# Multisociety guidance for infection prevention and control in nursing homes

**DOI:** 10.1017/ice.2025.10252

**Published:** 2025-11

**Authors:** Lona Mody, Sonali D. Advani, Muhammad Salman Ashraf, Allison H. Bartlett, Suzanne F. Bradley, Deborah P. Burdsall, Jennifer A. Hanrahan, Susan S. Huang, Robin L.P. Jump, Lindsay Nicolle, Mary-Claire Roghmann, Patricia Stone, Rekha K. Murthy

**Affiliations:** 1 University of Michigan and VA Ann Arbor Healthcare System, Ann Arbor, MI, USA; 2 Duke University School of Medicine, Durham, NC, USA; 3 University of Nebraska Medical Center, Omaha, NE, USA; 4 Comer Children’s Hospital, University of Chicago Medicine, Chicago, IL, USA; 5 VA Ann Arbor Healthcare System, University of Michigan Medical School, Ann Arbor, MI, USA; 6 Baldwin Hill Solutions, LLC, Palatine, IL, USA; 7 Eastern Virginia Medical School, Norfolk, VA, USA; 8 University of California Irvine School of Medicine, Irvine, CA, USA; 9 Geriatric Research Education and Clinical Center (GRECC) at the VA Pittsburgh Healthcare System, University of Pittsburgh School of Medicine, Pittsburgh, PA, USA; 10 University of Manitoba, Winnipeg, MB, Canada; 11 Baltimore VA Medical Center, University of Maryland School of Medicine, Baltimore, MD, USA; 12 Center for Health Policy, Columbia University School of Nursing, New York, NY, USA; 13 Cedars-Sinai, University of California Los Angeles School of Medicine, Los Angeles, CA, USA

## Abstract

This multisociety guidance was endorsed by SHEA, APIC, IDSA, PALTmed, and AGS. It provides recommendations for infection prevention and control (IPC) in the context of the complexity of nursing home care in the United States: increased medical acuity of residents, the spread of multidrug-resistant organisms, and the threat of emerging pathogens. Recommendations and implementation suggestions address IPC leadership, staffing, and resources, healthcare personnel and residents‘ adherence to precautions and effective hand hygiene, outbreak preparedness, training, occupational health, cleaning and disinfection in the care environment, and the involvement of IPC in the facility. The guidance also addresses the challenges of maintaining a home-like care space while sustaining necessary IPC measures. The guidance covers the role of regulatory bodies like the Centers for Medicare and Medicaid Services (CMS) and recommendations from the Centers for Disease Control and Prevention (CDC). It should serve as a resource for IPC program leaders in nursing homes who are aiming to enhance infection prevention efforts.

## Purpose

This guidance document updates the 2008 Society for Healthcare Epidemiology of America (SHEA)/Association for Professionals in Infection Control and Epidemiology (APIC) guideline: *Infection Prevention and Control in the Long-Term Care Facility* ^1^ and is intended to assist nursing homes in the United States (US) in defining and implementing their infection prevention and control (IPC) programs and practices.

## Background

Nursing homes, also called skilled nursing facilities, are an important provider of healthcare in the United States. The 2008 SHEA/APIC guideline provided a framework to define the structure and main elements for implementing a nursing home IPC program.^1^ The common infections occurring in nursing home residents and the viral and bacterial pathogens causing outbreaks in nursing homes described in that guidance remain quite relevant today. Furthermore, as seen during the COVID-19 pandemic, nursing home residents and healthcare personnel (HCP) experienced significant morbidity from outbreaks of SARS-CoV-2 introduced when virus was circulating in the community.^2^ While the basic principles for implementing a nursing home IPC program remain the same, facilities are challenged by the tasks of ensuring their IPC program’s infrastructure and practices evolve to meet the changing care needs of their resident population and responding to the emergence of antimicrobial resistance and novel pathogens. The Centers for Medicare and Medicaid Services (CMS) has made IPC in nursing homes a priority. Additionally, nursing homes must be attentive to and ready to adapt to changing recommendations by the Centers for Disease Control and Prevention (CDC) and standards by regulatory agencies.

The medical complexity and acuity of care provided in nursing homes has continued to significantly increase.^3^ Most individuals admitted to nursing homes come directly from acute care hospitals for the provision of skilled nursing or rehabilitative services, rather than primary residential care. These post-acute care residents experience risks of healthcare-associated infections and colonization with multidrug-resistant organisms (MDROs), such as exposure to indwelling medical devices, use of intravenous therapy, especially antibiotics, and presence of wounds, that are similar to those for hospitalized patients.^4,5^ In addition, a growing number of nursing homes have expanded their services to include specialty units for ventilator-dependent residents, further increasing the medical complexity and the risk of infection and MDRO colonization.^6,7^

A key component to reducing risks for nursing home residents, especially among those who require higher acuity care, is the incorporation of core IPC practices (e.g., hand hygiene, Standard Precautions, cleaning and disinfection of equipment) during resident care activities.^8^ Residents, HCP, and visitors may worry that IPC measures, including the placement of supplies and resources in resident rooms and care areas, will undermine attempts to maintain a “home-like environment” for residents.^9^ Additionally, prolonged restrictions on in-person visitation during the COVID-19 pandemic likely contributed to reductions in the physical and psychological well-being of the residents.^10^ However, IPC measures taken to provide safe care and reduce the spread of infectious pathogens are critically important to maintaining a resident’s health and quality of life.

Nursing homes should support a culture that prioritizes safety and emphasizes that everyone has a role in promoting infection prevention. Successful strategies include engaging frontline HCP in identifying solutions to ensure IPC practices during resident care, creating a culture of shared accountability for IPC outcomes, educating residents and their families, and supporting the use of innovative IPC improvement activities.^9^

Since the early 1990s, CMS regulatory standards for nursing homes have required nursing homes to have IPC programs.^11^ The effectiveness of these requirements has been variable, with lapses of IPC practice remaining among the most commonly cited deficiencies in US nursing homes from 2000 to 2007.^12^ In 2016, nursing home regulations underwent substantial updates, including new requirements for nursing home IPC programs that included implementing new antimicrobial stewardship activities and designating an infection preventionist with specialized training in IPC to be responsible for the IPC program.^13^ An evaluation of IPC programs comparing nursing home survey data from 2014 and 2018 showed promising improvements in implementation of specific IPC practices following the release of the new regulations^14^; however, the regulatory assessments of IPC programs in nursing homes across the country remain variable, raising concerns about the consistency and effectiveness of oversight from state to state.^15^

In the midst of the changing clinical landscape and growing regulatory expectations for nursing home IPC programs, a proposal was made to update the 2008 SHEA/APIC guideline: *Infection Prevention and Control in the Long-Term Care Facility* in order to support nursing homes’ IPC programs and implementation of practices based on a review of currently available evidence, taking into consideration the unique population and type of care performed in these facilities.

## Intended use

Although there is a broad spectrum of post-acute and long-term care providers, including long-term acute care hospitals, home health agencies, residential care facilities, and group homes, the primary audience for this guidance are leaders of IPC programs in US nursing homes. The principles outlined in this guidance could be applicable to other residential care settings, such as assisted living communities or intermediate care facilities; however, there may be unique considerations for the needs of individuals served by or the knowledge and experience of those working in those care environments that should inform the implementation of an IPC program’s activities.

No guideline or expert guidance document can anticipate all clinical situations, and this guidance document is not meant to be a substitute for individual clinical judgment by qualified professionals.

## Methods

### Document development

This guidance follows the process outlined in the 2017 “Handbook for SHEA-Sponsored Guidelines and Expert Guidance Documents.”^16^ While updated in September 2024,^17^ the guidance followed the 2017 Handbook until completion.^16^

IPC in nursing homes was among topics that were proposed and selected by the SHEA Guidelines Committee (GLC). The subsequent manuscript proposal developed by the GLC was approved by the SHEA Publications Committee and the SHEA Board of Trustees.

SHEA develops expert guidance documents for topics important in the provision of safe, effective healthcare, but that lack the level of evidence required for a formal guideline developed using the Grading of Recommendations Assessment, Development and Evaluation or a similar systematic methodology. Expert guidance documents are based on synthesis of available evidence, theoretical rationale, current practices, practical considerations, writing group opinion, and consideration of potential harm where applicable. As such, evidence level is not provided for individual recommendations.^16^

The writing panel developed PICO-style (population, intervention, control, and outcomes) questions based on themes that they identified. These questions were used in the development of search terms (medical subject heading and text word) by a professional medical librarian. Both the questions and search terms were voted on by the panel until unanimous approval was achieved. Articles published from January 1, 2007, through December 31, 2021, were collected from PubMed, with the search run on May 14, 2021. Articles published from 1974 through May 17, 2021, were collected from Embase, with the search run on May 18, 2021. In January 2024, the PubMed search was rerun with updated dates to January 18, 2024.

Only English-language articles on human subjects were included. The abstract management software Covidence was used, and abstracts from the article yield were blindly screened by two authors for inclusion. Drs. Mody and Murthy resolved conflicts, and subgroups screened full texts of studies for inclusion in this document.

SHEA guidance documents are developed with a formalized process for reaching consensus (see Supplementary Material, Table 1 for a summary list of expert guidance recommendations and Supplementary Material, Table 7 for a quick reference for topics).

Consensus around recommendations and rationale is determined via an anonymous comment period. For this document’s recommendations, full consensus was achieved.

See Supplementary Material, Table 2 for a list of terminology and acronyms. See Supplementary Material, Table 3 for additional resources to support nursing home infection prevention and antimicrobial stewardship efforts.

Supplementary Material, Table 8 provides the search strategy, exclusion criteria, and PRISMA.

### Authors

The authors include current and past members of the SHEA Guidelines Committee, the SHEA Long-Term Care Special Interest Group, the American Geriatrics Society (AGS), and the Pediatric Infectious Diseases Society (PIDS), as well as representatives for APIC, the Post-Acute and Long-Term Care Medical Association (PALTmed; previously The Society of Post-Acute and Long-Term Care Medicine [AMDA]), and the Infectious Diseases Society of America (IDSA).

Dr. Robin Jump served as an author and representative for PALTmed, Deborah Burdsall and Patricia Stone served as authors and representatives for APIC, Dr. Suzanne Bradley served as an author and representative for IDSA. All authors served as volunteers. All authors participated in the development of and attested to agreement with the recommendations and rationale sections contained in this document.

### Review

The document was reviewed by the SHEA Guidelines Committee, the SHEA Publications Committee, APIC, IDSA, PALTmed, and AGS, and endorsed by the SHEA Board of Trustees, APIC, IDSA, PALTmed, and AGS.

## Recommendations for infection prevention and control (IPC) in nursing homes

### Infection prevention and control program (IPC program)

#### Leadership

**1. What resources (physical, human, financial) are needed to meet the goals of the nursing home’s IPC program?**

*Recommendation:* The resources for a nursing home’s IPC program should include:

1. At least one infection preventionist (IP) to manage the infection prevention and control (IPC) program who:
  - Has ongoing, specialized training in IPC that is financially supported by the nursing home
  - Demonstrates commitment to ongoing continuing education in IPC to remain current in developments and strategies to optimize the IPC program
  - Has clinical and/or public health experience
  - Is an effective communicator, educator, leader, mentor, and collaborator
  - Receives training in leading and managing programs
2. Sufficient dedicated time for the IP(s) to manage the IPC program based on the complexity of the resident population and services provided:
  - At least one full-time equivalent (FTE) IP, if the facility has more than 100 licensed beds or provides onsite ventilator or hemodialysis services
  - At least 0.5 FTE IP (20 IP hours per week), if the facility has fewer than 100 beds and does not provide on-site ventilator or hemodialysis services
3. Adequate staffing (e.g., nursing, clinical) and supplies (e.g., personal protective equipment [PPE], alcohol-based hand sanitizer [ABHS], US Environmental Protection Agency (EPA)-registered disinfectants) to allow healthcare personnel (HCP) to follow all recommended IPC practices
4. Dedicated time for personnel to receive regular job-specific IPC education and demonstrate competency through assessment (see **14** and **15**)
5. Access to information technology training and infrastructure (e.g., integrated electronic health records, software applications, internet access) to support facility-level surveillance activities and access to public health surveillance programs
6. Access to expert advice, learning collaboratives, and professional associations specific to IPC (see **6, 9,** and **42**).

*Rationale:* In 2016, the Code of Federal Regulations (483.80)^18^ required that all Centers for Medicare and Medicaid Services (CMS)-certified nursing homes have a designated person overseeing the IPC program. This person should be an IP with a scope of practice^19^ that includes IPC knowledge and skills and clear expectations to implement best practices. Although these practices should be tailored to the population and resources, they should also be built on core IPC practices outlined in CDC guidance and should be adherent to CMS requirements.

The individual overseeing the IPC program should have dedicated support from nursing home administrative and medical leadership, including the medical director, and the organization’s commitment to provide the financial resources to meet the growing scope of the IPC program. Program responsibilities include developing policies and procedures to implement the most current, evidence-based practices, using electronic systems for infection surveillance and reporting, informing HCP IPC training, auditing IPC practices, guiding outbreak preparedness, prevention, and response, promoting antimicrobial stewardship, participating in quality assurance activities, and leading quality improvement projects to improve IPC practices.

#### Staffing

Although no recent studies are available that quantify IP hours for all levels of nursing home care, a nursing home should provide adequate IPC personnel, time, and resources in accordance with the level of acuity and services provided to residents. For example, chronically ventilated nursing home residents are at greater risk for infection. Bundled interventions (a defined set of evidence-based practices) put in place by the IP may decrease rates of ventilator-associated pneumonia.^20,21^ Residents who are ventilated are at risk for high rates of MDRO colonization and may benefit from admission screening to assist with early identification and appropriate isolation,^22^ especially with the emergence of *Candida auris* and carbapenem-resistant *Acinetobacter baumannii* in nursing homes that provide ventilator services.^6,23–27^

Of note, specific staffing requirements for IPs may vary depending on the size and complexity of the facility, as well as the local and state regulations. The National Academies of Sciences, Engineering, and Medicine have recommended that the IP have sufficient time to perform required duties; one survey found approximately 25% of IP time is spent on infection surveillance.^28^ Submitting surveillance data to a network, which allowed for comparing infection rates across facilities and tracking performance improvement, has been associated with a decreased rate of healthcare-associated infections (HAIs), including influenza-like illness, urinary tract infections (UTIs), and pneumonia over time.^29^ This important and time-intensive responsibility is one of numerous responsibilities, including the IP’s engagement in continuing education and need to train and educate nursing home personnel in IPC.^30^

As part of guidance issued during the COVID-19 pandemic, CDC recommended at least one full-time IP for every 100 beds or for any nursing home that provides dialysis or mechanical ventilation.^31^ Some states legislate FTE IPs for each nursing home. In New Jersey, nursing homes with 100 residents or more must have a full-time IP in a manager position and each nursing home must have an IPC committee.^32^ California has legislated that each nursing home must have a full-time IP.^33^ In light of the evolving expectations for nursing home IPC programs and the increasing complexity of the resident population, nursing homes larger than 100 licensed beds and those that provide onsite ventilator or hemodialysis services should have at least one FTE IP. If a facility has fewer than 100 beds or does not provide these services, a nursing home should have at least 0.5 FTE IP.

#### Training

IPs who work in nursing homes should have specialized training in infection prevention and control. A national survey of US nursing homes found that facilities that have IPs with specialized IPC training (certification in infection control, state or local training course with certificate, national or local training course through a professional society, or other) were more likely to have recommended IPC policies in place, when controlling for other known measures of quality (e.g., ownership, staffing).^14^ Agarwal and colleagues also found a positive association between nursing homes that employ trained IPs who have certifications and nursing homes having a comprehensive antimicrobial stewardship program.^34^ Prior to the CMS regulation for IPC, Wagner and colleagues did not find a specific correlation between IPC training and nursing home quality measures. However, this correlation could strengthen as more directed training and efforts to build a foundation for IPC in the nursing home occurs.^35^ This group did find that having a FTE IP was associated with better outcomes in 3 of 8 quality measures (percent high-risk residents with pressure ulcers, percent influenza vaccination for long-stay residents, and percent influenza vaccination for short-stay residents).

The National Academies of Sciences, Engineering, and Medicine recommends that every nursing home has an IP who is a registered nurse (RN), advanced practice RN, or a physician^36^ and must have received specialized IPC education. However, other research has found that the effect of specialized training was not dependent on whether the IP was a RN. Therefore, since many IPs come from public health and other clinical backgrounds, the IP position does not need to be limited to only a RN or physician.

#### Resources

A business case should be used as a tool to support advocating to nursing home leadership for resources.^25,27^ For IPC programs to be successful, the nursing home should have adequate IP and nurse staffing to reduce rates of infections,^24^ which often requires programs to improve retention of personnel. The nursing home should ensure that ample quantity of IPC supplies (e.g., PPE, ABHS, EPA-registered disinfectants) is available in resident care areas to allow HCP to follow all recommended IPC practices. The IP also needs access to information technology training and infrastructure (e.g., integrated electronic health records, software applications, internet access) to support collection and management of infection surveillance and other resident outcome data used to inform performance improvement.^24,37–39^ Technologic resources not only support data collection and management within the facility but also support a nursing home’s access and connectivity to regional population and laboratory-based public health surveillance programs.^40–43^

**2. To whom should the nursing home IP report?**

*Recommendation:*

1. The nursing home IP should report to a designated person in administrative and medical leadership who has knowledge relevant to regulatory and resource needs for the IPC program.
2. The IP should be a member of the Quality Assessment and Assurance (QAA) committee to integrate IPC activities within the quality assessment and performance improvement programs.
3. To be successful, IPC programs require visible and tangible support from all levels of nursing home personnel:
  - Administrative and medical leadership, including the medical director, should actively participate in IPC program activities to provide appropriate resources and training to support the implementation of IPC policies and procedures
  - Nursing homes should clearly define the IP position and include dedicated time for the IP in IPC training, continuous education, and modes of communication with facility personnel, including leadership
  - Nursing homes should evaluate IPC program surveillance reports and practices using the Quality Assurance Performance Improvement (QAPI) process.

*Rationale:*

#### Administrative and medical leadership and reporting structures

The nursing home should ensure that there is structured oversight of the IPC program with support for activities from administrative and medical leadership, including the medical director. Administrative leaders should provide financial resources to meet the training and continuing education needs of the IP and to procure supplies needed to implement IPC program activities. Medical leadership, including the medical director, should provide clinical insight into protocols, processes, and treatment needs and should be an active participant in the IPC program and regularly attend its meetings.^1,44^

Reporting structures for the IPC program and the ways that administrative and medical leadership engage may vary depending on the facility’s needs. In a review article, Montoya and colleagues suggested that facilities designate an administrator to be an active part of the team led by an IP.^45^ In a qualitative study conducted in Canada during the COVID-19 pandemic, Yau and colleagues found that communication and coordination among leadership and personnel were essential to manage pandemic needs, in some instances involving daily huddles.^46^ In a successful model described by Bartels and colleagues, the nursing home IP reported to a regional director of infection prevention in a large healthcare system.^47^ With growing numbers of nursing home corporations, when available, IPC or quality leaders at the regional or national level of an organization should support and collaborate with nursing home IPs to develop standardized policies and practices.^27^ Regardless of configuration, the IP and IPC program should have a reporting structure that facilitates communication with the medical director and leadership of relevant departments, including mechanisms to seek resources for the IPC program.

#### QAA committee, QAPI program, and IPC committee

In the United States, CMS requires that each nursing home facility maintain a QAA committee that meets at least quarterly and includes the director of nursing, the medical director or designee, at least three other staff members (one of whom must be in a leadership role), and the IP.^18,48^ CMS also requires that each facility conduct an annual review of the IPC program and update the program as necessary. Given that CMS requires the IP to report to the QAA committee, the members of the QAA committee are well positioned to review IPC policies. The IP should present and review the IPC policies with the QAA at least annually, discussing those for which modifications may be indicated due to internal data, new evidence, or changes to local, state, or federal guidance. The IP is responsible for documenting policy changes and associated dates, which will support the annual review process.

The QAA committee implements the QAPI program, intended to be a comprehensive, data-driven process to address outcomes of care and quality of life. Part of the QAPI program’s mandate is to establish and implement written policies and procedures for feedback, data collection systems, and monitoring, all aligning with the activities of an effective IPC program. Although IPC should be integrated into QAPI, it is not the only safety and quality concern addressed by the program. Therefore, some nursing homes may decide to create a separate interdisciplinary IPC committee to focus on implementing the core activities of the IPC program. Even if an IPC committee exists, the IP should continue to serve on the QAA committee to generate support and resources for the IPC program. A strong synergistic relationship between a nursing home’s IP and the facility’s administrative and medical leadership, including the medical director, enhances alignment, adherence to IPC practices, and evaluation for additional changes presented for QAPI.

**3. How can a nursing home support the continuity of its IPC program?**

*Recommendation:* Nursing homes should implement strategies to retain and mentor HCP for IPC program continuity so that the IPC program is not dependent on one individual:

1. Prioritize and invest in personnel retention strategies, including competitive wages and benefits for the IP
2. Provide ongoing, job-specific IPC training due to the likelihood that turnover of HCP leads to decreasing effectiveness of the IPC program (see **14** and **15.**)
3. Establish a mentoring program to foster interest in IPC:
  - Identify individuals who participate in quality improvement initiatives and/or demonstrate interest in IPC in interactions with nursing/clinical supervisors
  - Provide incentives for both mentors and mentees
4. Encourage and support participation in public health activities, local Association for Professionals in Infection Control and Epidemiology (APIC) Chapter meetings, and educational offerings from the Society for Healthcare Epidemiology of America (SHEA) and other professional organizations that work in IPC
5. Develop processes for succession planning, transitions, and cross-training for the activities that support the IPC program (e.g., conducting surveillance, developing IPC policies, implementing antimicrobial stewardship)
6. Reduce decision fatigue with checklists and standard processes
7. Celebrate success and foster a team atmosphere.

*Rationale:* A nursing home should have a plan in place to ensure the continuity of the IPC program activities and stability in the IP position. In a study that evaluated nursing home IPC program characteristics linked with IPC deficiency citation data, nursing homes that received citations reported having IPs with less IPC experience in their facility and were less likely to provide financial resources for continuing education in IPC.^49^ Fu and colleagues found that IP turnover every 3 years or more was associated with a less comprehensive nursing home antimicrobial stewardship plan.^34^

Although not specifically assessed in nursing homes, understanding the effects of financial factors, such as competitive wages and benefits and professional development support (e.g., investments in training opportunities), could inform efforts to recruit and retain IPs.^50,63^ Additional strategies to develop and support IP staff identified through a survey of IPs across various healthcare settings include mentoring new IPs, systems to support healthy work environments, reward and recognition programs, and systems to assess and balance workload.^51^

Another issue that affects quality of care in nursing homes is instability among certified nursing assistants (CNAs) and nursing staff.^52^ High turnover levels among nursing personnel in nursing homes (>70% per year) are associated with decreased ”compliance” of vital sign monitoring, physician notification, and appropriate hospitalization for pneumonia^24^ and increased numbers of pressure ulcers, pain, and UTIs.^53^ Qualitative studies of CNAs identified several factors associated with decreased “intent to leave”: satisfaction with wages, benefits, and advancement opportunities and having a respectful nursing supervisor who was willing to help when needed.^54^ Temple and colleagues found that involvement in resident care planning decreases the odds of high CNA turnover.^55^

Establishing processes for succession planning and cross-training HCP on IPC activities are also important for the continuity of the IPC program. Steps to mitigate the effects of high turnover on IPC practices include planning for continuous, ongoing educational efforts.^56^ Simple, regularly updated procedural documents that walk nursing home HCP through the steps of specific care activities (e.g., tracheostomy suctioning) can help decrease variability of practice.^57^

### Risk assessment

**4. How should a nursing home perform an IPC risk assessment?**

*Recommendation:*

1. The nursing home should perform a risk assessment annually to determine the resources needed to identify and reduce the risk for infections among residents and HCP.
2. The nursing home should assess IPC risk factors at the following levels:
  - Resident-level (person), such as ventilator use or the presence of an indwelling catheter or other medical device
  - Process-level (intervention), such as HCP compliance with hand hygiene, vaccination, and PPE use
  - Facility-level, such as location, access to services, and physical infrastructure.

*Rationale:* An IPC risk assessment is a systematic process that evaluates a setting for potential hazards and gaps in IPC practices. Nursing homes should conduct risk assessments once a year in accordance with CMS regulations^18^ to identify IPC priorities, to allocate resources (e.g., HCP training, supplies) to address IPC risks, and to plan quality improvement activities. One approach to conducting a risk assessment is evaluating IPC risk factors based on 3 categories: residents, processes, and facility infrastructure.

At the resident level, an assessment should consider an individual’s risk of infections based on the types of care and services provided by the facility. For example, facilities that care for residents on ventilators may need to dedicate more resources to addressing risk of infections from respiratory therapy practices, equipment, and prolonged exposure to medical devices. Facilities that care for residents with dementia or memory impairment may need to establish an environmental cleaning program that takes into account the increased environmental contamination from residents who are unable to follow instructions on hygiene practices and may have a higher prevalence of multidrug-resistant organisms (MDROs).^58^ Facilities that care for post-surgical patients may need additional training in incision and wound care.

Process-level factors consider knowledge or resource gaps that could affect HCP adherence to practices known to prevent transmission of pathogens, such as hand hygiene, employee vaccination, and PPE use.^59^ Device use and handling and removal policies and practices are relevant process factors because devices have been associated with an increased risk of infection and MDRO colonization.^60^

Facility-level factors, such as geographic location, could affect resources needed for detection and prevention of infections among residents. For example, nursing homes in a community experiencing the emergence of highly resistance MDROs in local healthcare facilities should be aware of their access to laboratories (e.g., state/regional public health laboratories) with capacity to perform specialized testing for resistance and assist in performing surveillance cultures, when indicated.^61^ To prepare for increasing rates of viral respiratory infections in the surrounding community, a nursing home should evaluate whether their laboratory offers diagnostic tests that provide rapid results for influenza and SARS-CoV-2.^62,63^ Alternatively, the nursing home may continue to maintain Clinical Laboratory Improvement Amendments (CLIA) Certificates of Waiver to perform point-of-care testing for SARS-CoV-2 and other respiratory viruses.^64^ Another facility-level factor for nursing homes to consider as part of the annual IPC risk assessment is the infrastructure of the building (e.g., heating, ventilation, and air conditioning [HVAC] and water systems) to ensure they are maintained and optimally functioning.

Modifiable tools like those found in the CDC Nursing Home Infection Preventionist Training Course^65^ provide a template for the stepwise evaluation of resident-level factors to develop numeric risk level scores for infection events based on probability of occurrence, level of harm from event, impact on care, and readiness to prevent. A similar numeric risk level score for IPC practice failures, used to identify resource gaps that may affect adherence to IPC practices, is based on probability of occurrence, impact on resident and HCP safety, capacity to detect, and readiness to prevent. Through the IPC risk assessment, the IPC program can prioritize activities to target areas that receive higher scores.

To further evaluate factors related to facility infrastructure, nursing homes may also conduct an evaluation of the safety of the physical environment. For example, while walking through different areas of the facility, the IP can assess cleanliness and potential safety concerns in resident rooms, treatment areas, and shared areas; monitor availability of IPC supplies (e.g., PPE, cleaning, and disinfection products); and review building maintenance activities.

### Working partners

#### Internal working partners

**5. How should the nursing home’s IPC program engage with facilities management?**

*Recommendation:* To proactively manage potential IPC concerns, particularly as they relate to water management, airflow, air filtration, air disinfection, and construction, the IPC program should provide expert input and/or consultation to the facility management team, which may include a facility engineer, maintenance director, and/or industrial hygienist.

*Rationale:* The IPC program should develop a close working relationship with its facilities management team to ensure building systems are maintained and optimally functioning, and support collaborative responses to unexpected events, such as broken equipment or a leak in a roof. The IPC program should also be engaged by facilities management during the planning and execution of any construction projects. At a minimum, the IPC program should work with facility management to be familiar with water management and HVAC systems.

*Water.* The primary concern regarding water systems is the potential for waterborne pathogens, such as *Legionella* spp. and *Elizabethkingia* spp., which can colonize water environments and, through aerosolization, aspiration, or direct contamination, cause respiratory infections.^66^ Water systems such as taps, aerators, drains, and faucets also can be a reservoir for other pathogens, including MDROs.^66,67^ CDC, based on data from 15 acute and 57 long-term care settings, estimated a case fatality rate of 25% for definite and 10% for possible healthcare-associated Legionnaires’ disease.^68^ The facility management team, including the maintenance department, and the IP should develop a water management program^69^ that ensures water quality and safety. The water management plan should include active monitoring of water systems through visual inspection of water tanks, pipes, sinks, hoppers, and equipment, and routine monitoring of temperature and disinfectant levels in premise plumbing (including wastewater plumbing) to support a safe water supply that is inhospitable to pathogens while avoiding the risk of burns to residents.

*Airflow and ventilation.* Respiratory outbreaks in congregate care settings can be caused by multiple organisms, but mainly viral respiratory pathogens including influenza, respiratory syncytial virus (RSV), human metapneumovirus, adenovirus, parainfluenza, and SARS-CoV-2. Proper ventilation is critical to efforts to prevent the spread of these viruses and others, including more rarely, varicella-zoster virus, which can cause shingles. The IPC program should play a role in enhancing and maintaining building air quality (see **28**). For example, when building a new nursing home or an extension to an existing facility, IPC should be involved in considering whether to install a ventilation system that allows for negative pressure to be maintained in certain rooms when needed.^70^

*Construction.* Nursing homes are required to maintain a “safe, sanitary, and comfortable environment.”^18^ The IPC program’s involvement in construction is well understood in acute care but may be less established in nursing homes. Especially during the COVID-19 pandemic, evidence emerged about the increased infection risk of aging infrastructure.^36,71^ With calls for renovations and improvements to nursing home design, there is an increased need for the IP to coordinate with and provide input to facilities management to prevent potential construction-related infections.

#### External working partners

*Consultants*

**6. What should nursing homes consider when deciding whether to hire an infectious disease or IP consultant?**

*Recommendation:* Nursing homes may consider hiring external infectious diseases or IP consultants if the IPC risk assessment (see **4**) reveals resident-level risk factors or process-level practice implementation gaps that require additional expertise.

*Rationale:* CMS requires that nursing homes conduct a facility-wide assessment at least annually to determine what IPC resources are necessary to provide safe care for its residents during both day-to-day operations and emergencies. This assessment considers the probability of infections based on the resident population, risk of IPC practice gaps based on personnel competencies and expertise, and facility resources. Although IPs have received specialized training, persistent turnover can compromise their operational experience. The facility assessment and gap analysis should consider the experience and expertise of current personnel involved in IPC programs. A 2015 survey indicated that IPs in nonacute care settings, including nursing homes, had limited experience in IPC; only 9% were certified in infection control.^28^ Clinical experiences of infectious disease and IP consultants can support IPC efforts. External consultants may help less experienced IPs conduct their facility assessment and suggest specific programs to address the identified needs, such as hand hygiene monitoring or standardized protocols to help decrease the use of urinary catheters.

In evaluations of whether to engage external infectious diseases or IP consultants, other considerations include the availability of relevant IPC data to inform areas for program improvement, internal expertise for data analyses, desired outcomes and goals for the program, available methods for sharing information securely with consultants, the medical complexity of nursing home residents, and the financial resources available to the facility. Defining the goals and resources for working with an external consultant will inform decisions about the specific services (e.g., in-person versus remote consultations; day-to-day engagement or periodic reviews; program development with or without subsequent maintenance) and resources needed (e.g., access to electronic health records; video equipment and communication systems compliant with the Health Insurance Portability and Accountability Act; support around licensure, contracting, and billing procedures).

The nursing home can also obtain support through collaborations with experts from the local community, hospitals within their referral network, and state/local public health programs (see **9**). Such partnerships may be mutually beneficial with shared goals and logistical advantages (e.g., avoiding hospital readmissions, having decreased prevalence of MDROs and *Clostridioides difficile* [*C. difficile*] infections across institutions, having onsite consultations and opportunities for in-person education).^72^

**7. How should the IPC program engage with the hiring and responsibilities of contract services?**

*Recommendation:* Nursing homes should involve the IPC program in:

1. Identifying IPC risks related to the proposed services (e.g., wound care, podiatry)
2. Participating in hiring considerations and defining contractors’ responsibilities
3. Educating contractors about IPC policies and protocols
4. Monitoring contracted services’ compliance with IPC protocols.

*Rationale:* Lapses in IPC practices by contractors, such as inadequate cleaning and disinfection of shared equipment, have led to transmission of infections within nursing homes. Wise and colleagues described a hepatitis B virus outbreak affecting 5 of 15 residents who received podiatric care in a single day.^73^ A hepatitis C virus outbreak involving 45 residents was found to be related to contracted podiatry and phlebotomy services.^74^

The IPC program should always be involved with decisions to hire contractors to evaluate whether the proposed contracted services may increase infection transmission risks within the facility and, if yes, whether contractors have an adequate plan in place to mitigate those risks. Additionally, the IPC program should develop training for contractors on the facility-specific policies and protocols that should be followed while working at the facility. These include general principles related to Standard Precautions (e.g., hand hygiene, respiratory hygiene) and Transmission-Based Precautions, including, when appropriate, targeted education around donning and doffing PPE, wearing masks appropriately, and other activities relevant to a particular service.

Nursing homes should make sure that contractors who provide services that require equipment reprocessing (e.g., podiatry, dental, wound care services) are performing sterilization and disinfection per the equipment or device manufacturer’s instructions for use (MIFU) and are maintaining records. Preferably, the contractors should bring an adequate supply of clean and disinfected or sterilized instruments to provide safe care for all residents requiring the services.

The nursing home facility should make sure that contractors have appropriate space available in the building to provide the medical services safely. The treatment area should also have adequate supplies and access to PPE and ABHS. Steps should be in place to make sure that separation is maintained between clean and soiled equipment to prevent cross contamination. When possible, single-use, disposable equipment should be used, with a process in place for proper disposal after use. If a contractor uses reusable medical equipment to provide services, then processes should be in place for safe preparation and transport of the equipment for appropriate processing before use on anyone else. Finally, the IPC program should perform periodic audits to ensure compliance with the facility’s protocols related to the contracted services and provide appropriate feedback.

**8. How should nursing homes verify IPC training and vaccination status for contract employees and consultants?**

*Recommendation:* Nursing homes should ensure that the contract and onboarding processes for all contract employees and consultants include provisions requiring appropriate documentation of IPC training and vaccination status.

*Rationale:* Contract employees and consultants should follow the same IPC training and occupational health vaccination requirements that are applied to HCP who are directly hired by the nursing home. This expectation should be made clear at the time of contract negotiations and included in the contract itself. The contract should also include the provision for a contractor to share appropriate documentation verifying training and vaccination status for employees. If the contractor assumes responsibility for ensuring compliance with the facility vaccination requirements for employees, the contract should clearly state that appropriate documentation verifying the vaccination status of the contract employees will be shared with the nursing home. Similarly, if the contractor provides IPC training to employees, the IPC program may need to perform an assessment to ensure that the training meets the nursing home’s requirements before acceptance. Even if the specific training is acceptable, the nursing home should provide contract employees with additional orientation and education on facility-specific IPC protocols (e.g., the use of signage in the facility for Transmission-Based Precautions, proper use of facility-approved disinfectants including contact time). The nursing home’s IPC program should develop an internal process to keep track of IPC training and vaccination status of all the contract employees and to follow up with the contractors if any information is missing.

**9. What relationship should nursing homes have with local and state public health departments?**

*Recommendation:* Nursing homes should:

1. Develop a relationship with local/regional and state public health departments for support, guidance, and collaboration with HCP within the local and regional healthcare continuum.
2. Comply with reporting of cases and outbreaks of infectious diseases as required by local and state public health departments and institutional jurisdictions. Public health departments can help facilities in their efforts to prevent and control pathogen transmission.
3. Partner with public health departments, local hospitals, and other healthcare organizations in quality improvement and safety collaboratives to support antimicrobial stewardship, to prevent infections, outbreaks, and the spread of MDROs, and to improve resident outcomes.

*Rationale:* State and local public health departments assist nursing home IPs in myriad ways by providing education, resources, and support for the implementation of IPC program activities. Public health department programs focused on prevention of HAIs and spread of antibiotic resistance have created online and in-person IPC trainings, templates, and tools to support infection surveillance, outbreak response, and antimicrobial stewardship and have provided remote and onsite technical assistance for the nursing home IP.^75–79^ An evaluation of the effect of various IPC activities led by state public health departments found that nursing homes in states offering IPC training for providers had fewer IPC-related deficiency citations.^80^

A relationship between a nursing home IP and the local public health department can improve communication and information sharing when the IP identifies questions related to IPC practices or reports a suspected outbreak to the appropriate public health authority in the region. When nursing homes report infection outbreaks, public health department staff can support nursing homes with collection of case data and interpretation of findings, including patterns of infections, laboratory results, observed symptoms, and surveillance definitions, and can offer strategies to disrupt ongoing transmission. Public health department programs can also facilitate opportunities for nursing homes to collaborate with community partners, including local hospitals and quality improvement organizations, to improve resident care and outcomes.^72,81^

Members of the nursing home’s IPC program should have a clear understanding of the structure of their local and state public health jurisdictions to access support. Each jurisdiction may have a different reporting structure. Many corporate nursing home structures cross multiple state and local jurisdictions, meaning that corporate guidance may not pertain to local or regional public health requirements.

**10. What IPC-specific information should be communicated during resident/patient transfers?**

*Recommendation:* Nursing home HCP involved in resident transfers to or from hospitals, emergency departments, and primary care settings should be proficient in communicating and receiving IPC-specific information, including the resident/patient’s:

1. History of colonization or infection with MDROs
2. Relevant microbiological data, including cultures and susceptibilities
3. Pending test results
4. The need for and type of Transmission-Based Precautions
5. The presence of indwelling medical devices, wounds, diarrhea, or uncontained secretions
6. Current skin conditions
7. Recent or current antimicrobial exposure
8. Vaccination status for relevant vaccines (e.g., influenza, pneumococcus, COVID-19).

*Rationale:* The goal of a successful interfacility transfer is to ensure a safe handoff for a patient/resident moving from one healthcare facility to another as the care needs change. Having relevant information about risk factors and history of infection or colonization with germs that spread from person to person will ensure a safe environment for both the individual moving to a new care setting and the HCP receiving them.

Structured, systematic, and bidirectional communication around transitions of care is essential to patient safety. Ideally, communication about residents/patients as they transfer between healthcare institutions should occur among HCP with similar roles (i.e., nurses, pharmacists, and physicians). With the increasing focus on regional integration of healthcare systems, the nursing home may want to coordinate with their key referral hospitals to implement bidirectional, interfacility communication activities.^42,82^ Among resources available to support IPC programs in healthcare, CDC has an example of an interfacility transfer form that can be used to communicate IPC information.^83,84^

### Occupational health

**11. How should nursing homes prevent the transmission of infectious illness from HCP to residents and other HCP?**

*Recommendation:*

1. Nursing home personnel and individual HCP, including contractors, consultants, and others who enter the nursing home but may not be directly employed by it, are responsible to adhere to federal, state, and local requirements concerning:
  - Vaccinations:
    - HCP should receive recommended vaccinations or have documented evidence of immunity against vaccine-preventable diseases
    - Nursing homes should:
      - Enforce vaccination policies in keeping with vaccine recommendations, including exemptions for medical contraindications and those specified by state and federal regulations
      - Track vaccination status of HCP (see **15** and **16**)
      - Utilize programs and resources to improve vaccine uptake (see **12**)
  - Reporting to public health authorities when an illness identified in the nursing home or among HCP has public health implications or is required to be reported (see **9**).
2. Nursing homes should implement policies and processes and that:
  - Promote timely reporting by HCP of signs, symptoms (e.g., fever, cough, diarrhea, vomiting, draining skin lesions), or diagnosed illnesses that may represent a risk to residents and other HCP
  - Support HCP with acute infectious illness to adhere to work restrictions to prevent spread of illness to others in the facility.

*Rationale:* Presenteeism, working while ill, is estimated to be one of the biggest costs to companies in terms of lost productivity and can contribute to errors.^85^ In the nursing home setting, presenteeism can lead to greater consequences, as residents with underlying comorbidities risk becoming ill from exposure to HCP who are sick. HCP have self-reported high rates of working while ill and outbreaks have occurred as a result.^86–89^ Factors that contribute to presenteeism include behavioral norms for a given facility, negative consequences of missing work, and negative views toward those who call off work.^90^ HCP have cited reasons for presenteeism that include a sense of moral obligation to work, especially when their place of work is understaffed,^91^ not being able to afford lost pay, and holding the perception that they are able to continue to perform their duties while ill.^87^

Prevention of vaccine-preventable respiratory infections has the greatest potential impact on presenteeism. Presenteeism is also reduced when facilities have sick leave policies that encourage people not to work when ill and that emphasize the need to isolate sick individuals.^92–94^ CDC guidelines describe infrastructure and practices for occupational IPC.^95^

**12. How can nursing homes increase vaccine coverage among HCP?**

*Recommendation:* Nursing home administration and medical leadership, including the medical director, should:

1. Identify and implement multimodal interventions to increase HCP acceptance of CDC-recommended vaccines
2. Consider the use of educational campaigns and strategies such as onsite delivery of vaccines, time off for receiving and recovering from vaccination, and other ways to promote vaccine uptake and to improve vaccine confidence.

*Rationale:* To reduce vaccine-preventable morbidity among HCP and residents and to decrease the likelihood of transmission of vaccine-preventable illnesses, the CDC recommends that all HCP receive recommended vaccines.

#### Barriers to vaccination

There is large body of evidence detailing barriers that may deter HCP from getting vaccinated. Nursing homes should implement and evaluate programs that address misconceptions about vaccination, especially in areas with low vaccine uptake.^96^ Education and in-services can be rolled out using brief unscheduled sessions. In one study, 15-minute chance encounters and unplanned in-services helped deliver the message of importance of influenza vaccination and led to an increase in vaccine uptake. This principle can apply to other proven measures, such as hand hygiene and PPE use.^97^

Nace and colleagues reported that turnover of HCP was a barrier to vaccination uptake.^98^ Kenny and colleagues found lack of encouragement from peers and failing to perceive the vaccination as rewarding were significant predictors of HCPs not intending to get vaccinated.^99^ Using US cross-sectional survey data, Groenewold and colleagues found that not feeling respected/rewarded for work was negatively associated with vaccination.^100^

In Michigan, Kimmins and colleagues found misconceptions or lack of knowledge were the most frequently cited barriers to vaccinations.^101^ In a cross-sectional study in Tuscany, however, Domnich and colleagues found that confidence in vaccination was the strongest predictor of uptake and that health literacy was not a predictor.^102^ Lorini and colleagues found that people’s perceptions of risk versus value and the norms of their social groups affected vaccine uptake.^103^

#### Interventions to increase uptake

Numerous researchers have reported that interventions that are tailored and multifaceted improve vaccine uptake. In 2010, Lam and colleagues published a systematic review of 12 eligible studies investigating campaigns to improve HCP vaccination in nonhospital settings, including nursing homes, and found that campaigns with a greater variety of components (e.g., education, better access, role models, legislation or regulation) were the most effective.^104^ Ofstead and colleagues tested customized multifaceted interventions in four facilities in the Midwest and found HCP vaccination uptake increased from 50% to 85% (*p* < 0.01) and absenteeism due to respiratory illness decreased from 31% to 19% (*p* < 0.01) in the year after the intervention.^105^ Looijmans-van den Akker and colleagues conducted a cluster randomized controlled trial in 33 Dutch nursing homes to assess the effects of a systematically developed multifaceted intervention program to improve influenza vaccination uptake in HCP.^106^ The program, which included vaccine promotional materials (e.g., posters), education and personalized communication (letters to HCP), hosting informational meetings to address HCP questions, and identifying vaccine role models, produced significantly higher vaccination rates, but uptake remained moderate. Using materials designed to facilitate the implementation of similar interventions, additional studies found comparable results.^107,108^ In July 2021, CMS-certified nursing homes reported 82.3% of residents and 60.8% of HCP had been fully vaccinated against COVID-19 (84.8% of residents and 62.6% of HCP had been partially vaccinated).^109^ The authors concluded that facilities should use communication resources developed to increase vaccine confidence among HCP and strategies to address structural barriers, such as scheduling or paid medical leave for possible postvaccination side effects.

Black and colleagues found that influenza vaccination coverage was highest (94.8%) among HCP in settings where vaccination was required, although during the 2017-2018 influenza season, only 29.6% of nursing home HCP reported their facilities having a vaccination requirement.^110^ The study also found that influenza vaccination coverage was lowest for nursing assistants and aides in nursing homes compared with other HCP. In France, Elias and colleagues found that better knowledge about influenza prevention tools was correlated (*p* < 0.001) with higher influenza vaccination coverage in HCP. Individual perceptions of vaccination benefits also had a significant influence on the coverage (*p* < 0.001).^111^ Similar findings were reported among nursing home HCP in Ireland.^99^

### Healthcare-associated infection (HAI) surveillance

**13. How should a nursing home IPC program decide which symptoms, syndromes, and microorganisms to include in its surveillance program?**

*Recommendation:* Nursing homes should:

1. Establish priorities for routine surveillance of HAIs in the nursing home based on the needs of the facility, community risks, and regulatory requirements
2. Adopt standardized definitions and methods of reporting for HAI surveillance.

*Rationale:*

#### Priorities for routine surveillance

A primary goal for nursing home IPC programs is to improve resident care through the prevention of pathogen spread and reduction of HAIs. To accomplish this, the IP in conjunction with the QAA committee should have a written surveillance plan outlining which HAIs will be routinely monitored and reported for quality improvement and/or regulatory purposes. HAI surveillance is used to set goals for the IPC program’s prevention activities and should be reassessed on at least an annual basis.

Selecting HAIs to include in the surveillance plan may involve several strategies, including identification of specific pathogens (e.g., *C. difficile*), antimicrobial resistance patterns (e.g., carbapenem-resistant), and clinical syndromes consistent with transmissible infections (e.g., influenza-like illness or gastroenteritis). The IPC risk assessment can inform the HAI surveillance plan based on specific resident populations, device use, and antibiotic use.

Facilities should prioritize HAIs that link to IPC prevention goals that can be reliably assessed and achieved (e.g., improving central line accessing and dressing care to reduce central line-associated bloodstream infection [CLABSI]). A routine surveillance plan should address transmissible, preventable infections with high morbidity, mortality, or impact on the quality of life for residents.^112^

The US Department of Health and Human Services (HHS) Long-Term Care Facility National Action Plan identified several infection surveillance and prevention targets for nursing home IPC programs, including laboratory-confirmed *C. difficile*, urinary catheter use, catheter-associated urinary tract infections (CAUTIs), pneumococcal and influenza vaccination rates in residents, influenza vaccination rates in HCP, and antimicrobial stewardship.^48,113^ The CDC National Healthcare Safety Network (NHSN) is a web-based surveillance platform available to nursing homes that can support tracking of HHS priorities through modules for healthcare-associated UTI, laboratory-based identification for MDRO and *C. difficile* infection, and preventative process modules.^114^ NHSN provides nursing home-specific training, tool kits, and a web-based platform to help facilities identify HAIs in a standardized fashion.^115^ However, the IP needs dedicated time to understand and implement NHSN reporting processes and leadership support to maintain access to the NHSN system (e.g., serve as a NHSN user for the facility). During the COVID-19 pandemic, CMS required nursing homes to report COVID-19 data of residents and HCP and COVID-19 vaccination rates into NHSN.^116^

#### Standardized definitions and methods for surveillance

An effective surveillance program must have accurate, standardized, and reproducible methods to detect infections that minimize subjective interpretation and interobserver variability over time. The McGeer infection surveillance definitions for long-term care facilities, originally developed in 1991 and revised in 2012, provide a standard set of criteria designed for nursing home surveillance activities.^112,117^ Retrospective identification of infections is typically accomplished through the review of residents’ medical records, discharge summaries, laboratory and radiology reports, and medication and treatment records. Electronic access to health records is ideal. Each defined HAI should be recorded using standardized methods, such as line listings, and reported by the number of HAIs and rates of each HAI per 1000 resident-days.^112,118,119^

Although all CMS-certified nursing homes have been required to track a resident’s admission, and on a quarterly basis information about UTIs, pneumonias, *C. difficile*, and antibiotic-resistant bacteria using the Minimum Data Set, Minimum Data Set definitions are not the same as those used for HAI surveillance and should not be substituted for infection surveillance purposes.^30^

#### Implementation of IPC practices

Implementing evidence-based practices and recommendations can be challenging, particularly in nursing homes, which may have limited resources and HCP and residents who have high acuity of care. In this section, implementation strategies that have been studied and found to help implement IPC evidence to practice are described.

### Healthcare personnel (HCP) training, monitoring, auditing, and feedback

**14. What constitutes minimum IPC competency for nursing homes’ frontline (resident-facing) HCP?**

*Recommendation:* Nursing homes should:

1. Select training methods and content that addresses the diversity of the workforce and meets the needs of the HCP being trained
2. Provide job-specific, minimum IPC competency-based training, defined as the “minimum knowledge and skill needed to safely perform a task according to facility standards and policies”
3. Ensure dedicated time for HCP to receive regular, job-specific IPC education and to demonstrate competency
4. Document demonstrations of competency following IPC training
5. Evaluate competency before provision of care, specific procedures, introduction of new equipment or protocols, and on an as-needed basis to prepare for and respond to an infectious diseases event
6. Conduct competency assessments through direct observations by trained observers or online skills training that include:
  - Initial or core competency training conducted at-hire or during orientation
  - Ongoing competency training done annually or when new skills or knowledge are needed
  - Specialized competency training related to an area of specialization, such as wound care, central line dressing change, or tracheostomy care.

*Rationale:* Ongoing education and training to build a knowledge base is critical to prevent and manage infections, and studies have suggested that more attention to competency training is needed. In April 2008, the Institute of Medicine released “Retooling for an Aging America: Building the Health Care Workforce”^120^ recommending:

1. Requirements for HCP to demonstrate competence in basic geriatric care for obtaining and maintaining their licenses and certifications
2. Expansion of coursework and training for HCP in the treatment of older adults
3. Determination of training content for specialized or job-related competencies^121^

In a survey among nursing home HCP, 44% to 53% of respondents, depending on municipality, rated that their knowledge in “infection protection” was insufficient.^122^ Other studies have suggested that current competencies do not correspond with the tasks assigned to nursing home staff.^123^ Age and length of work experience had a positive, but not a strong, correlation with level of competence.^124,125^ In a national survey of nursing homes, the timing of IPC training (at new employee orientation and when an infection outbreak occurred versus other [only at new employee orientation, only when an infection outbreak occurred, or neither]) was associated with reduced indwelling urinary catheter use.^77^ Although evidence is sparse, McKinley and colleagues suggested that engaging environmental services and enhancing their knowledge and competencies are important to prevent infections and curtail outbreaks.^126^

Competency training is associated with better work satisfaction and lower turnover rates among nursing home staff.^122^ Minimum IPC competency for frontline HCP should include the knowledge, skills, and attitudes required to practice using reasoning, critical thinking, reflection, and analysis to inform assessment and decision-making in the prevention of infections and antimicrobial resistance.

Nursing homes should use objective tools to assess competency. For example, the Infection Control Assessment and Response Tools developed by CDC may be used to assess IPC practices and guide quality improvement activities.^127^ The CMS Civil Money Penalty Reinvestment Program developed the CMS Competency Assessment Toolkit to assess nursing home HCP competency, sufficiency, and performance. This competency assessment includes frontline assessments (behavioral, technical, and resident-based competencies), as well as a management assessment (behavioral and technical competencies).^128,129^

**15. How should nursing homes monitor IPC practices?**

*Recommendation:* Nursing homes should:

1. Monitor HCP adherence to IPC practices as part of implementing IPC policies
2. Assess the availability of supplies at the point of use to support IPC practices
3. Use findings from the annual IPC risk assessment and infection surveillance data to inform which IPC practices to audit. Commonly audited practices include but are not limited to:
  - Hand hygiene
  - Device insertion, maintenance, and removal
  - Cleaning and disinfection of environmental surfaces and reusable medical equipment
  - Use of PPE
  - Vaccination status of residents and HCP
4. HCP who perform practice audits should:
  - Receive training
  - Use standardized tools to support consistent monitoring.

*Rationale:* Nursing homes should use evidence-based tools developed by CDC and Agency for Healthcare Research and Quality (AHRQ) to monitor IPC practices^127,130^ including hand hygiene, reducing and improving device use, environmental and reusable medical device cleaning and disinfection, selection and use of appropriate PPE, and HCP and resident vaccination rates. Nursing homes may assess and verify performance of IPC practices by HCP through competency checks and observational audits (see Supplementary Material, Table 5). Competency checks should be done in a controlled environment, such as during scheduled training. Observational audits are performed while HCP are in the actual work environment. Observational audits provide compliance rates and identify process failures, such as overlooked high touch areas in the environmental cleaning process.

**16. What models are effective in implementing, auditing, and providing feedback on IPC policies and procedures?**

*Recommendation:* To effectively develop, disseminate, and implement IPC practices (including bundled practices and quality improvement interventions), nursing homes should:

1. Engage administrative and clinical HCP leadership (e.g., nursing and providers). If applicable, nursing homes should also include corporate leadership
2. Obtain input from HCP for strategies to implement IPC practices
3. Ensure HCP who are implementing practices have adequate time to receive education, appropriate training, and competency evaluation (see **14**)
4. Involve HCP in ongoing evaluation of practice implementation and opportunities for improvement
5. Audit and provide feedback on HCP adherence to recommended practices
6. Establish metrics to evaluate the impact of practice implementation and quality improvement.

*Rationale:* Changing practices in the nursing home is possible, but complex. To make changes, nursing homes should consider barriers and facilitators,^131^ local collaborators, ways to build on prior collaborative relationships, ongoing formative evaluation,^132^ and organizational culture and climate.^133^ Several studies have reported barriers to practice implementation as a result of HCP factors (e.g., turnover, high workload, attitudes) or organizational factors (e.g., funding, resources, logistics).^134^ For example, one study conducted in 36 acute and long-term care facilities described barriers to hand hygiene, such as inappropriate placement of sinks, traffic flow issues, inadequately stocked washrooms, HCP workload, and time constraints.^135,136^

To overcome these barriers, nursing homes should implement changes in a manner that fits within resident care workflow and that makes it easier for HCP to follow best practices. Nursing homes should give special focus to ensuring that needed supplies (e.g., ABHS, PPE, disinfectant wipes) are available to HCP at the point of care and that they have been educated on proper use. For example, when using EPA-registered cleaning and disinfectant products, HCP should follow the MIFU and instructions for safety.

Over the past two decades, numerous studies have shown that thoughtfully designed bundles (defined sets of evidence-based practices implemented together) can improve HCP practices and reduce infections, antimicrobial-resistant organisms, and outbreaks. One bundled IPC strategy that focused on specific IPC practices (e.g., gown and glove use during resident care) for targeted high-risk individuals with indwelling devices led to reductions in overall MDRO prevalence, new methicillin-resistant *Staphylococcus aureus* (MRSA) acquisitions, and CAUTI rates among those residents.^137^

To successfully implement IPC practices, whether individually or as part of a bundle, nursing homes should build interventions that have technical (content) and socio-adaptive components (leadership, communications).^137^ Leadership should be fully engaged in implementing bundles^138^ and HCP, including nurses and nurses’ aides, should be empowered to suggest strategies to reduce infections and the prevalence of antimicrobial-resistant organisms.^139^

One approach to implementation is using “Plan-Do-Study-Act” cycles,^133^ that is, providing step-wise processes for implementation of new practices and for evaluation of the effects of each change. In one study, interventions that targeted specific care tasks (e.g., oral care, physical restraints) were more likely to yield outcomes compared with interventions that required general practice changes (e.g., care philosophy). Human factors engineering, specifically the Systems Engineering Initiative for Patient Safety model, has been proposed as a method to identify new interventions^140^ through close observation of processes to develop mitigation strategies. For example, by involving nursing home HCP in proactive risk assessments and conducting direct observations of tracheostomy care, Katz and colleagues were able to identify high-risk steps in process and develop strategies to decrease the risk of infection transmission.^57^

Sustainable changes require strong leadership, organizational support, and teamwork.^133^ Successful strategies may include the use of educational materials,^141^ engagement in national and regional quality improvement collaboratives,^142^ and leveraging electronic medical records, where available, to integrate reminders to prompt actions (e.g., assessment and removal of indwelling catheters) or perform key IPC tasks. Defining metrics to evaluate changes in outcomes (e.g., CAUTI rates) or IPC processes (e.g., percent of HCP adherence to hand hygiene) is another important way to maintain awareness and support for implementation efforts.^133^

#### Commonly audited practices

**17. What should a nursing home’s hand hygiene program include?**

*Recommendation:* A nursing home’s hand hygiene program should include:

1. Interactive, regular education with demonstrations of technique, auditing, feedback, and access to educational materials
2. Active engagement by the nursing home’s leadership, clinical HCP, and nonclinical HCP in the practice and promotion of hand hygiene
3. Easy access to ABHS (see **23**).

*Rationale:* Proper hand hygiene is the basic tenet of IPC. However, hand hygiene adherence remains a challenge. In one study, hand hygiene adherence occurred in 96 of 352 observations before resident care (27.3%) and only 165 of 358 observations after resident care (46.1%).^143^ Some of the factors influencing adherence include HCP time constraints, availability of hand sanitizers at point of care, and competing priorities.

Several studies have shown that hand hygiene programs are effective, efficient, and reduce infections as well as antimicrobial-resistant organisms.^144,145^ Ensuring HCP understand how to incorporate hand hygiene into resident care activities based on type of care delivered and length of time spent with a resident can reinforce the importance of adherence. Hand hygiene programs that involved distribution of pocket-sized hand sanitizers and educational materials were effective in enhancing hand hygiene and reducing infection rates.^146–148^ Tailoring hand hygiene products to local preferences can enhance engagement and improve compliance with key hand hygiene measures.^135,136^ Studies have shown that multicomponent interventions that include performance feedback after the first evaluation phase, several training sessions, assessment of hand hygiene technique, a full day of hand hygiene training, and focusing on institutional communication can improve hand hygiene rates. Furthermore, education and awareness of institutional policies can enhance hand hygiene, particularly in high-risk activities.^149^ HCP engagement is important in deciding the best two or three interventions to improve hand hygiene compliance.^150^ Informed by local challenges and cultures, a combination of these strategies should be used to improve hand hygiene.

Auditing HCP adherence to hand hygiene during resident care activities may be conducted in various ways.^135,136^ Nursing home HCP can be trained to serve as secret observers on units as part of their routine activities. Residents and visitors might volunteer to observe HCP hand hygiene practices.^151^ Some healthcare facilities have incorporated use of automated or electronic hand hygiene monitoring systems into units to capture more data than observations might be able to generate.^152,153^

**18. How should nursing homes assess HCP knowledge and skill in device insertion, maintenance, and removal?**

*Recommendation:*

1. HCP should be knowledgeable about medical devices (e.g., central lines, indwelling urinary catheters, percutaneous gastrostomy tubes, tracheostomy tubes) including:
  - The risks associated with their use
  - Recommended IPC practices during placement, maintenance, and removal.
2. Nursing homes should document:
  - The presence, indication for, and duration of a medical device
  - Regular assessment of the ongoing need for a device, presence of signs or symptoms of infection or device malfunction, and opportunities for early and prompt removal
  - Adherence to the recommended steps during insertion, maintenance, and removal of the medical device
3. Nursing homes may conduct audits using standardized forms or forms tailored to the needs and processes of the nursing home.

Rationale: A bundled approach to implementing safe device use and maintenance has been shown to successfully reduce device-related infections like CAUTI and CLABSI in nursing homes. In a cluster-randomized clinical trial at 12 community-based nursing facilities focused on high-risk residents with indwelling urinary catheters, a multimodal targeted infection program reduced the prevalence of MDROs by 23% and the incidence of device-related infections by 45%. The interventions included preemptive barrier precautions during care of residents with indwelling devices, active surveillance for MDROs and infections, and HCP education. In addition, the multicomponent intervention improved IPC knowledge in HCP and was expected to benefit payors by reducing costs and improving health outcomes.^137^ Similarly, a multimodal approach successfully reduced adverse events and infections in other types of devices, such as gastric tubes and tracheostomies.^154^

In a study that evaluated the effect of implementing IPC bundled practices focused on medical device care and handling, using a multidisciplinary, collaborative approach, CAUTIs were reduced by 50% and CLABSIs by 25%.^155^ The program focused device insertion technique, reviewing the indication and ongoing need of the device, and audited specific practices for each device. Among residents who have central lines, the bundle assessment included changing the dressing, tubing, and needleless connectors; verifying that dressings are clean, dry, and intact; and scrubbing the hub before accessing the line.^156^ In residents with indwelling urinary catheters, the bundle assessments included addressing functioning (e.g., unobstructed flow) and handling during care (e.g., collection bag below the bladder, proper technique when accessing for specimen collection).^157^ Tools and checklists that can support the implementation and assessment of device-associated prevention bundles are available.^158,159^

**19. How should nursing homes conduct environmental cleaning and disinfection?**

*Recommendation:* Nursing homes should:

1. Have clearly written policies on the processes and time involved in cleaning and disinfection of environmental surfaces in shared areas, residents’ rooms, and for reusable medical equipment
2. Ensure that written policies address the frequency of both routine cleaning and disinfection practices, and cleaning and disinfection practices during outbreak situations (see **30**)
3. Audit practices for equipment and areas that are cleaned and disinfected, such as frequency and adequacy of cleaning and adherence to contact time (how long a disinfectant remains wet on a surface)
4. Assess availability of appropriate cleaning and disinfection supplies at the point of care, ensuring that products are EPA-registered as effective for the purpose for which they are being used (see **31**)
5. Use objective methods for evaluation of routine environmental cleaning, which may include direct observation, fluorescent markers, or adenosine triphosphate (ATP) bioluminescence
6. Focus on HCP education and training and provide regular performance feedback.

*Rationale:* Environmental contamination is a risk factor for transmission of pathogens. Multiple studies have demonstrated the presence of clinically significant pathogens on items and high-touch surfaces in residents’ rooms and common areas.^160–165^ One study used a nonpathogenic deoxyribonucleic acid (DNA) marker inoculation onto the television remote controls in rooms of two ambulatory long-term care facility residents and demonstrated dissemination of the DNA marker to the hands of nursing home residents, high-touch surfaces in the room, on the ward, and to shared portable equipment, indicating the importance of cleaning of surfaces frequently touched by residents.^161–163,165,166^ Studies showed that environmental contamination often depends on frequency and time spent in the cleaning of individual resident rooms and common areas, as well as overall prevalence of organisms in nursing facilities. Proper cleaning and disinfection have been demonstrated to reduce microbial burden.^161,163^

Audits of reusable medical device use and reprocessing have also revealed issues in IPC. One study found that HCP in two skilled nursing facilities were incorrectly using blood glucose monitoring equipment, leading to six hepatitis B cases.^167^ These outbreaks highlight the need to use audits specifically to address adherence to evidence-based IPC practices by HCP.^168^ Facilities may also evaluate whether there are technological advances (e.g., single-use glucose monitoring devices) that could reduce transmission risk.^160^

Cleaning supplies should be readily available and easily accessible. Facilities should use disinfectants approved for use against the pathogen of interest. The most commonly used method for assessing the adequacy of environmental cleaning in nursing homes is visual assessment performed by the supervisor for randomly selected rooms.^169^ This method to evaluate cleanliness may identify visible soilage but has poor correlation with identification of microbial contamination and is subject to high potential for observer bias.^170^ A study comparing use of visual inspection, performance observation, and ATP interpretation found the results to be inconsistent, with weak positive correlation between visual inspection and performance observation but no correlation between visual inspection and ATP interpretation.^171^ CMS has recommended that nursing homes use objective methods for evaluation of routine environmental cleaning, including direct practice observation, fluorescent markers, or ATP bioluminescence.^172^

Studies evaluating the effectiveness of different methods for assessing adequacy of environmental cleaning have predominantly been in acute-care settings.^170,171,173^ The results of these studies support combining routine objective assessments with HCP education and performance feedback for improvement in environmental cleaning.^170^ However, currently inadequate evidence exists to make a preferential recommendation for the best objective assessment methods to use in the nursing home setting. Rather, nursing homes should choose among objective measures that include regular HCP education and performance feedback for their environmental cleaning and disinfection program, based on available resources.

**20. How should nursing homes ensure proper use of PPE?**

*Recommendation:* Nursing homes should:

1. Develop a written policy for Standard and Transmission-Based Precautions that describes the types of PPE, indications for use, and proper steps for donning (putting on) and doffing (removing) the PPE
2. Provide HCP with ready access to PPE, including gowns, gloves, eye protection, surgical masks, and respirators
3. Ensure HCP select and use PPE based on the nature of the resident interaction and the potential for exposure to blood, body fluids, or infectious material
4. Monitor adherence to practices and provide feedback (see **22**)
5. Make PPE available at the point-of-care when residents are placed on Transmission-Based Precautions.

*Rationale:* Standard Precautions are the basic practices that apply to all individuals in settings where healthcare is delivered, regardless of a person’s suspected or confirmed infectious state.^174^ Standard Precautions protect HCP and prevent HCP or the environment from transmitting pathogens to others. Appropriate selection and use of PPE are essential elements of Standard Precautions and based on the type of interaction and exposure anticipated when providing care. Examples of PPE selection as part of Standard Precaution include wearing gloves when contact with blood or other body fluids, mucous membranes, or non-intact skin could occur; wearing gowns during procedures and activities that can lead to splashes of blood, body fluids, or other secretions; and using protective eyewear and mask or face shield to protect eyes, nose, and mouth during procedures and activities that could generate splashes of blood, body fluids, and secretions.

Transmission-Based Precautions are applied when Standard Precautions alone may not be sufficient to prevent pathogen transmission. The type of Transmission-Based Precautions used are based on the known or suspected routes by which an infectious pathogen can spread. For example, Contact Precautions, which includes use of gowns and gloves during care interactions, are used to disrupt the spread of pathogens by direct or indirect contact with a resident or their environment. When used either singly or in combination, they are always used in addition to Standard Precautions.

Although the use of gowns and gloves has been shown to reduce transmission of pathogens,^175^ their use in nursing homes, especially gowns, remains suboptimal. When nursing homes have PPE readily available, use by HCP and adherence to precautions are increased. Gowns stored in supply closets, for example, are rarely used. Along with interactive education and leadership buy-in, making PPE available at the point of care increases adherence and reduces colonization pressure and infections among high-risk residents.^137,176^

**21. What should nursing homes’ vaccination policies include?**

*Recommendation:* Nursing homes should have written, up-to-date policies for vaccination of residents and HCP that include education, training, and monitoring of vaccination acceptance rates.

*Rationale:* A vital component of the IPC program is promoting vaccination to residents and HCP against respiratory viruses and other vaccine-preventable infections to prevent introduction and spread of these pathogens in nursing homes. Facility policies and procedures should address when vaccines are offered, how to promote vaccine acceptance, how to transport and store the vaccines properly, how to administer them safely, and how to maintain documentation of resident and staff vaccination status. Strategies to increase staff vaccination acceptance, including promoting the benefits of vaccine, providing educational materials, and having conversations to address questions and misconceptions, (see **12**), are also important when discussing vaccines with HCP, residents, and families.^177^

Evidence-based tools, such as those developed by AHRQ, can support nursing home efforts to monitor vaccination acceptance rates of residents and HCP, coordinate next doses, and identify vaccine hesitancy.^178^ These resources enable nursing homes to track and trend vaccine administration data (e.g., COVID-19, influenza) for both residents and HCP and assist with compiling accurate data for reporting to the CDC NHSN.^59^

**22. What methods should nursing homes use to provide feedback on HCP adherence to IPC practices?**

*Recommendation:*

1. Nursing homes should use audit and feedback methods to improve and sustain compliance with evidence-based practices:
  - For individual HCP, nursing homes should use specific, just-in-time feedback on the process being audited
  - At the unit or facility-level, nursing homes should use simplified, aggregated data from audits during rounds, QAA meetings, personnel newsletters, and reporting huddles.
2. Nursing homes should train HCP who conduct these audits to use standardized tools and definitions.
3. Nursing homes should provide feedback to HCP on signs of potential lapses in IPC practices during care.

*Rationale*: Sustainable improvements in IPC require culture change, increased HCP involvement, explicit administrative support, and meaningful, timely feedback. Providing feedback to facility HCP about their adherence to IPC practices helps reinforce the importance of these activities and maintains awareness of the facility’s policies and procedures. Feedback also serves as a strategy to engage HCP and develop shared goals to reduce infections in nursing homes.

Nursing homes may share feedback in multiple ways. For example, performance monitoring data can be aggregated and summarized facility-wide or broken down by unit, shift, or provider type to compare differences in performance. Individualized feedback can be shared during one-on-one meetings with HCP or provided at the moment when the care activity is occurring or “just-in-time,” to provide a gentle reminder and immediate learning opportunity. Multimodal interventions that incorporate feedback of hand hygiene compliance with optional interventions have been successful (e.g., e-learning, kick-off workshop, observer training, team training).^179^ In one study, investigators created a tool to measure the IPC practices such as hand hygiene compliance, environmental contamination, prevalence of MDROs using active surveillance approach, appropriate use of indwelling devices, and antibiotics. This led to the creation of a personalized facility-level spider plot that was then shared with respective facilities and led to standardization of implementation across sites, enhanced transparency, and improved outcomes.^180^

### Environment of care

**23. Where should nursing homes place ABHS dispensers?**

*Recommendation:* Nursing homes should:

1. Place ABHS dispensers where they are easily accessible at a room’s entry and at the point of care
2. Install ABHS dispensers in accordance with local fire regulations
3. Have a hand hygiene program that includes:
  - Widespread availability of hand hygiene products throughout the facility for use by HCP, visitors, and residents
  - Engagement of HCP in selection and feedback on products.

*Rationale:* ABHS products should be provided at the entry to the room and at the point of care.^181^ Hand hygiene products should be tailored to local preferences to increase HCP engagement and adherence.^135,136^

In addition, ABHS products and dispensers should be selected with considerations for nursing home residents’ needs. Although concern that cognitively impaired residents may be injured by ingestion of ABHS is cited as a barrier to installation of ABHS dispensers in hallways and rooms, instances of ingestion are exceedingly rare. The benefits of increasing ABHS access to support hand hygiene outweigh potential risks. The dispensers should be installed such that they minimize any accidents or spills. In secure units, alcohol-based hand sanitizers that remain under the control of the healthcare worker, such as an individual pocket-sized bottle, or specially designed anti-ligature wall-mounted dispensers are strategies that make product available to HCP who care for and interact with residents with cognitive or behavioral impairment. Questions on alcohol-impregnated wipes being efficacious in eliminating pathogens remain as ABHS products need further study; however, alcohol-impregnated wipes may be a convenient option for nursing home residents who are unable to get easily to sinks or wall-mounted dispensers.^182^

**24. How should nursing homes handle laundry and linens for IPC?**

*Recommendation:* Nursing homes should:

1. Use industrial laundry equipment (onsite or offsite) to process laundry and linens. Exceptions may be made for clothing that is laundered by the resident; however, laundry machines used onsite for clothing that is laundered by the resident or family should be disinfected and maintained in accordance with the laundry machines’ MIFUs
2. Store clean laundry and linens in a location that protects them from environmental contamination
3. Educate HCP on safe practices, including PPE selection, when handling and/or changing used linens (e.g., gowns and gloves when handling linens that are grossly soiled)
4. Require HCP to wear gowns and gloves when changing bed linens of residents who are on Enhanced Barrier or Contact Precautions to prevent contamination of clothing and subsequent transmission to other residents.

*Rationale*: Current industrial laundry processes clean laundry and linens via physical, chemical, and thermal actions, all of which result in hygienically clean items.^183^ Outbreaks associated with laundered healthcare textiles are rare, including an inadvertent exposure of clean textiles to environmental contamination (e.g., Aspergillus, Rhizopus) or a process failure during laundering (e.g., *Bacillus cereus*).^183^ Occupationally acquired infections (e.g., scabies, hepatitis A) have been tied to mishandling of soiled linens and failure by HCP to use PPE properly.^183^ Studies in which gowns and gloves of HCP are cultured after specific care activities have shown that handling linens frequently results in contamination (25% of interactions involved changing linen).^184^ In a small nonrandomized clinical trial, the use of Enhanced Barrier Precautions decreased *S. aureus* transmission.^175^ Enhanced Barrier Precautions classify changing of linens as a high contact activity.^176^ Thus, HCP should be educated in safe practices when handling and/or changing used linens (e.g., do not shake or sort, do not carry against the body). Use of precautions lowers the risk of contamination and transmission.

### Outbreak preparedness and response

**25. What strategies should nursing homes use to detect and respond to outbreaks?**

*Recommendation:* Nursing homes should:

1. Be aware of viruses and other pathogens circulating in the community
2. Understand how pathogens that typically cause outbreaks enter and spread within a facility and how to implement pathogen-specific symptom screening
3. Educate HCP to identify and report when they or residents have symptoms that could be consistent with a highly transmissible pathogen
4. Implement sick leave policies and processes for HCP that promote timely reporting of illness and appropriate action (see **11**)
5. Implement early (point of care) diagnostic testing to identify pathogens
6. Implement appropriate Transmission-Based Precautions based on symptoms, while awaiting a resident’s diagnosis
7. Communicate with referral hospitals and public health departments
8. Vaccinate residents and HCP
9. Identify approaches for the facility’s access to and use of early therapeutics.

*Rationale*: Outbreaks are common in nursing homes because close quarters and shared caregivers exacerbate asymptomatic and presymptomatic transmission among this vulnerable population. Over the past decade, outbreaks due to viruses (COVID-19,^185,186^ influenza, RSV,^187^ and norovirus^188^), bacteria (*S. pyogenes*,^189^ *S. pneumoniae*,^190^ Group A *Streptococcus,* ^186^ *A. baumannii*), fungi (*C. auris*),^22^ and parasites have been reported in nursing homes. Many of the outbreaks were regional and detected in hospitals, demonstrating that nursing homes contribute to the spread of MDROs, such as carbapenem-resistant Enterobacterales and *Acinetobacter*.^191–193^

Clear and consistent policies for HCP, residents, and visitors that support reporting of signs and symptoms of illness help nursing homes prevent outbreaks, reduce absences, and improve reporting. Such policies should include the following: criteria for initiation of Transmission-Based Precautions, communication to visitors to postpone non-urgent in-person visitation if they are ill, and development of programs to provide guidance to staff about whether they should be excluded from work as a result of an infectious illness. This approach can lead to reductions in bed closure days, fewer delays in initiation of Transmission-Based Precautions, better reporting, and fewer isolation days without a substantial increase in missing work. Nursing homes should focus on changing processes but with clarification and backed by evidence. In addition, tracking influenza, COVID-19, and other novel illnesses as they arise and providing access to rapid testing and treatment are critical strategies to prevent ongoing transmission and severe outcomes.^194^

Nursing homes should train and educate HCP to notify the IPC program of suspected cases of gastrointestinal, respiratory, and skin and soft tissue infections in residents. The IPC program’s HCP should work with local resources (i.e., public health departments) to diagnose new cases and respond appropriately.^22,195–197^

**26. How should nursing homes use point-of-care testing to detect and control the spread of respiratory pathogens?**

*Recommendation:* Nursing homes should:

1. Have the capacity to perform point-of-care testing for early detection of viral respiratory pathogens to prevent them from being introduced into the nursing home when community transmission is present and to enable the early treatment of residents.
2. Liaise with laboratory or infectious diseases consultants regarding selection of point-of-care tests.

*Rationale:* Point-of-care testing reduces the impact of viral respiratory infections and helps to prevent and manage outbreaks in nursing homes. Point-of-care testing can help facilities isolate and treat people with certain viral respiratory infections who may be infectious, even before onset of symptoms, and help stop outbreaks. During an RSV outbreak in an adult nursing home, rapid test results along with intensified IPC measures were instrumental to halting the spread and ending the outbreak.^187^ Furthermore, most antiviral medications (e.g., oseltamivir, nirmatrelvir/ritonavir) are more effective when given early in infection.

Before the COVID-19 pandemic, nursing homes generally did not have capacity to implement point-of-care testing for respiratory viruses for residents and HCP. The primary rapid testing performed in most facilities was for influenza and was used only for residents. During the COVID-19 pandemic, point-of-care tests—mainly using lateral flow devices—were deployed extensively and used for testing of both residents and HCP.^185^

Antigen point-of-care tests for COVID-19, influenza, and RSV are less sensitive than nucleic acid amplification tests (e.g., polymerase chain reaction) and can return false negative results. Residents who are symptomatic or known to be exposed to the virus should be presumed to be infected and remain on appropriate Transmission-Based Precautions until the negative test result is confirmed by PCR or repeated antigen testing.

**27. How should nursing homes implement respiratory hygiene, cough etiquette, and source control to control the spread of respiratory pathogens?**

*Recommendation:* Nursing homes should have policies and protocols for respiratory hygiene, cough etiquette, and masking for source control to prevent transmission of infection to HCP, visitors, and residents that include:

1. Education of HCP, residents, and visitors in how to prevent transmission of respiratory pathogens
2. Signage and reminders at entrances and in shared areas on hand hygiene and how and when to wear a mask for source control
3. Appropriate and easily accessed supplies (e.g., masks, ABHS) so practices can be followed.

*Rationale:* Clear and easily visible policies, guidance, and reminders about recommended practices to prevent transmission of respiratory pathogens should be posted at facility entrances, break rooms, and shared areas frequented by residents. This is particularly true when changes in facility practice are made in response to a facility outbreak or increases in respiratory virus transmission in the community. For example, when there is an increase in respiratory virus transmission in the community, CDC recommends that healthcare facilities consider having everyone mask upon entry to the facility to ensure better adherence to respiratory hygiene and cough etiquette for those who might be infectious.^8^ Providing necessary supplies will ensure that individuals are able to follow recommended practices. CDC has created a viral respiratory pathogen toolkit for nursing homes to help them prepare for and prevent the spread of respiratory viruses.^198^

**28. How should nursing homes use ventilation to control the spread of respiratory pathogens?**

*Recommendation:* Nursing homes should:

1. Ensure they are compliant with building code requirements for heating, ventilation, and air conditioning
2. Monitor ventilation systems in accordance with engineers’ and manufacturers’ recommendations to ensure optimal performance
3. Ensure the IPC program collaborates with facility management, particularly in circumstances when considering implementation of any supplemental strategies to enhance ventilation (e.g., during a facility respiratory pathogen outbreak) (see **5**)
4. Have a process in place for isolating residents with pathogens for which an airborne infection isolation room (AIIR) is recommended (e.g., tuberculosis)
  - If an AIIR is not available, the residents should be transferred as soon as is feasible to a facility where an AIIR is available. Place a mask on the resident (if tolerated) and isolate the resident in a private room with the door closed, while awaiting transfer.

*Rationale*: Good ventilation is important to maintain air quality and reduce risk for transmission of pathogens that spread through the air. Facilities should ensure that they are compliant with building code requirements for heating, ventilation, and air conditioning; regular maintenance is important to ensure optimal performance of these systems. The American Society of Heating Refrigeration and Air-Conditioning Engineers provides guidance about air changes per hour based on the type of room or common area and the recommended minimum efficiency reporting value filtration level for optimal air flow and air quality.^70^ The IPC program should provide input to facility management for ventilation and have awareness of the general pattern of airflow throughout the building and the location of return registers and systems that exhaust to the outdoors. The IPC program should have awareness of rates of respiratory virus transmission in the facility and occurrence of outbreaks that could inform the use of supplemental strategies to improve ventilation and enhance filtration and purification of air.

Nursing homes should also have a plan for management of residents suspected or confirmed to be infected with a pathogen (such as measles, tuberculosis, or a novel pathogen) for which an AIIR is recommended. In most circumstances, the affected resident should be transferred to a facility with such capacity since most nursing homes do not have AIIRs. In such situations, local public health authorities and external partners, such as referral hospitals, should be notified immediately.

### Strategies for specific IPC practices in nursing homes

#### Environmental cleaning and disinfection

**30. How frequently should nursing homes clean and disinfect surfaces in residents’ rooms, shared bathrooms, and shared areas?**

*Recommendation:* Although the optimal frequency of cleaning and disinfection of areas in nursing homes remains unclear, nursing homes should provide adequate time for HCP to:

1. Routinely clean and disinfect resident rooms, shared bathrooms, and shared areas at least once a day, paying particular attention to high-touch surfaces with an EPA-registered disinfectant active against the pathogens most likely to contaminate the resident care environment
2. Perform cleaning immediately upon noticing visibly soiled surfaces
3. Increase the frequency of cleaning and disinfection during outbreaks.

*Rationale:* Cleaning and disinfection of resident rooms and shared areas are important in preventing transmission of pathogens within the nursing home. Limited studies have evaluated the optimal frequency of cleaning and disinfection of residents’ rooms and shared areas. CMS guidance calls for routine cleaning and disinfection of frequently touched or visibly soiled surfaces in common areas, residents’ rooms, and at time of discharge.^18^ One prospective study of environmental contamination and cleaning quality found that, in addition to the high MRSA burden within the facility, spending less time during cleaning and/or less frequent cleaning of common rooms were significantly associated with MRSA environmental contamination.^164^ The frequency of cleaning ranged from 1 to 3 times/day (median 2.5 time) within the 10 nursing homes that participated in that study.

Nursing homes should consider cleaning and disinfecting resident rooms, shared bathrooms, and shared areas at least once a day, especially focusing on high-touch surfaces. Because an increased microbial burden on environmental surfaces has been shown during outbreaks,^199,200^ facilities should increase the frequency of cleaning and disinfection during outbreak response.

**31. What are the cleaning and disinfection considerations for equipment shared among residents (e.g., shower chairs, blood pressure cuffs, mechanical lifts) and residents’ personal belongings?**

*Recommendation:* Nursing homes should:

1. At least daily, clean and disinfect residents’ frequently used items (e.g., canes, walkers, remotes, tablets, phones)
2. After each use, clean and disinfect items that are shared among residents
3. Choose products that are EPA-registered for the specific cleaning and disinfection purpose
4. Follow the MIFU for equipment and the cleaning products used to avoid damaging existing equipment and objects

The laundry section (see **24**) addresses cleaning of residents’ soft items, as appropriate. Care of residents’ personal hygiene items (e.g., toothbrushes) are outside the scope of this document.

*Rationale:* CMS guidance outlines the requirement for routine cleaning and disinfection of resident care equipment, including equipment shared among residents (e.g., blood pressure cuffs, rehabilitation therapy equipment, blood glucose meters).^18^ To avoid transmission of pathogens among residents, nursing homes should clean and disinfect all items that are shared among residents after each use.

Studies have not evaluated the optimal frequency of cleaning residents’ personal belongings, but, for residents’ frequently used items (e.g., canes, walkers, remotes, tablets, phones) the same principles should apply as described above for high-touch surfaces in resident’s rooms (see **30**). Nursing homes should clean, or when feasible, facilitate cleaning with residents or family members, frequently used personal items of residents at least once daily.

CDC guidelines for environmental IPC in healthcare facilities recommend following MIFUs for cleaning and disinfection of medical equipment.^201^ When MIFUs are not available, noncritical medical equipment (e.g., stethoscopes, blood pressure cuffs, equipment knobs and controls) usually only require cleaning and low- to intermediate-level disinfection depending on the nature and degree of contamination.

To facilitate timely cleaning and disinfection of these items, the cleaning and disinfection products should be available to HCP at the point of use. When choosing cleaning and disinfection products, nursing homes should use products that are EPA-registered for the specific cleaning and disinfection purpose.^202^ HCP and should follow the MIFU for selected cleaning products for instructions about material compatibility, dilution, contact time, storage, shelf-life, safe use, and disposal.

### Resident placement and PPE use

**32. How should Transmission-Based Precautions for residents who are chronically colonized with MDROs apply to dining (in-room and group), rehabilitation and therapy (in-room and group), recreational, and other high-contact activities, as well as interactions with residents’ visitors, including students, trainees, and volunteers?**

*Recommendation:* Nursing homes should:

1. Not restrict residents who are chronically colonized with MDROs from visitation, social activities, dining, rehabilitation and therapy, or recreational activities
2. Apply Enhanced Barrier Precautions for residents infected or colonized with MDROs targeted by CDC. Nursing homes may consider, based on their policies, applying Enhanced Barrier Precautions more broadly to include other epidemiologically important MDROs
3. Not require that visitor(s) seeing a single resident who is chronically colonized with an MDRO wear specific PPE, although nursing homes may offer PPE for high-contact care or for care, in which Standard Precautions would require PPE.

*Rationale:* Nursing home residents often are colonized chronically with MDROs.^6,7^ Colonization can persist for prolonged periods of time. The strategies used to prevent MDRO transmission in acute care hospitals where length of stay is often short, such as Contact Precautions, which include room restriction, are challenging to implement in nursing homes. In particular, extended isolation and room restriction can have adverse effects on a resident’s quality of life, since many social and rehabilitative activities occur in a group setting. Enhanced Barrier Precautions are a less restrictive approach that does not require isolation of the resident or restriction from social activities.^176^

### Enhanced barrier precautions

CDC promotes Enhanced Barrier Precautions to reduce transmission during care of residents who are infected or colonized with novel or targeted MDROs and as a strategy to address the risk of MDRO acquisition associated with indwelling devices and wounds. Enhanced Barrier Precautions expand the use of PPE beyond situations in which exposure to blood and body fluids is anticipated (i.e., Standard Precautions) and refer to the use of gowns and gloves during high-contact resident care activities that provide opportunities for transfer of MDROs to hands or clothing of HCP.

Use of Enhanced Barrier Precautions is recommended for all residents infected or colonized with a novel or targeted MDRO when Contact Precautions do not otherwise apply. Enhanced Barrier Precautions may also be considered during high-contact care of residents with wounds and/or indwelling medical devices (e.g., central line, urinary catheter, feeding tube, tracheostomy/ventilator) regardless of MDRO colonization status.

Enhanced Barrier Precautions are intended to disrupt the spread of pathogens without restricting residents’ movements or participation in the facility. However, situations may occur when public health will recommend time-limited room restriction for residents colonized with targeted MDROs (e.g., during an outbreak). Facilities may consider applying Enhanced Barrier Precautions to residents who are infected or colonized with other epidemiologically important MDROs based on facility policy.

Unlike HCP, visitors in nursing homes will generally only interact with the individual resident who they are visiting. During those one-on-one interactions, gowns and gloves are generally not recommended except in situations where Standard Precautions apply (e.g., if the activity was going to result in the visitor having contact with blood or body fluids).^203^

**33. How should Transmission-Based Precautions apply to residents unable to tolerate IPC interventions (such as room restriction) implemented as part of outbreak response (e.g., individuals with significant cognitive impairment)?**

*Recommendation*: For residents who are unable to tolerate IPC interventions implemented as part of outbreak response, nursing homes should:

1. Emphasize prevention measures that do not depend on room restriction (e.g., vaccination, therapeutics) to prevent spread among all residents
2. Have HCP routinely assist residents in performing hand hygiene
3. During outbreaks, utilize horizontal IPC approaches, which are intended to control the spread of multiple organisms simultaneously.

*Rationale:* Residents with cognitive impairment may be particularly affected by room restrictions, limitations on group activities, or other IPC practices that are typically implemented to control facility outbreaks. For example, residents may have difficulty connecting with new caregivers or, when isolated, lack social cues to eat during mealtimes.^204^ Therefore, additional supervision (e.g., support with personal hygiene) and staffing to care for residents with cognitive impairments are often necessary to support the use of prevention strategies during outbreaks; use of familiar staff who have established relationships with these residents should be prioritized, when feasible.^205^

Despite some lessons learned during the COVID-19 pandemic, strong evidence remains scarce on how to best address these challenges. During outbreaks, when feasible, the nursing home should focus on interventions that do not require residents’ participation. For example, vaccination can limit the impact of influenza outbreaks.^206^ The value of horizontal IPC practices (e.g., increasing frequency of environmental cleaning and disinfection or hand hygiene) is that they can reduce transmission risk across more than one pathogen. For example, during respiratory virus season, universal use of masks for source control by HCP during a respiratory outbreak could limit spread to residents even before a specific virus has been identified.^8^ Although the use of masks by caregivers can result in residents having difficulty recognizing and connecting with them, creative strategies such as maintaining consistent assignment between residents and caregivers or using large photos and name tags to help with identification could help overcome those challenges.

**34. How should nursing homes educate residents, families, and visitors on appropriate IPC practices?**

*Recommendation:* Nursing homes should:

1. Educate and engage residents, families, and visitors in adoption of appropriate practices for hand hygiene, respiratory hygiene, PPE, antibiotic use, vaccination, and practices for the prevention and control of emerging infections and outbreaks
2. Post IPC policies and reminders in nursing home reception areas to reinforce interactions with HCP. Nursing homes may consider including signage at the entrance to the building, at reception, in family newsletters, on digital information screens, and on resident-used tablets and computers.

*Rationale:* Limited data exist with regard to how nursing homes should educate residents, families, and visitors in appropriate practices for hand hygiene, respiratory hygiene, PPE, antibiotics, vaccination, and emerging infections and outbreaks. Evidence has demonstrated that engagement leads to improved practices and better acceptance. O’Donnell and colleagues found that involving residents was important in designing interventions to improve residents’ hand hygiene, as ease of use of alcohol-based hand hygiene products improved implementation.^207^ No studies were found about educating residents, families, and visitors on respiratory hygiene or vaccination. However, ensuring that staff conducting educational in-services for residents and families are confident in vaccine effectiveness could improve acceptance. One qualitative study on PPE use showed that, although HCP expressed concerns about residents’ perceptions of gown use, residents were shown to be pragmatic if educated.^208^ HCP may discuss antibiotic use during advanced care discussions, acknowledging that care discussions for infection treatment decisions most frequently occurred at the time of medical crisis or after the medical events (e.g., aspiration).^209^ Advanced care planning is influenced by state policies. States with more mature policies were more likely to address antibiotic use during advanced planning meetings.^34^

**35. How should nursing homes assess whether to adopt evolving IPC practices to complement current IPC efforts?**

No recommendation.

Adoption of supplemental practices may be considered in response to a problem, such as high infection rates, outbreaks, or a survey citation that occurs despite careful adherence to all recommended core IPC practices. Nursing home leadership, in conjunction with the QAA committee, should select supplemental practices based on review of high-quality evidence, ideally provided by randomized controlled clinical trials.

Supplemental practices may be adopted in the short term until a problem is resolved, or they may be formally adopted as part of routine nursing home IPC policies and procedures, as an integral part of the collective journey toward high-quality and high-reliability nursing home care. The QAA committee should vet such QAPI projects to evaluate whether care is improved using metrics that assess value over time. Investments in innovative IPC practices may provide evidence toward potentially new standards; however, such investment requires champions, efforts by personnel, and collection of data to determine the associated benefit.

As one example, interest has increased in decolonization as a strategy to reduce both MDRO prevalence and transmission, as well as infections and hospitalizations among nursing home residents. Decolonization refers to the use of antiseptic soaps and nasal products to reduce the burden of bacteria and other pathogens on the skin and in the nose. Studies have shown that routine chlorhexidine bathing in nursing homes and long-term acute care hospitals can reduce MDRO prevalence, environmental contamination, and bloodstream infection.^210–213^ When applied in specific ways, chlorhexidine is active against Gram-positive and Gram-negative MDROs and *Candida auris*.^214^ Nasal decolonization also has been used to successfully reduce MRSA in nursing homes.^215^

Recently, a 28-nursing-home randomized clinical trial showed that the combination of chlorhexidine for routine bathing plus nasal iodophor decolonization twice daily every other week for all residents prevented nearly two infection-related hospitalizations per month per 100-bed nursing home and reduced MDRO prevalence by 30%.^216^ Nursing homes should consider these and other evolving and innovative practices when developing campaigns to address concerns about infections, hospitalizations, and MDROs.

Supplementary Material, Table 6 provides several examples of supplemental activities in nursing home settings that were evaluated by large randomized clinical trials and have begun to answer persistent IPC questions. Notably, some of the approaches in these trials might be helpful when performed correctly, whereas other commonly used approaches (e.g., cranberry capsules) have not shown benefit.

Additional examples of large-scale randomized clinical trials in nursing homes have included video-enhanced advanced care planning and the comparative effectiveness of various types of influenza vaccines.^217,218,239^

### Stewardship

Clinicians frequently use diagnostic tests when residents are suspected to have illness due to infection. Diagnostic stewardship is the term for efforts aimed at improving clinicians’ testing choices and interpretation of test results to improve accuracy and reduce the risk of unnecessary testing and subsequent treatment.^219^

Antimicrobial stewardship is the term used for efforts to mitigate the development of antimicrobial resistance and improve outcomes by selecting appropriate antimicrobial treatments and durations for treatment. Because test results strongly influence clinicians’ antimicrobial prescribing practices, diagnostic and antimicrobial stewardship efforts work together for the common goals of improved resident outcomes and mitigation of antimicrobial resistance.^220^

### Diagnostic stewardship

**36. What is the role of the laboratory in supporting diagnostic stewardship in nursing homes?**

*Recommendation:* In partnering with microbiology laboratories, nursing homes should incorporate the principles of diagnostic stewardship rules in the ordering, interpreting, and reporting.

*Rationale:* Current evidence suggests that antimicrobial stewardship interventions in nursing homes may be improved by focusing on interventions that target phases of the diagnostic process. Engaging the medical director, nursing home leadership, and HCP in diagnostic stewardship interventions can improve antimicrobial use in the long term.

Nonspecific symptoms, such as fatigue, change in function, or altered mental state, commonly trigger broad diagnostic testing, leading to incidental positive results and subsequent prescribing of antimicrobials. Antimicrobial use often continues despite negative test results.^221^ Almost half of the urine culture orders in nursing homes are due to inappropriate indications, like changes in mental status (32%) and in urine color, odor, or sediment (17%).^222^ Overuse and misuse of urine tests can lead to substantial overprescribing of antimicrobials in nursing homes.

### Approaches to diagnostic stewardship with the laboratory

Nursing homes, particularly those as part of a larger chain or in the same geographic region, may consider working with their contracted laboratory partners to implement aspects of diagnostic stewardship (similar to acute care hospitals), which include pre-analytic (test ordering), test processing, analytic, or post-analytic (test reporting).^223^ In the analytic or processing phase, prespecified criteria like presence of pyuria (i.e., white blood cells) on urinalysis may be used to determine whether a urine culture should be processed by the laboratory. In the post-analytic or reporting phase, the laboratory may report mixed urine cultures as possibly contaminated and use cascade reporting of antimicrobials to aid appropriate antimicrobial choices.^224^ Alternatively, the laboratory may use the high negative predictive value of a normal test result to stop unnecessary antimicrobials (e.g., stopping antimicrobials for UTI in residents without pyuria on urinalysis). The laboratory may also support the nursing home by alerting the facility about cultures growing antimicrobial-resistant organisms of interest, educating HCP about sample collection and transport procedures, describing types of diagnostic tests, developing facility-specific antibiograms, and providing periodic reports on specific diagnostic tests (e.g., number of urine cultures ordered, percent positives, organisms identified on cultures).^72,225^

Consensus guidelines for diagnosis of UTIs in nursing homes recommend the use of a diagnostic algorithm to promote antimicrobial stewardship. This algorithm may also be applicable to older adults and broader post-acute/nursing home populations.^226^ Use of multimodal antimicrobial stewardship interventions in nursing homes have led to a decrease in urine cultures and antimicrobial prescriptions for UTI, with no increase in hospital admissions or mortality.^227^ Clinical decision support tools for evidence-based resident assessment and communication with HCP have also been shown to be effective.^228^ Education of HCP on appropriate criteria for requesting urine cultures should be a component of all interventions.^229^ Education may be delivered as a dialogue tool, involving reflection and communication, case-based educational sessions to address knowledge gaps, or collaborative programs,^230^workshops, webinars, and coaching calls.^227^

**37. How should HCP be trained in collecting specimens for microbiological culture?**

*Recommendation:* Nursing homes should train HCP and conduct annual competency assessments for when and how to collect clinical specimens for diagnostic testing or culture (e.g., signs and symptoms that may indicate the need for urine collection, nasopharyngeal swab, throat swab; sputum collection, swab sample of frank pus from wound, tracheostomy aspirate, blood culture).

*Rationale:* Sustained improvements in testing practices require an assessment of knowledge and attitudes of HCP related to diagnosis and treatment of various infections.^226^ Nurses and medical assistants may have limited training and knowledge^231^ related to diagnostic laboratory practices, especially those for the microbiology laboratory.

Education of HCP on this topic should address specimen collection, transport, and processing. Training and competency assessment for collection should address the importance of reducing the risk of collecting contaminated samples, emphasizing differences between infection and colonization, and should explain that, except for blood cultures, most specimens collected for microbiological cultures are from nonsterile sites and are therefore expected to yield growth. In addition to a handbook developed by the CDC,^232^ many healthcare systems and commercial laboratories offer free written protocols for specimen collection, transport, and processing. These written protocols can be adapted to use as part of an annual competency assessment. Commercial vendors may have similar documentation available for purchase.

### Antimicrobial stewardship

**38. Who should be involved in supporting a nursing home’s antimicrobial stewardship program (ASP)?**

*Recommendation:* The nursing home’s ASP should be supported by, at a minimum, the IP, administrative and medical leadership including the medical director, a consulting pharmacist, and leadership from nursing (see **1**, **2**, and **3**).

*Rationale:* While within CMS regulatory guidance, the ASP program falls under the larger IPC program.^18^ The IP should partner with clinical leaders and the pharmacy to implement ASP activities. In addition, QAA committee members will have responsibility for overseeing ASP activities as part of their larger support for the IPC program.

A nursing home may decide to form a dedicated antimicrobial stewardship committee that includes leadership, pharmacy, and frontline personnel in the development and implementation of specific initiatives, as well as physicians, nurse practitioners, dispensing pharmacists, nurses, nurse aides, allied health professionals, and representatives from the resident and family council.^233^ The specific functions of an antimicrobial stewardship committee should be tailored to the needs and activities within the nursing home, some of which can be determined in facility assessment. Antimicrobial stewardship initiatives should be incorporated into the nursing home’s QAPI program to achieve common goals around resident safety.

**39. What strategies are effective for improving antibiotic use in nursing homes?**

*Recommendation:* Nursing home ASPs should:

1. Have antimicrobial use protocols and systems for monitoring antimicrobial use.
2. Provide regular feedback to prescribing clinicians on the prescribing of antimicrobials.
3. Combine feedback with education to reduce inappropriate antimicrobial use in nursing homes
4. Consider using peer comparison audit and feedback to make clinicians aware of their prescribing habits.

*Rationale:* The CDC has identified key components necessary for the successful implementation of nursing home ASP.^225^ However, CMS regulatory requirements for nursing homes specify antimicrobial use protocols and a system to monitor antimicrobial use as primary activities that should be in place for a nursing home ASP.^13^ Antimicrobial use protocols should provide the following for infections that are common among nursing home residents: diagnostic criteria, appropriate use and interpretation of diagnostic tests, suggestions for supportive care, recommendations for empiric antimicrobials, and length of antimicrobial therapy.^233^ Systems to monitor antimicrobial use may take several forms, including summarizing antimicrobial starts or days of therapy, preparing an antibiogram, or conducting surveillance for *C. difficile* or MDROs.^18^ Supplementary Material, Table 4 details resources to support implementation of ASPs in nursing homes.

**40. What are effective strategies for implementing ASP policies and metrics of success?**

*Recommendation:* Nursing homes should provide all clinical HCP, including physicians, nurse practitioners, nurses, nurses, nurse aides, and allied health professionals with multidisciplinary education about antimicrobial stewardship principles and antimicrobial use protocols.

*Rationale:* Among the array of strategies used to implement ASP policies, education is foundational for prescribers, nurses, nurse aids, residents, and family members. Physicians and pharmacists, who may work with nursing home(s) for a longer length of time, are important to the continuity and sustainability of the ASP. As with other quality improvement efforts, high turnover among nurse aides, nurses, and nursing leadership may cause programs to focus on re-education efforts, which can erode enthusiasm and hinder effective ASP activities.^79,142^ Several evidence-based strategies to support ASPs in nursing homes that build on education may be implemented in sequence or in parallel.^234,235^ For example, a multifaceted quality improvement study that framed antimicrobial stewardship as a resident safety issue was associated with a reduction in antimicrobial use, including fluroquinolones, and the number of urine culture collected. An array of resources were developed from that study, including antimicrobial use protocols suitable for nursing home settings.^236^

**41. What are the protocols for identifying, assessing, and potentially deprescribing antibiotics for newly transferred residents?**

*Recommendation:* In collaboration with referral hospitals, nursing homes should implement a process of medication review upon admission or return of a resident to avoid unnecessary treatments. The process should include identifying antimicrobial prescription, assessing its appropriateness, and discontinuing the prescription if deemed unnecessary.

*Rationale:* Over 10% of nursing home residents were shown to be started on antimicrobials outside the nursing home setting.^237^ CMS regulations require that nursing homes make sure that each resident’s regimen be free from unnecessary medications. Unnecessary medications are defined as any used in excessive dosing (including duplicate therapy); for excessive duration; without adequate monitoring; without adequate indications for its use or in the presence of adverse consequences which indicate the dose should be reduced or discontinued; or any combination of the above-mentioned reasons. Antimicrobials prescribed to residents by HCP from outpatient settings or emergency departments for UTIs are likely to be inappropriate compared with those initiated in the nursing home setting.^237^ This underscores the importance for the nursing home to implement protocols to identify antimicrobials started on residents by outside prescribers, assess the indications for those agents, and potentially discontinue or deescalate inappropriate prescriptions.

Antimicrobial stewardship during care transitions follows the same principles to optimize antimicrobial prescribing and to prevent antimicrobial-related adverse events. A nursing home should incorporate review of antimicrobials prescribed to residents into the existing workflow and processes used for admissions and transfers. This process includes obtaining information on recent or current antimicrobial exposure as outlined in recommendations for interfacility communication during resident/patient transfers (see **10**). HCP who are responsible for accepting a resident for admission or transfer specifically should assess for new medications or changes in existing prescriptions, ideally prior to the resident’s physical arrival to the facility. If this is not feasible, a nursing home may consider implementing an antimicrobial time-out within 1 to 2 days of the resident’s arrival. An antimicrobial time-out is an active assessment of the continued need and appropriateness of an antimicrobial prescription at a specific point after its initiation. The process calls for practitioners to reassess the patient’s overall clinical presentation, response to therapy, and laboratory data (including culture and sensitivity results) and to consider whether antimicrobials may be de-escalated or stopped altogether. Nurses may identify a new antimicrobial prescription and perform an initial assessment of a resident’s condition. Responsibility for reviewing the need to continue, change, or stop antimicrobials and other medications should rest with admitting practitioners, consultant pharmacists, and the medical director.

**42. What is the role of external consultants in a nursing home’s ASP?**

*Recommendation:* External partners and/or consultants may serve as antimicrobial stewardship experts for nursing home ASPs, especially when the nursing home antimicrobial stewardship team lacks such expertise. These individuals may contribute toward development of antimicrobial use protocols, processes for tracking antimicrobial use, data analyses and interpretation, providing specific feedback for further improvement, and/or educating HCP, residents, and families.

*Rationale:* Several studies have demonstrated the value of external partners as collaborators for nursing home ASPs (see Supplementary Material, Table 4). Felsen and colleagues reported that a hospital-based expert team developed educational modules for nursing homes, supported initial implementation of those modules, and then transitioned that role to the nursing home HCP.^81^ Sloane and colleagues described an intervention in which dissemination of educational materials related to antimicrobial use was the responsibility of the nursing home leadership in one arm of their study and a medical group in another arm.^79^ Both studies showed decreases in antimicrobial use following the intervention.

The CDC Core Elements of Antimicrobial Stewardship for Nursing Homes recommends involving infectious diseases consultants or consultant pharmacists with training in antimicrobial stewardship.^225^ Indicative of the potential benefits, an on-site infectious diseases consultation service at a 160-bed nursing home reduced systemic antimicrobial use by 30% and rate of positive *C. difficile* tests.^76^ Additionally, an integrative review reported that involvement of infection diseases consultants supported effective antimicrobial stewardship interventions in nursing homes.^235^

The nursing home may consider partnering with ASPs at the hospitals within their referral network or developing relationships with infectious diseases consultants in their community. Furthermore, some public health departments may have antimicrobial stewardship experts who can assist the nursing home with program development and implementation. Dispensing pharmacies can also work with the nursing home to develop facility-specific antimicrobial use reports and to assess antimicrobial orders for safety and appropriateness (i.e., drug-drug interactions; avoidance of drug-bug mismatch, length of therapy, potential for intravenous to oral conversion, and/or de-escalation). These partnerships are especially important when the nursing home antimicrobial stewardship team lacks antimicrobial expertise.

For a successful collaboration, a nursing home must have leadership support, including administrative and medical leadership along with regular communication and interactions between the nursing home and external consultant. Metrics should assess ASPs in general and the effectiveness of the external partners and/or consultant(s). Several metrics can be used to assess ASPs in nursing homes, including days of therapy and antimicrobial starts per month.^233^ Although these data offer an objective means to evaluate the external consultant(s), qualitative data also are important, including consideration for the relationship between the external consultant(s) and the nursing home leadership and HCP, with specific consideration as to frequency and ease of communication as well as the timeliness and clarity of recommendations.

### Future research

Clinical trials in nursing homes and cluster randomized trials are needed to best understand effective solutions in this specialized setting. Examples of large-scale randomized clinical trials in nursing homes have included video-enhanced advanced planning,^239^ the comparative effectiveness of various types of influenza vaccines,^217,218^ and an evaluation of universal chlorhexidine bathing soap and nasal iodophor on infection-related hospitalizations.^217,218,238,239^

## Supporting information

Mody et al. supplementary material 1 Mody et al. supplementary material

Mody et al. supplementary material 2 Mody et al. supplementary material

## Supplementary material

To view supplementary material for this article, please visit https://doi.org/10.1017/ice.2025.10252
