# Supplementary material for: Multisociety guidance for infection prevention and control in nursing homes
Source: Infect Control Hosp Epidemiol. 2025 Oct 28;46(11):1069–96. doi: 10.1017/ice.2025.10252 (PMC12620066; doi:10.1017/ice.2025.10252)
Supplement: Mody et al. supplementary material 1 — Mody et al. supplementary material [file S0899823X25102523sup001.docx]

Supplementary Material:

Infection Prevention in Nursing Homes

Contents

[Table 1. Recommendations 2](#_Toc210315424)

[Table 2. Terminology and Acronyms 11](#_Toc210315425)

[Table 3. Resources for Nursing Home Infection Prevention and Antimicrobial Stewardship Programs 13](#_Toc210315426)

[Table 4. Descriptions of External Partnering Approaches for Nursing Home Antimicrobial Stewardship Programs 15](#_Toc210315427)

[Table 5. Differences between Competency Checks and Observational Audits 16](#_Toc210315428)

[Table 6. Potential Supplemental Infection Prevention and Control Practices: Examples and Evaluations 17](#_Toc210315429)

[Table 7. Quick Reference for Recommendations 18](#_Toc210315430)

[Table 8. Search strategies, exclusion criteria, and PRISMA 19](#_Toc210315431)

[Search strategies 19](#_Toc210315432)

[PubMed 19](#_Toc210315433)

[Embase 22](#_Toc210315434)

[Exclusion criteria 24](#_Toc210315435)

[Preferred reporting items for systematic reviews and meta-analyses (PRISMA) 25](#_Toc210315436)

[References 26](#_Toc210315437)

# Table 1. Recommendations

| **Infection prevention and control program (IPC program)** | | |
| --- | --- | --- |
| **Leadership** | | |
| 1 | What resources (physical, human, financial) are needed to meet the goals of the nursing home’s IPC program? | The resources for a nursing home’s IPC program should include:  At least one infection preventionist (IP) to manage the infection prevention and control (IPC) program who:  Has ongoing, specialized training in IPC that is financially supported by the nursing home  Demonstrates commitment to ongoing continuing education in IPC to remain current in developments and strategies to optimize the IPC program  Has clinical and/or public health experience  Is an effective communicator, educator, leader, mentor, and collaborator  Receives training in leading and managing programs  Sufficient dedicated time for the IP(s) to manage the IPC program based on the complexity of the resident population and services provided:  At least one full-time equivalent (FTE) IP, if the facility has more than 100 licensed beds or provides onsite ventilator or hemodialysis services  At least 0.5 FTE IP (20 IP hours per week), if the facility has fewer than 100 beds and does not provide on-site ventilator or hemodialysis services  Adequate staffing (e.g., nursing, clinical) and supplies (e.g., personal protective equipment [PPE], alcohol-based hand sanitizer [ABHS], US Environmental Protection Agency (EPA)-registered disinfectants) to allow healthcare personnel (HCP) to follow all recommended IPC practices  Dedicated time for personnel to receive regular job-specific IPC education and demonstrate competency through assessment (see 14 and 15)  Access to information technology training and infrastructure (e.g., integrated electronic health records, software applications, internet access) to support facility-level surveillance activities and access to public health surveillance programs  Access to expert advice, learning collaboratives, and professional associations specific to IPC (see 6 and 42). |
| 2 | To whom should the nursing home IP report? | The nursing home IP should report to a designated person in administrative and medical leadership who has knowledge relevant to regulatory and resource needs for the IPC program.  The IP should be a member of the Quality Assessment and Assurance (QAA) committee to integrate IPC activities within the quality assessment and performance improvement programs.  To be successful, IPC programs require visible and tangible support from all levels of nursing home personnel:  Administrative and medical leadership and the medical director should actively participate in IPC program activities to provide appropriate resources and training to support the implementation of IPC policies and procedures  Nursing homes should clearly define the IP position and include dedicated time for the IP in IPC training, continuous education, and modes of communication with facility personnel, including leadership  Nursing homes should evaluate IPC program surveillance reports and practices using the Quality Assurance Performance Improvement (QAPI) process. |
| 3 | How can a nursing home support the continuity of its IPC program? | Nursing homes should implement strategies to retain and mentor HCP for IPC program continuity so that the IPC program is not dependent on one individual:   1. Prioritize and invest in personnel retention strategies, including competitive wages and benefits for the IP 2. Provide ongoing, job-specific IPC training due to the likelihood that turnover of HCP leads to decreasing effectiveness of the IPC program (see 14 and 15). 3. Establish a mentoring program to foster interest in IPC :    1. Identify individuals who participate in quality improvement initiatives and/or demonstrate interest in IPC in interactions with nursing/clinical supervisors    2. Provide incentives for both mentors and mentees 4. Encourage and support participation in public health activities, local Association for Professionals in Infection Control and Epidemiology (APIC) Chapter meetings, and educational offerings from the Society for Healthcare Epidemiology of America (SHEA) and other professional organizations that work in IPC 5. Develop processes for succession planning, transitions, and cross-training for the activities that support the IPC program (e.g., conducting surveillance, developing IPC policies, implementing antimicrobial stewardship) 6. Reduce decision fatigue with checklists and standard processes 7. Celebrate success and foster a team atmosphere. |
| **Risk assessment** | | |
| 4 | How should a nursing home perform an IPC risk assessment? | 1. The nursing home should perform a risk assessment annually to determine the resources needed to identify and reduce the risk for infections among residents and HCP. 2. The nursing home should assess IPC risk factors at the following levels:    1. Resident-level (person), such as ventilator use or the presence of an indwelling catheter or other medical device    2. Process-level (intervention), such as HCP compliance with hand hygiene, vaccination, and PPE use    3. Facility-level, such as location, access to services, and physical infrastructure. |
| **Working partners** | | |
| **Internal working partners** | | |
| 5 | How should the nursing home’s IPC program engage with facilities management? | To proactively manage potential IPC concerns, particularly as they relate to water management, airflow, air filtration, air disinfection, and construction, the IPC program should provide expert input and/or consultation to the facility management team, which may include a facility engineer, maintenance director, and/or industrial hygienist. |
| **External working partners** | | |
| 6 | What should nursing homes consider when deciding whether to hire an infectious diseases or IP consultant? | Nursing homes may consider hiring external infectious diseases or IP consultants if the IPC risk assessment (see 4) reveals resident-level risk factors or process-level practice implementation gaps that require additional expertise (see 42). |
| 7 | How should the IPC program engage with the hiring and responsibilities of contract services? | Nursing homes should involve the IPC program in:   1. Identifying IPC risks related to the proposed services (e.g., wound care, podiatry) 2. Participating in hiring considerations and defining contractors’ responsibilities 3. Educating contractors about IPC policies and protocols 4. Monitoring contracted services’ compliance with IPC protocols. |
| 8 | How should nursing homes verify IPC training and vaccination status for contract employees and consultants? | Nursing homes should ensure that the contract and on-boarding processes for all contract employees and consultants include provisions requiring appropriate documentation of IPC training and vaccination status. |
| 9 | What relationship should nursing homes have with local and state public health departments? | Nursing homes should:   1. Develop a relationship with local/regional and state public health departments for support, guidance, and collaboration with HCP within the local and regional healthcare continuum. 2. Comply with reporting of cases and outbreaks of infectious diseases are required by local and state public health departments and institutional jurisdictions. Public health departments can help facilities in their efforts to prevent and control pathogen transmission. 3. Partner with public health departments, local hospitals, and other healthcare organizations in quality improvement and safety collaboratives to support antimicrobial stewardship, to prevent infections, outbreaks, and the spread of MDROs, and to improve resident outcomes. |
| 10 | What IPC-specific information should be communicated during resident/patient transfers? | Nursing home HCP involved in resident transfers to or from hospitals, emergency departments, and primary care settings should be proficient in communicating and receiving IPC-specific information, including the resident/patient’s:   1. History of colonization or infection with antimicrobial-resistant organisms 2. Relevant microbiological data, including cultures and susceptibilities 3. Pending test results 4. The need for and type of Transmission-Based Precautions 5. The presence of indwelling medical devices, wounds, diarrhea, or uncontained secretions 6. Current skin conditions 7. Recent or current antimicrobial exposure 8. Vaccination status for relevant vaccines (e.g., influenza, pneumococcus, COVID-19). |
| **Occupational health** | | |
| 11 | How should nursing homes prevent the transmission of infectious illness from HCP to residents and other HCP? | 1. Nursing home personnel and individual HCP, including contractors, consultants, and others who enter the nursing home but may not be directly employed by it, are responsible to adhere to federal, state, and local requirements concerning:    1. Vaccinations:       1. HCP should receive recommended vaccinations or have documented evidence of immunity against vaccine-preventable disease       2. Nursing homes should:          1. Enforce vaccination policies in keeping with vaccine recommendations, including exemptions for medical contraindications and those specified by state and federal regulations          2. Track vaccination status of HCP (see 16)          3. Utilize programs and resources to improve vaccine uptake (see 12)    2. Reporting to public health authorities when an illness identified in the nursing home or among HCP has public health implications or is required to be reported (see 9). 2. Nursing homes should implement policies and processes and that:    1. Promote timely reporting by HCP to the nursing home of signs, symptoms (e.g., fever, cough, diarrhea, vomiting, draining skin lesions), or diagnosed illnesses that may represent a risk to residents and other HCP    2. Support HCP with acute infectious illness to adhere to work restrictions to prevent spread of illness to others in the facility. |
| 12 | How can nursing homes increase vaccine coverage among HCP? | Nursing home administration, medical leadership, and the medical director should:   1. Identify and implement multimodal interventions to increase HCP acceptance of CDC-recommended vaccines 2. Consider the use of educational campaigns and strategies such as onsite delivery of vaccines, time off for receiving and recovering from vaccination, and other ways to promote vaccine uptake and to improve vaccine confidence. |
| **Healthcare-associated infection (HAI) surveillance** | | |
| 13 | How should a nursing home IPC program decide which symptoms, syndromes, and microorganisms to include in its surveillance program? | Nursing homes should:   1. Establish priorities for routine surveillance of HAIs in the nursing home based on the needs of the facility, community risks, and regulatory requirements 2. Adopt standardized definitions and methods of reporting for HAI surveillance. |
| **Healthcare personnel (HCP) training, monitoring, auditing, and feedback** | | |
| 14 | What constitutes minimum IPC competency for nursing homes’ frontline (resident-facing) HCP? | Nursing homes should:   1. Select training methods and content that addresses the diversity of the workforce and meets the needs of the HCP being trained 2. Provide job-specific, minimum IPC competency-based training, defined as the “minimum knowledge and skill needed to safely perform a task according to facility standards and policies”   Ensure dedicated time for HCP to receive regular, job-specific IPC education and to demonstrate competency   1. Document demonstrations of competency following IPC training 2. Evaluate competency before provision of care, specific procedures, introduction of new equipment or protocols, and on an as-needed basis to prepare for and respond to an infectious diseases event 3. Conduct competency assessments through direct observations by trained observers or online skills training that include:     1. Initial or core competency training conducted at-hire or during orientation    2. Ongoing competency training done annually or when new skills or knowledge are needed    3. Specialized competency training related to an area of specialization, such as wound care, central line dressing change, or tracheostomy care. |
| 15 | How should nursing homes monitor IPC practices? | Nursing homes should:   1. Monitor HCP adherence to IPC practices as part of implementing IPC policies 2. Assess the availability of supplies at the point of use to support IPC practices 3. Use findings from the annual IPC risk assessment and infection surveillance data to inform which IPC practices to audit. Commonly audited practices include but are not limited to:    1. Hand hygiene    2. Device insertion, maintenance, and removal    3. Cleaning and disinfection of environmental surfaces and reusable medical equipment    4. Use of PPE    5. Vaccination status of residents and HCP 4. HCP who perform practice audits should:    1. Receive training    2. Use standardized tools to support consistent monitoring. |
| 16 | What models are effective in implementing, auditing, and providing feedback on IPC policies and procedures? | To effectively develop, disseminate, and implement IPC practices (including bundled practices and quality improvement interventions), nursing homes should:   1. Engage administrative and clinical HCP leadership (e.g., nursing and providers). If applicable, nursing homes should also include corporate leadership 2. Obtain input from HCP for strategies to implement IPC practices 3. Ensure HCP who are implementing practices have adequate time to receive education, appropriate training, and competency evaluation (see 14) 4. Involve HCP in ongoing evaluation of practice implementation and opportunities for improvement 5. Audit and provide feedback on HCP adherence to recommended practices 6. Establish metrics to evaluate the impact of practice implementation and quality improvement. |
| **Commonly audited practices** | | |
| 17 | What should a nursing home’s hand hygiene program include? | A nursing home’s hand hygiene program should include:   1. Interactive, regular education with demonstrations of technique, auditing, feedback, and access to educational materials 2. Active engagement by the nursing home’s leadership, clinical HCP, and nonclinical HCP in the practice and promotion of hand hygiene 3. Easy access to ABHS (see 23). |
| 18 | How should nursing homes assess HCP knowledge and skill in device insertion, maintenance, and removal? | 1. HCP should be knowledgeable about medical devices (e.g., central lines, indwelling urinary catheters, percutaneous gastrostomy tubes, tracheostomy tubes) including:    1. The risks associated with their use    2. Recommended IPC practices during placement, maintenance, and removal. 2. Nursing homes should document:    1. The presence, indication for, and duration of a medical device    2. Regular assessment of the ongoing need for a device, presence of signs or symptoms of infection or device malfunction, and opportunities for early and prompt removal    3. Adherence to the recommended steps during insertion, maintenance, and removal of the medical device 3. Nursing homes may conduct audits using standardized forms or audit forms tailored to the needs and processes of the nursing home. |
| 19 | How should nursing homes conduct environmental cleaning and disinfection? | Nursing homes should:   1. Have clearly written policies on the processes and time involved in cleaning and disinfection of environmental surfaces in shared areas, and residents’ rooms, and for reusable medical equipment 2. Ensure that written policies address the frequency of both routine cleaning and disinfection practices, and cleaning and disinfection practices during outbreak situations (see 30) 3. Audit practices for equipment and areas that are cleaned and disinfected, such as frequency and adequacy of cleaning and adherence to contact time (how long a disinfectant remains wet on a surface) 4. Assess availability of appropriate cleaning and disinfection supplies at the point-of-care, ensuring that products are EPA-registered as effective for the purpose for which they are being used (see 31) 5. Use objective methods for evaluation of routine environmental cleaning, which may include direct observation, fluorescent markers, or adenosine triphosphate (ATP) bioluminescence 6. Focus on HCP education and training and provide regular performance feedback. |
| 20 | How should nursing homes ensure proper use of PPE? | Nursing homes should:   1. Develop a written policy for Standard and Transmission-Based Precautions that describes the types of PPE, indications for use, and proper steps for donning (putting on) and doffing (removing) the PPE 2. Provide HCP with ready access to PPE, including gowns, gloves, eye protection, surgical masks, and respirators 3. Ensure HCP properly select and use PPE based on the nature of the resident interaction and the potential for exposure to blood, body fluids, or infectious material 4. Monitor adherence to practices and provide feedback (see 22) 5. Make PPE available at the point-of-care when residents are placed on Transmission-Based Precautions. |
| 21 | What should nursing homes’ vaccination policies include? | Nursing homes should have written, up-to-date policies for vaccination of residents and HCP that include education, training, and monitoring of vaccination acceptance rates. |
| 22 | What methods should nursing homes use to provide feedback on HCP adherence to IPC practices? | 1. Nursing homes should use audit and feedback methods to improve and sustain compliance with evidence-based practices:    1. For individual HCP, nursing homes should use specific, just-in-time feedback on the process being audited    2. At the unit or facility-level, nursing homes should use simplified, aggregated data from audits during rounds, QAA meetings, personnel newsletters, and reporting huddles. 2. Nursing homes should train HCP who conduct these audits to use standardized tools and definitions. 3. Nursing homes should provide feedback to HCP on signs of potential lapses in IPC practices during care. |
| **Environment of care** | | |
| 23 | Where should nursing homes place alcohol-based hand sanitizer (ABHS) dispensers? | Nursing homes should:   1. Place ABHS dispensers where they are easily accessible at a room’s entry and at the point of care 2. Install ABHS dispensers in accordance with local fire regulations 3. Have a hand hygiene program that includes:    1. Widespread availability of hand hygiene products throughout the facility for use by HCP, visitors, and residents    2. Engagement of HCP in selection and feedback on products |
| 24 | How should nursing homes handle laundry and linens for IPC? | Nursing homes should:   1. Use industrial laundry (onsite or offsite) to process laundry and linens. Exceptions may be made for clothing that is laundered by the resident; however, laundry machines used onsite for clothing that is laundered by the resident or family should be disinfected and maintained in accordance with the laundry machines’ MIFUs 2. Store clean laundry and linens in a location that protects them from environmental contamination 3. Educate HCP on safe practices, including PPE selection, when handling and/or changing used linens (e.g., gowns and gloves when handling linens that are grossly soiled) 4. Require HCP to wear gowns and gloves when changing bed linens of residents who are on Enhanced Barrier or Contact Precautions to prevent contamination of clothing and subsequent transmission to other residents. |
| **Outbreak preparedness and response** | | |
| 25 | What strategies should nursing homes use to detect and respond to outbreaks? | Nursing homes should:   1. Be aware of viruses and other pathogens circulating in the community 2. Understand how pathogens that typically cause outbreaks enter and spread within a facility and how to implement pathogen-specific symptom screening 3. Educate HCP to identify and report symptoms (residents’ symptoms or their own) that may be consistent with transmissible pathogens 4. Implement sick leave policies that promote timely reporting of illness and appropriate action (see 11) 5. Implement early (point-of-care) diagnostic testing to identify pathogens 6. Implement appropriate Transmission-Based Precautions based on symptoms, while awaiting a resident’s diagnosis 7. Communicate with referral hospitals and public health departments 8. Vaccinate residents and HCP (see 8 and 11) 9. Identify approaches for the facility’s access to and use of early therapeutics. |
| 26 | How should nursing homes use point-of-care testing to detect and control the spread of respiratory pathogens? | Nursing homes should:   1. Have the capacity to perform point-of-care testing for early detection of viral respiratory pathogens to prevent them from being introduced into the nursing home when community transmission is present and to enable early treatment of residents 2. Liaise with laboratory or infectious diseases consultants regarding selection of point-of-care tests. |
| 27 | How should nursing homes implement respiratory hygiene, cough etiquette, and source control to control the spread of respiratory pathogens? | Nursing homes should have policies and protocols for respiratory hygiene, cough etiquette, and masking for source control to prevent transmission of infection to HCP, visitors, and residents that include:   1. Education of HCP, residents, and visitors in how to prevent transmission of respiratory pathogens 2. Signage and reminders at entrances and in shared areas on hand hygiene and how and when to wear a mask for source control 3. Appropriate and easily accessed supplies (e.g., masks, ABHS) so practices can be followed. |
| 28 | How should nursing homes use ventilation to control the spread of respiratory pathogens? | Nursing homes should:   1. Ensure they are compliant with building code requirements for heating, ventilation, and air conditioning 2. Monitor ventilation systems in accordance with engineers' and manufacturers' recommendations to ensure optimal performance 3. Ensure the IPC program collaborates with facility management, particularly in circumstances when considering implementation of any supplemental strategies to enhance ventilation (e.g., during a facility respiratory pathogen outbreak) (see 5) 4. Have a process in place for isolating residents with pathogens for which an airborne infection isolation room (AIIR) is recommended (e.g., tuberculosis)    1. If an AIIR is not available, the residents should be transferred as soon as is feasible to a facility where an AIIR is available. Place a mask on the resident (if tolerated) and isolate the resident in a private room with the door closed, while awaiting transfer. |
| **Strategies for specific IPC practices in nursing homes** | | |
| **Environmental cleaning and disinfection** | | |
| 30 | How frequently should nursing homes clean and disinfect surfaces in residents’ rooms, shared bathrooms, and shared common areas? | Although the optimal frequency of cleaning and disinfection of areas in nursing homes remains unclear, nursing homes should provide adequate time for HCP to:   1. Routinely clean and disinfect resident rooms, shared bathrooms, and shared common areas at least once a day, paying particular attention to high-touch surfaces with an EPA-registered disinfectant active against the pathogens most likely to contaminate the resident care environment 2. Perform cleaning immediately upon noticing visibly soiled surfaces 3. Increase the frequency of cleaning and disinfection during outbreaks. |
| 31 | What are the cleaning and disinfection considerations for equipment shared among residents (e.g., shower chairs, blood pressure cuffs, mechanical lifts) and residents’ personal belongings? | Nursing homes should:   1. At least daily, clean and disinfect residents’ frequently used items (e.g., canes, walkers, remotes, tablets, phones) 2. After each use, clean and disinfect items that are shared among residents 3. Choose cleaning products that are EPA-registered for the specific cleaning and disinfection purpose 4. Follow the MIFU for equipment and the cleaning products used to avoid damaging existing equipment and objects.   The laundry section (see 24) addresses cleaning of residents’ soft items, as appropriate. Care of residents’ personal hygiene items (e.g., toothbrushes) are outside the scope of this document. |
| **Resident placement and PPE use** | | |
| 32 | How should nursing homes use Transmission-Based Precautions for residents who are chronically colonized with MDROs apply to dining (in-room and group), rehabilitation and therapy (in-room and group), recreational, and other high-contact activities, as well as interactions with residents’ visitors, including students, trainees, and volunteers? | Nursing homes should:   1. Not restrict residents who are chronically colonized with MDROs from visitation, social activities, dining, rehabilitation and therapy, or recreational activities 2. Apply Enhanced Barrier Precautions for residents infected or colonized with MDROs targeted by CDC. Nursing homes may consider, based on its policies, applying Enhanced Barrier Precautions more broadly to include other epidemiologically important MDROs 3. Not require that visitor(s) seeing a single resident who is chronically colonized with an MDRO wear specific PPE, although nursing homes may offer PPE for high-contact care or for care, in which Standard Precautions would require PPE. |
| 33 | How should Transmission-Based Precautions apply to residents unable to tolerate IPC interventions (such as room restriction) implemented as part of outbreak response (e.g., individuals with significant cognitive impairment)? | For residents who are unable to tolerate IPC interventions implemented as part of outbreak response, nursing homes should:   1. Emphasize prevention measures that do not depend on room restriction (e.g., vaccination, therapeutics) to prevent spread among all residents 2. Have HCP routinely assist residents in performing hand hygiene 3. During outbreaks, utilize horizontal IPC approaches, which are intended to control the spread of multiple organisms simultaneously. |
| 34 | How should nursing homes educate residents, families,  and visitors on appropriate IPC practices? | Nursing homes should:   1. Educate and engage residents, families, and visitors in adoption of appropriate practices for hand hygiene, respiratory hygiene, PPE, antibiotic use, vaccination, and practices for the prevention and control of emerging infections and outbreaks 2. Post IPC policies and reminders in nursing home reception areas to reinforce interactions with HCP. Nursing homes may consider including signage at the entrance to the building, at reception, in family newsletters, on digital information screens, and on resident-used tablets and computers. |
| 35 | How should nursing homes assess whether to adopt evolving IPC practices to complement current IPC efforts? | No recommendation. |
|  | **Diagnostic stewardship** |  |
| 36 | What is the role of the laboratory in supporting diagnostic stewardship in nursing homes? | In partnering with microbiology laboratories, nursing homes should incorporate the principles of diagnostic stewardship rules in the ordering, interpreting, and reporting. |
| 37 | How should HCP be trained in collecting specimens for microbiological culture? | Nursing homes should train HCP and conduct annual competency assessments for when and how to collect clinical specimens for diagnostic testing or culture (e.g., signs and symptoms that may indicate the need for urine collection, nasopharyngeal swab, throat swab; sputum collection, swab sample of frank pus from wound, tracheostomy aspirate, blood culture). |
|  | **Antimicrobial stewardship** |  |
| 38 | Who should be involved in supporting a nursing home’s antimicrobial stewardship program (ASP)? | The nursing home’s ASP should be supported by, at a minimum, the IP, administrative and medical leadership and the medical director, the consulting pharmacist, and leadership from nursing (see 1-3). |
| 39 | What strategies are effective for improving antibiotic use in nursing homes? | Nursing home ASPs should:   1. Have antimicrobial use protocols and systems for monitoring antimicrobial use. 2. Provide regular feedback to prescribing clinicians on prescribing of antimicrobials. 3. Combine feedback with education to reduce inappropriate antimicrobial use in nursing homes 4. Consider using peer comparison audit and feedback to make clinicians aware of their prescribing habits. |
| 40 | What are effective strategies for implementing ASP policies and metrics of success? | Nursing homes should provide all clinical HCP, including physicians, nurse practitioners, nurses, nurse aides, and allied health professionals with multidisciplinary education about antimicrobial stewardship principles and antimicrobial use protocols. |
| 41 | What are the protocols for identifying, assessing, and potentially deprescribing antibiotics for newly transferred residents? | In collaboration with referral hospitals, nursing homes should implement a process of medication review upon admission or return of a resident to avoid unnecessary treatments. The process should include identifying antimicrobial prescription, assessing its appropriateness, and discontinuing the prescription if deemed unnecessary. |
| 42 | What is the role of external consultants in a nursing home’s ASP? | External partners and/or consultants may serve as antimicrobial stewardship experts for nursing home ASPs, especially when the nursing home antimicrobial stewardship team lacks such expertise. These individuals may contribute toward development of antimicrobial use protocols, processes for tracking antimicrobial use, data analyses and interpretation, providing specific feedback for further improvement, and/or educating HCP, residents, and families. |

# Table 2. Terminology and Acronyms

| **Term** | **Used as** | **Also called** |
| --- | --- | --- |
| Nursing home | A facility that provides some skilled nursing care | Long-term care facilities, skilled nursing facility, home for the aged (Europe), elder care home (Europe), dementia care, memory care  Continuing care retirement communities may have nursing home units in addition to independent and assisted living settings  May also refer to, as determined to be appropriate: immediate care facilities, immediate care facilities for intellectual disabilities, rehabilitation skilled nursing facilities, and ventilator skilled nursing facilities |
| Infection prevention and control program | Facility-specific configuration of coordinated staffing and resources to uphold infection prevention and control in a nursing home | Infection control program  IPC Program |
| Administrative and medical leadership |  | Medical director, epidemiologist, administration |
| Infection preventionist |  | Infection prevention and control leader |
| Epidemic and non-epidemic | In the region |  |
| Outbreak and non-outbreak | In the facility |  |
| QAA committee | Infection control and control committee   - QAPI - QI/QIO |  |
| **Acronym** | **Term** | |
| ABHS | Alcohol-based hand sanitizer | |
| AHRQ | Agency for Healthcare Research and Quality | |
| AIIR | Airborne infection isolation room | |
| AMDA | Society of Post-Acute and Long-Term Care Medicine | |
| APIC | Association for Professionals in Infection Control and Epidemiology | |
| ASP | Antimicrobial stewardship program | |
| ATP | Adenosine triphosphate | |
| *C. difficile* | *Clostridium difficile* | |
| CAUTI | Catheter-associated urinary tract infection | |
| CDC | Centers for Disease Control and Prevention | |
| CLABSI | Central line-associated bloodstream infection | |
| CMS | Centers for Medicare and Medicaid Services | |
| CNA | Certified nursing assistant | |
| DNA | Deoxyribonucleic acid | |
| EPA | US Environmental Protection Agency | |
| FTE | Full-time equivalent | |
| GLC | Guidelines Committee | |
| GRADE | Grading of Recommendations Assessment, Development and Evaluation | |
| HAI | Healthcare-associated infection | |
| HCP | Healthcare personnel | |
| HVAC | Heating, ventilation, and air conditioning | |
| IDSA | Infectious Diseases Society of America | |
| IP | Infection preventionist | |
| IPC | Infection prevention and control | |
| MDR-GNB | Multidrug-resistant gram-negative bacteria | |
| MDRO | Multidrug-resistant organism | |
| MIFU | Manufacturer’s instructions for use | |
| MRSA | Methicillin-resistant *Staphylococcus aureus* | |
| NHSN | National Healthcare Safety Network | |
| PICO | Population, intervention, control, and outcomes | |
| PPE | Personal protective equipment | |
| QAA | Quality Assessment and Assurance | |
| QAPI | Quality Assurance Performance Improvement | |
| RN | Registered nurse | |
| RSV | Respiratory syncytial virus | |
| SHEA | Society for Healthcare Epidemiology of America | |
| US | United States | |
| UTI | Urinary tract infection | |

# Table 3. Resources for Nursing Home Infection Prevention and Antimicrobial Stewardship Programs

| **Source** | **Resource** | **Notes** |
| --- | --- | --- |
| AHRQ | [Toolkit to Improve Antibiotic Use in Long-Term Care](https://www.ahrq.gov/antibiotic-use/long-term-care/index.html) | The Four Moments of Antibiotic Decision Making:   1. How to apply them 2. Presentations and tools to support implementation and antibiotic prescribing by: 3. Developing and improving the ASP 4. Creating a culture of safety around antibiotic prescribing 5. Learning and disseminating best practices for common infectious diseases syndromes. |
| PALTmed (previously AMDA) | [Template for an Antibiotic Stewardship Policy for Post-Acute and Long-Term Care Settings](https://europepmc.org/backend/ptpmcrender.fcgi?accid=PMC5839140&blobtype=pdf) | 1. Policy template for nursing homes to adapt to their facility 2. Discussion of implementation of an ASP in LTC settings 3. List of free resources to support efforts |
| APIC | [Long-Term Care IP Essentials](https://apic.org/course/ltc-ip-essentials/) | IPC training and education course |
| APIC | [LTC-CIP Review Course](https://learnipc.apic.org/ltc-cip-certification) |  |
| Brown | [UNC-SPICE](https://spice.unc.edu/iCAREhttps:/care.brown.edu/why-i-care) |  |
| CDC | [Nursing Homes and Assisted Living Resources](https://www.cdc.gov/longtermcare/index.html) | IPC education tool |
| CDC | [CDC Train](https://www.train.org/cdctrain/training_plan/3814) | Nursing home IP training course |
| CDC | [National Healthcare Safety Network (NHSN) for LTC Facilities](https://www.cdc.gov/nhsn/ltc/index.html) | Introduction video, enrollment instructions, manual of definitions, FAQs |
| CDC | [ICAR: Infection Control Assessment Tools](https://www.cdc.gov/hai/prevent/infection-control-assessment-tools.html) |  |
| CDC | [Infection Control Guidelines Library](https://www.cdc.gov/infectioncontrol/guidelines/index.html) |  |
| CDC | [Implementation of PPE Use in Nursing Homes to Prevent the Spread of MDROs](https://www.cdc.gov/hai/containment/PPE-Nursing-Homes.html) |  |
| CDC | Core Elements for Antibiotic Stewardship in Nursing Homes (1) | 1. Leadership commitment 2. Accountability 3. Drug expertise 4. Action 5. Tracking 6. Reporting 7. Education |
| CMS | [Regulation & Guidance for Long-Term Care Facilities](https://www.cms.gov/nursing-homes/providers-partners/regulations-guidance) | Regulation, guidance, training, and resources for LTC Facilities |
| CMS | Reform of Requirements for Long-Term Care Facilities 2016  Interpretive Guidelines, Guidance to Surveyors for Long-Term Care Facilities  Medicare State Operations Manual, Appendix PP: Interpretive Guidelines for Long-Term Care Facilities n.d. | Requirement for a facility to develop an ASP with protocols and a system to monitor antibiotic use, with support and accountability from leadership via the participation of administrative and medical leadership, the consulting pharmacist, nursing leadership, and other individuals with designated responsibility for the IPC Program |
| CORHA | Principles and Practices for Healthcare Outbreak Response | [CORHA Principles and Practices for Healthcare Outbreak Response \| CORHA](https://www.corha.org/resources/corha-principles-and-practices-for-outbreak-response/) |
| CORHA | HAI Outbreak Notification Framework | [CORHA Framework for Healthcare-Associated Infection Outbreak Notification \| CORHA](https://www.corha.org/resources/corha-framework-for-healthcare-associated-infection-outbreak-notification/) |
| CORHA | C. auris: Recommendations for Healthcare Response | [Candida Auris: Recommendations for Healthcare Outbreak Response \| CORHA](https://www.corha.org/resources/candida-auris-recommendations-for-healthcare-outbreak-response/) |
| CORHA | Influenza: Thresholds for Reporting and Investigating Cases | [CORHA Thresholds for Reporting and Investigating Cases of Influenza \| CORHA](https://www.corha.org/resources/corha-thresholds-for-reporting-and-investigating-cases-of-influenza/) |
| CORHA | CDI: Recommendations for Healthcare Outbreak Response | [CORHA Clostridioides difficile Infection (CDI): Recommendations for Healthcare Outbreak Response \| CORHA](https://www.corha.org/resources/clostridioides-difficile-infection-cdi-recommendations-for-healthcare-outbreak-response/) |
| CORHA | COVID-19: Investigation/Reporting Thresholds and Outbreak Definitions for Healthcare Settings | [Investigation/Reporting Thresholds and Outbreak Definitions for COVID-19 in Healthcare Settings \| CORHA](https://www.corha.org/resources/corha-cste-proposed-investigation-reporting-thresholds-and-outbreak-definitions-for-covid-19-in-healthcare-settings/) |
| CORHA | Nontuberculous mycobacteria: Recommended Practices for Healthcare Outbreak Response | [Nontuberculous mycobacteria: Recommended Practices for Healthcare Outbreak Response \| CORHA](https://www.corha.org/resources/corha-proposed-investigation-reporting-thresholds-and-outbreak-definition-for-extrapulmonary-nontuberculous-mycobacteria-ntm/) |
| CORHA | CRE: Recommendations for Healthcare Outbreak Response | [Carbapenem-resistant Enterobacterales (CRE): Recommendations for Healthcare Outbreak Response \| CORHA](https://www.corha.org/resources/corha-interim-suggested-investigation-reporting-thresholds-and-outbreak-definition-for-carbapenem-resistant-enterobacteriaceae-cre/) |
| CORHA | Scabies: Outbreak Detection, Reporting, and Infection Prevention | - [CORHA General Guidance for the Outbreak Detection and Reporting of Scabies \| CORHA](https://www.corha.org/resources/corha-general-guidance-outbreak-detection-reporting-scabies/) - LTC-specific: [Infection Prevention in Long-Term Care – Scabies \| CORHA](https://www.corha.org/resources/infection-prevention-in-long-term-care-scabies/) |
| OSHA | Occupational requirements for PPE, exposures | - [1910.132 - General requirements](https://www.osha.gov/laws-regs/regulations/standardnumber/1910/1910.132) - [1910.134 - Respiratory protection](https://www.osha.gov/laws-regs/regulations/standardnumber/1910/1910.134) - [1910.1030 - Bloodborne pathogens](https://www.osha.gov/laws-regs/regulations/standardnumber/1910/1910.1030) |
| SHEA | [Implementing ASPs in Post-Acute and LTC Settings](https://learningce.shea-online.org/content/implementing-antibiotic-stewardship-programs-post-acute-and-long-term-care-settings#group-tabs-node-course-default5) | Online course reviews regulatory requirements, resources, and stewardship implementation approaches |
| UCI Health | [Nursing Home Decolonization Toolkit](https://www.ucihealth.org/shield/nursing-home-decolonization-toolkit) | Protocols, resources, educational materials for chlorhexidine bathing and nasal decolonization to reduce infection, hospitalization, and MDROs |

#

# Table 4. Descriptions of External Partnering Approaches for Nursing Home Antimicrobial Stewardship Programs

| **Study** | **Personnel involved in consultations** | **Roles of External Consultants** |
| --- | --- | --- |
| Salem-Schatz (2) | Interdisciplinary group of experts including infectious diseases physician, geriatrician, and independent consultants in quality improvement, organization behavior and change management | - Collaborated with NH colleagues during workshops to develop QI materials and inform QI strategies to implement AS interventions. - Developed a core curriculum focusing on appropriate testing and treatment of UTI incorporating QI principles and specific concepts and strategies for adaptive changes based on complexity science. - Developed and disseminated various educational tools. - Provided ongoing support including a coaching check-in call to facility team leaders by QI experts every 6 weeks. |
| Felsen (3) | Hospital-based AS expert team | - Collaborated with administrative and medical leadership advisory group to develop treatment guidelines for the nursing homes in the community - Assisted with NH to incorporate AS into their daily routine - Created antibiotic tracking tools for NH staff - Regularly reviewed antibiotic data to identify need for additional facility-specific interventions - Worked with microbiology lab to develop NH antibiograms - Coached NH AS team on interpretation of antibiotic data to decide on intervention targets - Summarized antibiotic data and provided quarterly feedback - Developed educational modules and conducting interactive face-to-face sessions (transitioned responsibility over time to NH staff) |
| Sloane (4) | University-based research team | - Developed educational material (videos, posters, pocket cards, information brochure) for promoting antibiotic stewardship interventions - Shared quarterly quality improvement 1-page newsletter to HCP and nursing directors that included prescribing data individually for each participating NH (with deidentified comparison data from other facilities in the study) |
| Stevenson (5) | Infectious diseases physicians via videoconference | Weekly videoconferences between an infectious diseases physician and a multidisciplinary team at rural hospital and connected nursing home. |
| Doernberg (6) | Infectious diseases pharmacist and infectious diseases physician | Performed weekly prospective audit and feedback of prescriptions for UTI (after discussion with the ID physician, the pharmacist conveyed ASP recommendations to primary treating provider via telephone or fax) |
| Zimmerman (7) | University-based clinical and health services researchers (including a physician, nurse, infectious  disease specialist, and experts in nursing home organization) | Physicians and NH nurses   - Provided on-site training to physicians and nursing home nurses on prescribing guidelines and practices. - Shared feedback on prescribing practices and adherence to guidelines - Participated in the monthly nursing home quality improvement team meetings - Facilitated education to residents and families by providing information brochure related to antibiotic prescribing and QI program and attending various gatherings/meetings where the information was being shared. |
| Jump (8) | Infectious diseases Physicians and Nurse Practitioner | Examined residents at the LTCF once each week and remained available for remote consultation the remainder of the week |

# Table 5. Differences between Competency Checks and Observational Audits

|  | **Competency check** | **Observational audits** |
| --- | --- | --- |
| *Location Type* | Controlled environment | Actual work environment |
| *Timing* | Routine, during scheduled orientation or skills fair | Unscheduled, sometimes using secret observers |
| *Awareness of observation* | Yes | No |
| *Education* | Education/Training provided first | No education training provided prior to audit |
| *Frequency* | At hire, and annually  Additional competency check may be needed in unusual situations (e.g., pandemic, outbreaks) | Regular intervals as determined by policy |
| *Feedback* | Provided during demonstration | Provided one-on-one (usually immediately after audit), and documented |
| *Outcome* | Results used for employee file, and evidence of competency for surveys | Used for tracking/trending infection prevention processes and identifying gaps |

# Table 6. Potential Supplemental Infection Prevention and Control Practices: Examples and Evaluations

*This table provides several examples of supplemental activities in nursing home settings that have been evaluated by large-scale, randomized-controlled trials and studies. These examples highlight strategies that may be beneficial when performed correctly, and those that are commonly used but have not shown benefit and should not be encouraged.*

| **Problem** | **Interventions** | **Significance** | **Interpretation** |
| --- | --- | --- | --- |
| Infections, infection-related hospitalizations, and multidrug-resistant organisms (MDROs) (9, 10) | Universal CHG for all routine bed bathing (2% leave-on) and showering (4% rinse off) plus universal nasal iodophor twice daily Monday-Friday every other week | Universal decolonization in nursing homes reduced 1.9 infection-related hospitalizations per month per 100-bed nursing home, and reduced MDRO prevalence (MRSA, VRE, ESBL, CRE) by 30%.  Decreases in infection-related hospitalizations were associated with decreased costs and deaths for nursing home residents. | Universal decolonization with routine CHG bathing and nasal iodophor should be considered for reducing infection-related hospitalizations and MDROs in nursing homes |
| Pneumonia/Respiratory Tract Infections (11, 12) | Oral care techniques and products to protect teeth, gums, tongue, dentures (Mouth Care Without a Battle program) | Sustained improvement in oral and denture hygiene by systematic oral exam in residents of 13 nursing homes    No reduction in pneumonia | Training on oral care techniques and products should be considered for improving oral hygiene.    Oral care was not proven to prevent pneumonia. |
| *Staphylococcus aureus* colonization/infection (13) | Mupirocin nasal ointment vs placebo twice daily for two weeks | 93% successful decolonization with mupirocin vs 15% with placebo in two LTC facilities | Nasal mupirocin should be considered for reducing *S. aureus* colonization |
| Urinary Tract Infections (14) | Two cranberry capsules daily vs placebo | No difference in bacteriuria or UTI in women in 21 nursing homes | Cranberry supplements should not be considered for preventing bacteriuria or UTI. |

#

# Table 7. Quick Reference for Recommendations

| **Topic** | **Recommendations numbers** |
| --- | --- |
| Administration, leadership, medical director, infection preventionist (IP) | 1-2, 12-13, 16-17, 20, 35-38, 42 |
| Antimicrobial stewardship | 1, 3, 9, 13, 35, 38-42 |
| Consultants and contract services | 6-8, 11, 26, 42 |
| Detection and control of respiratory pathogens | 4-6, 11-12, 21, 23, 26-28 |
| Device care | 4, 13, 15, 18 |
| Diagnostic stewardship | 36-37 |
| Diagnostics and testing | 25-26, 35-37, 42 |
| Environmental care, cleaning, and disinfection | 1, 4-5, 14-15, 19-20, 22-24 |
| Facility management (water, airflow and ventilation, construction) | 4-5, 28 |
| Hand hygiene | 1, 4, 6-7, 12, 15-18, 22-23, 27, 33-34, 37 |
| HCP education, training, and competency | 1-4, 6-9, 11, 13-17, 21, 25, 32, 37, 42 |
| HCP illness (screening, reporting, and sick leave) | 11, 25 |
| Laundry and linens | 24, 31-32 |
| Colonization and decolonization | 1, 4, 10, 20, 32, 35, 37 |
| Monitoring, audit, feedback of IPC practices | 1-2, 6-7, 14-15, 17, 19, 20-22, 39, 41 |
| Outbreak preparedness and response | 1, 3, 7, 9-11, 14, 19, 24-28, 30, 32-35 |
| Precautions and PPE | 1, 4, 7-9, 15-16, 18, 20, 24-26, 32-34 |
| Public health | 6, 9, 10-11, 13-15, 21, 27, 31-32, 37, 39, 42 |
| Quality improvement | 1-4, 9, 13-14, 16, 40 |
| Resident, visitor, student, volunteer education | 17, 24, 31-32, 34, 40 |
| Residents’ personal items | 24, 30-31 |
| Residents’ rooms | 4, 18-1, 339, 23, 30-31 |
| Respiratory hygiene, cough etiquette, source control | 7, 11, 27, 34 |
| Risk assessment | 4, 6, 13, 15-16 |
| Shared items and equipment | 7, 14, 19, 31 |
| Shared spaces and common areas | 4, 19, 27, 30, 34 |
| Staff | 1-3, 9, 11, 14, 21, 25, 33-34 |
| Surveillance | 1-4, 9, 13, 15, 18, 22, 39 |
| Transfers | 10, 28, 41 |
| Vaccination | 4, 8, 10-13, 15, 21, 33-34 |

# Table 8. Search strategies, exclusion criteria, and PRISMA

## Search strategies

### PubMed

#### Sets Combined with Nursing Homes Concepts and IP Concepts

((("Nursing Homes"[MeSH Terms] OR "nursing home*"[Title/Abstract] OR "intermediate care*"[Title/Abstract] OR "skilled nursing*"[Title/Abstract] OR ("SNF"[Title/Abstract] OR "SNFS"[Title/Abstract] OR "extended care facilit*"[Title/Abstract]) OR "continuing care*"[Title/Abstract] OR ("Long-Term Care"[MeSH Terms] OR "long term care*"[Title/Abstract] OR "LTC"[Title/Abstract] OR "LTCF"[Title/Abstract]) OR ("Assisted Living Facilities"[MeSH Terms] OR "ASSISTED LIVING"[Title/Abstract]) OR ("homes for the aged"[MeSH Terms] OR "homes for the aged"[Title/Abstract] OR "old age home*"[Title/Abstract] OR "HOMES FOR THE ELDERLY"[Title/Abstract]) OR "ELDER CARE"[Title/Abstract] OR "DEMENTIA CARE"[Title/Abstract] OR "MEMORY CARE"[Title/Abstract] OR ("Independent Living"[MeSH Terms] OR "independent living*"[Title/Abstract] OR "community dwelling*"[Title/Abstract]) OR ("rehabilitation center*"[Title/Abstract] OR "rehabilitation facilit*"[Title/Abstract]) OR "Rehabilitation Centers"[MeSH Terms:noexp] OR "nursing center*"[Title/Abstract] OR "Skilled Nursing Facilities"[MeSH Terms]) NOT ("animal*"[MeSH Terms] NOT ("animal*"[MeSH Terms] AND "human*"[MeSH Terms]))) NOT ("letter*"[Publication Type] OR "editorial*"[Publication Type] OR "comment*"[Publication Type])) AND "english"[Language] AND 2007/01/01:2021/12/31[Date - Publication] AND ((("cross infection"[MeSH Terms] OR "nosocomial infection*"[Title/Abstract] OR "health care associated infection"[Title/Abstract] OR "disease transmission, infectious"[MeSH Terms] OR "infectious disease*"[Title/Abstract]) AND ("prevent*"[Text Word] OR "control*"[Text Word])) OR "infection control"[MeSH Terms] OR ("infection*"[Title/Abstract] AND ("prevent*"[Text Word] OR "control*"[Text Word] OR "reduc*"[Title/Abstract])) OR ("hai reduction*"[Title/Abstract] OR "hai prevention*"[Title/Abstract] OR "Communicable Disease Control"[MeSH Terms:noexp]) OR "infections/prevention and control"[MeSH Terms] OR (("Preventive Health Services"[MeSH Terms:noexp] OR "preventive health"[Title/Abstract] OR "PREVENTATIVE HEALTH"[Title/Abstract]) AND ("infection*"[Text Word] OR "infectious*"[Title/Abstract]))) AND ("EXECUTIVE"[Title/Abstract] OR "EXECUTIVES"[Title/Abstract] OR "ORGANIZATION AND ADMINISTRATION"[MeSH Subheading] OR "leadership"[MeSH Terms] OR "leader*"[Title/Abstract] OR "health personnel"[MeSH Terms:noexp] OR "infection control practitioners"[MeSH Terms] OR "health facility administrators"[MeSH Terms] OR "physician executive*"[MeSH Terms] OR "medical director*"[Title/Abstract] OR "administrative personnel"[MeSH Terms] OR "administrator*"[Title/Abstract] OR "policy maker*"[Title/Abstract] OR "infection preventionist*"[Title/Abstract] OR "governance*"[Title/Abstract] OR ("Risk Assessment"[MeSH Terms] OR "risk assessment*"[Title/Abstract] OR "risk analys*"[Title/Abstract] OR "Risk Factors"[MeSH Terms] OR "risk factor*"[Title/Abstract] OR "risk score*"[Title/Abstract] OR "health correlate*"[Title/Abstract] OR "POPULATIONS AT RISK"[Title/Abstract] OR "POPULATION AT RISK"[Title/Abstract]) OR ("HEALTH RESOURCES"[MeSH Terms] OR "health resource*"[Title/Abstract] OR "health workforce"[MeSH Terms] OR "workforce*"[Title/Abstract] OR "health personnel"[MeSH Terms] OR "PERSONNEL"[Title/Abstract] OR "MANPOWER"[Title/Abstract] OR "personnel turnover"[MeSH Terms] OR "personnel turnover"[Title/Abstract] OR "staff turnover"[Title/Abstract] OR "staff stability"[Title/Abstract] OR (("occupational groups"[MeSH Terms] OR "occupation*"[Title/Abstract] OR "PERSONNEL"[Text Word]) AND "STABILITY"[Title/Abstract]) OR ("FINANCIAL SUPPORT"[MeSH Terms:noexp] OR "financial support*"[Title/Abstract] OR "financial investment"[Title/Abstract] OR "EQUIPMENT AND SUPPLIES"[MeSH Terms:noexp] OR "SUPPLIES"[Title/Abstract] OR "EQUIPMENT"[Title/Abstract] OR "SUPPLY AND DISTRIBUTION"[MeSH Subheading] OR "PERSONAL PROTECTIVE EQUIPMENT"[MeSH Terms] OR "PPE"[Title/Abstract] OR "PERSONAL PROTECTIVE EQUIPMENT"[Title/Abstract] OR "HEALTH CARE EVALUATION MECHANISMS"[MeSH Terms] OR "HEALTH CARE EVALUATION"[Title/Abstract] OR "PROFESSIONAL COMPETENCE"[MeSH Terms] OR "CLINICAL COMPETENCE"[MeSH Terms] OR "competenc*"[Title/Abstract] OR "expertise*"[Title/Abstract] OR "Work Performance"[MeSH Terms] OR "work performance*"[Title/Abstract] OR "job performance*"[Title/Abstract])) OR ("Sentinel Surveillance"[MeSH Terms] OR "Population Surveillance"[MeSH Terms:noexp] OR "surveillance*"[Text Word] OR (("Disease Outbreaks"[MeSH Terms] OR "OUTBREAK"[Text Word]) AND ("detect*"[Title/Abstract] OR "report*"[Title/Abstract])) OR ("mandatory reporting"[MeSH Terms] OR "mandatory report*"[Title/Abstract]) OR (("Government Regulation"[MeSH Terms] OR "government regulation*"[Title/Abstract] OR (("State Government"[MeSH Terms] OR "state government*"[Title/Abstract]) AND "REPORTING"[Text Word]) OR ("centers for medicare and medicaid services, u s"[MeSH Terms] OR "CENTERS FOR MEDICARE AND MEDICAID"[Title/Abstract]) OR ("centers for disease control and prevention, u s"[MeSH Terms] OR "CENTERS FOR DISEASE CONTROL"[Title/Abstract] OR "CDCP"[Title/Abstract] OR "CDC"[Title/Abstract])) AND "reporting*"[Text Word]) OR ("quality assurance, health care"[MeSH Terms] OR "healthcare quality assurance*"[Title/Abstract] OR "quality assessment*"[Title/Abstract] OR "Quality Improvement"[MeSH Terms:noexp] OR "qapi"[Title/Abstract] OR "audit*"[Text Word] OR "Inservice Training"[MeSH Terms] OR "TRAINING"[Title/Abstract] OR "staff development"[MeSH Terms] OR "staff development"[Title/Abstract]) OR ((("educat*"[Title/Abstract] OR "TRAINING"[Title/Abstract]) AND "staff*"[Text Word]) OR "occupational groups"[MeSH Terms] OR "occupational groups"[Title/Abstract] OR "PERSONNEL"[Text Word] OR "physician*"[Text Word] OR "nurs*"[Text Word] OR ("allied health*"[Text Word] OR "worker*"[Title/Abstract] OR "MANPOWER"[Title/Abstract] OR "WORKFORCE"[Title/Abstract] OR "WORK FORCE"[Title/Abstract] OR "personnel*"[Text Word])) OR ("ventilators, mechanical"[MeSH Terms] OR "ventilator*"[Text Word] OR "ventilation service*"[Title/Abstract] OR "RESPIRATOR"[Title/Abstract] OR "RESPIRATORS"[Title/Abstract]) OR ("Dialysis"[MeSH Terms] OR "dialys*"[Text Word] OR "Renal Dialysis"[MeSH Terms])) OR ("POLICY"[Text Word] OR "POLICIES"[Title/Abstract] OR "Organizational Policy"[MeSH Terms]) OR ("occupational health"[MeSH Terms] OR "industrial hygien*"[Title/Abstract] OR "industrial health"[Title/Abstract] OR "employee health"[Title/Abstract] OR "occupational health"[Title/Abstract] OR "INFRASTRUCTURE"[Title/Abstract] OR "Health Facility Environment"[MeSH Terms] OR "health facility environment*"[Title/Abstract] OR "patients rooms"[MeSH Terms] OR "PATIENT'S ROOMS"[Title/Abstract] OR "PATIENT ROOMS"[Title/Abstract] OR "facility characteristic*"[Title/Abstract] OR "physical plant*"[Title/Abstract]) OR ("Water Quality"[MeSH Terms] OR "water qualit*"[Title/Abstract] OR "Water Supply"[MeSH Terms:noexp] OR "Water Supply"[Title/Abstract] OR "Water Microbiology"[MeSH Terms] OR "potable water"[Title/Abstract] OR "facility water"[Title/Abstract] OR "water system*"[Title/Abstract] OR "water management"[Title/Abstract]) OR ("environment, controlled"[MeSH Terms] OR "controlled environment*"[Title/Abstract] OR "air flow"[Title/Abstract] OR "air flow"[Title/Abstract] OR "AIRFLOW"[Title/Abstract] OR "AIR CONDITIONING"[Text Word] OR ("Air Movements"[MeSH Terms:noexp] OR "air movement*"[Title/Abstract]) OR ("Air Microbiology"[MeSH Terms] OR "air microbiolog*"[Title/Abstract]) OR ("air pollution, indoor"[MeSH Terms] OR "indoor air pollut*"[Title/Abstract] OR "INDOOR AIR QUALITY"[Title/Abstract]) OR ("Air Pollutants"[MeSH Terms:noexp] AND "indoor*"[Text Word]) OR ("Ventilation"[MeSH Terms] OR "building ventilation*"[Title/Abstract]) OR ("airborne*"[Title/Abstract] OR "AIR BORNE"[Title/Abstract]) OR ("Air Filters"[MeSH Terms] OR "air filter*"[Title/Abstract] OR "air purifier*"[Title/Abstract])) OR ("Environmental Monitoring"[MeSH Terms:noexp] OR "environmental monitor*"[Title/Abstract] OR "environmental surveillance*"[Title/Abstract] OR "environmental design"[Title/Abstract] OR "Environment Design"[MeSH Terms] OR "Environment Design"[Title/Abstract] OR "healthy place*"[Title/Abstract] OR "environmental testing"[Title/Abstract] OR "Equipment Contamination"[MeSH Terms] OR "equipment contamination*"[Title/Abstract] OR "environment contamination"[Title/Abstract] OR "environmental contamination"[Title/Abstract]) OR ("Needs Assessment"[MeSH Terms] OR "needs assessment*"[Title/Abstract] OR "Consultants"[MeSH Terms] OR "consultant*"[Title/Abstract] OR "advisory service*"[Title/Abstract] OR "contractor*"[Title/Abstract] OR "contract employee*"[Title/Abstract]) OR (("Local Government"[MeSH Terms] AND "public health*"[Text Word]) OR (("Government Programs"[MeSH Terms] OR "government*"[Title/Abstract]) AND "public health"[Text Word]) OR ("State Government"[MeSH Terms] AND "public health"[Text Word]) OR ("external stakeholder*"[Title/Abstract] OR (("EXTERNAL"[All Fields] OR "externally"[All Fields] OR "externals"[All Fields]) AND "stake holder*"[Title/Abstract]))) OR ("Patient Transfer"[MeSH Terms] OR "patient transfer*"[Title/Abstract] OR "resident transfer*"[Title/Abstract] OR "drug resistance, multiple"[MeSH Terms] OR "multiple drug resistan*"[Title/Abstract] OR "multi drug resistan*"[Title/Abstract] OR "multidrug resistan*"[Title/Abstract] OR "mdro*"[Title/Abstract]) OR ("Health Plan Implementation"[MeSH Terms] OR "implementation*"[Title/Abstract]) OR ("alcohol based hand rub"[Title/Abstract] OR "alcohol based hand rubs"[Title/Abstract] OR "ABHR"[Title/Abstract] OR (("Ethanol"[MeSH Terms] OR "ethanol*"[Title/Abstract]) AND "hygiene*"[Text Word])) OR ("Hand Disinfection"[MeSH Terms] OR "hand disinfection*"[Title/Abstract] OR "hand washing"[Title/Abstract] OR "HANDWASHING"[Title/Abstract] OR "hand scrubbing"[Title/Abstract] OR ("Hand Hygiene"[MeSH Terms] OR "Hand Hygiene"[Title/Abstract] OR "gloves, protective"[MeSH Terms] OR "PROTECTIVE GLOVES"[Title/Abstract])) OR ("laundry service, hospital"[MeSH Terms] OR "LAUNDRY"[Title/Abstract] OR "LAUNDRIES"[Title/Abstract] OR "Bedding and Linens"[MeSH Terms] OR "BEDDING"[Title/Abstract] OR "LINENS"[Title/Abstract]) OR ("respiratory infection*"[Title/Abstract] OR "RESPIRATORY TRACT INFECTIONS"[MeSH Terms] OR "respiratory tract infection*"[Title/Abstract] OR "respiratory protection*"[All Fields] OR "respiratory precaution*"[All Fields]) OR ("cafeteria*"[Title/Abstract] OR "dining room*"[Title/Abstract]) OR (("educat*"[Text Word] OR "ENGAGEMENT"[Title/Abstract]) AND ("family"[MeSH Terms] OR "family"[Title/Abstract] OR "FAMILIES"[Title/Abstract] OR "RELATIVES"[Title/Abstract] OR "Visitors to Patients"[MeSH Terms] OR "patient visitor*"[Title/Abstract] OR "patient s visitor*"[Title/Abstract] OR "visitors to patient*"[Title/Abstract])) OR ("Patient Care Bundles"[MeSH Terms] OR "bundle*"[Title/Abstract]) OR (("RESPIRATORY"[Text Word] OR "RESPIRATORY TRACT INFECTIONS"[MeSH Terms]) AND ("Point-of-Care Testing"[MeSH Terms] OR "point of care*"[Title/Abstract] OR "bedside test*"[Title/Abstract])) OR ("drug resistance, multiple"[MeSH Terms] OR "multiple drug resistan*"[Title/Abstract] OR "multi drug resistan*"[Title/Abstract] OR "MDRO"[Title/Abstract] OR "MDROS"[Title/Abstract] OR "multidrug resistan*"[Title/Abstract] OR ("Endemic Diseases"[MeSH Terms] OR "ENDEMIC"[Title/Abstract]) OR ("Pandemics"[MeSH Terms] OR "pandemic*"[Title/Abstract]) OR (("Disease Outbreaks"[MeSH Terms] OR "outbreak*"[Title/Abstract]) AND ("virally"[All Fields] OR "virals"[All Fields] OR "virology"[MeSH Terms] OR "virology"[All Fields] OR "viral"[All Fields] OR ("virology"[MeSH Subheading] OR "virology"[All Fields] OR "viruses"[All Fields] OR "viruses"[MeSH Terms] OR "virus s"[All Fields] OR "viruse"[All Fields] OR "virus"[All Fields])))) OR ("Patient Rights"[MeSH Terms] OR "Patient Rights"[Title/Abstract] OR "PATIENT'S RIGHTS"[Title/Abstract] OR "patients rights"[Title/Abstract] OR "resident rights"[Title/Abstract] OR "RESIDENT'S RIGHTS"[Title/Abstract] OR "residents rights"[Title/Abstract] OR "DIGNITY"[Title/Abstract]) OR ((("chlorhexidine"[MeSH Terms] OR "Tubulicid"[Title/Abstract] OR "sebidin a"[Title/Abstract] OR "MK412A"[Title/Abstract] OR "chlorhexidine"[Title/Abstract]) AND "baths"[MeSH Terms]) OR "BATH"[Title/Abstract] OR "baths"[Title/Abstract] OR "BATHING"[Title/Abstract]) OR (("NASAL"[Text Word] AND "decolonization*"[Title/Abstract]) OR "decolonisation*"[Title/Abstract]) OR ("antimicrobial stewardship"[MeSH Terms] OR "antimicrobial stewardship"[Title/Abstract] OR "ANTI-MICROBIAL STEWARDSHIP"[Title/Abstract] OR "ANTIBIOTIC STEWARDSHIP"[Title/Abstract]))

#### Sets Combined with Monitoring Concepts and Nursing Home Concepts

(((("Nursing Homes"[MeSH Terms] OR "nursing home*"[Title/Abstract] OR "intermediate care*"[Title/Abstract] OR "skilled nursing*"[Title/Abstract] OR ("SNF"[Title/Abstract] OR "SNFS"[Title/Abstract] OR "extended care facilit*"[Title/Abstract]) OR "continuing care*"[Title/Abstract] OR ("Long-Term Care"[MeSH Terms] OR "long term care*"[Title/Abstract] OR "LTC"[Title/Abstract] OR "LTCF"[Title/Abstract]) OR ("Assisted Living Facilities"[MeSH Terms] OR "ASSISTED LIVING"[Title/Abstract]) OR ("homes for the aged"[MeSH Terms] OR "homes for the aged"[Title/Abstract] OR "old age home*"[Title/Abstract] OR "HOMES FOR THE ELDERLY"[Title/Abstract]) OR "ELDER CARE"[Title/Abstract] OR "DEMENTIA CARE"[Title/Abstract] OR "MEMORY CARE"[Title/Abstract] OR ("Independent Living"[MeSH Terms] OR "independent living*"[Title/Abstract] OR "community dwelling*"[Title/Abstract]) OR ("rehabilitation center*"[Title/Abstract] OR "rehabilitation facilit*"[Title/Abstract]) OR "Rehabilitation Centers"[MeSH Terms:noexp] OR "nursing center*"[Title/Abstract] OR "Skilled Nursing Facilities"[MeSH Terms]) NOT ("animal*"[MeSH Terms] NOT ("animal*"[MeSH Terms] AND "human*"[MeSH Terms]))) NOT ("letter*"[Publication Type] OR "editorial*"[Publication Type] OR "comment*"[Publication Type])) AND "english"[Language] AND 2007/01/01:2021/12/31[Date - Publication] AND ("MONITORING"[Text Word] OR "monitoring*"[Text Word] OR "Guideline Adherence"[MeSH Terms] OR "protocol compliance"[Title/Abstract] OR "Guideline Adherence"[Title/Abstract] OR "policy compliance"[Title/Abstract] OR "audit*"[Text Word] OR "feedback"[MeSH Terms] OR "feedback"[Title/Abstract] OR "Attitude of Health Personnel"[MeSH Terms] OR "health personnel attitude*"[Title/Abstract] OR "staff attitude*"[Title/Abstract]) AND ("Hand Disinfection"[MeSH Terms] OR "hand disinfection*"[Title/Abstract] OR "hand washing"[Title/Abstract] OR "HANDWASHING"[Title/Abstract] OR "hand scrubbing"[Title/Abstract] OR ("Hand Hygiene"[MeSH Terms] OR "Hand Hygiene"[Title/Abstract]) OR ("environmental cleaning"[Title/Abstract] OR "Environmental Microbiology"[MeSH Terms:noexp] OR "environmental microbiolog*"[Title/Abstract] OR "housekeeping"[MeSH Terms] OR "housekeeping"[Title/Abstract] OR "Disinfection"[MeSH Terms] OR "disinfect*"[Title/Abstract] OR "Decontamination"[MeSH Terms] OR "decontaminat*"[Title/Abstract] OR "environmental service*"[Title/Abstract] OR "CLEANLINESS"[Title/Abstract]) OR (("Vaccination"[MeSH Terms] OR "vaccin*"[Text Word] OR "IMMUNIZATION"[MeSH Terms] OR "immunizat*"[Title/Abstract] OR "immunisat*"[Title/Abstract]) AND ("occupational health"[MeSH Terms] OR "industrial hygien*"[Title/Abstract] OR "industrial health"[Title/Abstract] OR "employee health"[Title/Abstract] OR "occupational health"[Title/Abstract])) OR ("Anti-Bacterial Agents"[MeSH Terms] OR "antibiotic*"[All Fields] OR "Antimicrobial Stewardship"[MeSH Terms] OR "antimicrobial*"[Text Word]) OR ((("midline*"[Title/Abstract] OR "PICC"[Title/Abstract] OR "PICCs"[Title/Abstract]) AND ("catheterization, central venous"[MeSH Terms] OR "central catheter*"[Title/Abstract] OR "central venous catheter*"[Title/Abstract] OR "Central Venous Catheters"[MeSH Terms] OR ("catheters, indwelling"[MeSH Terms] OR "indwelling catheter*"[Title/Abstract] OR "in dwelling catheter*"[Title/Abstract]) OR "implantable catheter*"[Title/Abstract] OR ("catheterization, peripheral"[MeSH Terms] OR "peripheral catheter*"[Title/Abstract] OR "peripherally inserted central catheter*"[Title/Abstract]))) OR "picc line*"[Title/Abstract]) OR ("enteral nutrition"[MeSH Terms] OR "enteral nutrition"[Title/Abstract] OR "force feeding*"[Title/Abstract] OR "tube feeding*"[Title/Abstract] OR "feeding tube*"[Title/Abstract]) OR ("Urinary Catheterization"[MeSH Terms] OR "urinary catheter*"[Title/Abstract] OR "foley catheter*"[Title/Abstract] OR "ureteral catheter*"[Title/Abstract] OR "urethral catheter*"[Title/Abstract] OR "Urinary Catheters"[MeSH Terms]))) OR (((("Nursing Homes"[MeSH Terms] OR "nursing home*"[Title/Abstract] OR "intermediate care*"[Title/Abstract] OR "skilled nursing*"[Title/Abstract] OR ("SNF"[Title/Abstract] OR "SNFS"[Title/Abstract] OR "extended care facilit*"[Title/Abstract]) OR "continuing care*"[Title/Abstract] OR ("Long-Term Care"[MeSH Terms] OR "long term care*"[Title/Abstract] OR "LTC"[Title/Abstract] OR "LTCF"[Title/Abstract]) OR ("Assisted Living Facilities"[MeSH Terms] OR "ASSISTED LIVING"[Title/Abstract]) OR ("homes for the aged"[MeSH Terms] OR "homes for the aged"[Title/Abstract] OR "old age home*"[Title/Abstract] OR "HOMES FOR THE ELDERLY"[Title/Abstract]) OR "ELDER CARE"[Title/Abstract] OR "DEMENTIA CARE"[Title/Abstract] OR "MEMORY CARE"[Title/Abstract] OR ("Independent Living"[MeSH Terms] OR "independent living*"[Title/Abstract] OR "community dwelling*"[Title/Abstract]) OR ("rehabilitation center*"[Title/Abstract] OR "rehabilitation facilit*"[Title/Abstract]) OR "Rehabilitation Centers"[MeSH Terms:noexp] OR "nursing center*"[Title/Abstract] OR "Skilled Nursing Facilities"[MeSH Terms]) NOT ("animal*"[MeSH Terms] NOT ("animal*"[MeSH Terms] AND "human*"[MeSH Terms]))) NOT ("letter*"[Publication Type] OR "editorial*"[Publication Type] OR "comment*"[Publication Type])) AND "english"[Language] AND 2007/01/01:2021/12/31[Date - Publication] AND ("Patient Education as Topic"[MeSH Terms:noexp] OR "PATIENT EDUCATION"[Title/Abstract] OR "education of patient*"[Title/Abstract]) AND ("Vaccination"[MeSH Terms] OR "vaccin*"[Text Word] OR ("Vaccination"[MeSH Terms] OR "IMMUNIZATION"[MeSH Terms]) OR "immunizat*"[Title/Abstract] OR "immunisat*"[Title/Abstract] OR "Hand Disinfection"[MeSH Terms] OR "hand disinfection*"[Title/Abstract] OR "hand washing"[Title/Abstract] OR "HANDWASHING"[Title/Abstract] OR "hand scrubbing"[Title/Abstract] OR "Hand Hygiene"[MeSH Terms] OR "Hand Hygiene"[Title/Abstract] OR "respiratory tract infections/prevention and control"[MeSH Terms] OR "respiratory protection*"[Title/Abstract] OR "respiratory precaution*"[Title/Abstract] OR "Personal Protective Equipment"[MeSH Terms] OR "personal protective equipment*"[Title/Abstract] OR "PPE"[Title/Abstract] OR "Anti-Bacterial Agents"[MeSH Terms] OR "antibiotic*"[Text Word] OR "Antimicrobial Stewardship"[MeSH Terms] OR "antimicrobial*"[Text Word] OR "Disease Outbreaks"[MeSH Terms] OR "OUTBREAK"[Text Word])) OR (("presenteeism"[MeSH Terms] OR "presenteeism"[Title/Abstract] OR "SICKNESS PRESENCE"[Title/Abstract]) AND (((("Nursing Homes"[MeSH Terms] OR "nursing home*"[Title/Abstract] OR "intermediate care*"[Title/Abstract] OR "skilled nursing*"[Title/Abstract] OR ("SNF"[Title/Abstract] OR "SNFS"[Title/Abstract] OR "extended care facilit*"[Title/Abstract]) OR "continuing care*"[Title/Abstract] OR ("Long-Term Care"[MeSH Terms] OR "long term care*"[Title/Abstract] OR "LTC"[Title/Abstract] OR "LTCF"[Title/Abstract]) OR ("Assisted Living Facilities"[MeSH Terms] OR "ASSISTED LIVING"[Title/Abstract]) OR ("homes for the aged"[MeSH Terms] OR "homes for the aged"[Title/Abstract] OR "old age home*"[Title/Abstract] OR "HOMES FOR THE ELDERLY"[Title/Abstract]) OR "ELDER CARE"[Title/Abstract] OR "DEMENTIA CARE"[Title/Abstract] OR "MEMORY CARE"[Title/Abstract] OR ("Independent Living"[MeSH Terms] OR "independent living*"[Title/Abstract] OR "community dwelling*"[Title/Abstract]) OR ("rehabilitation center*"[Title/Abstract] OR "rehabilitation facilit*"[Title/Abstract]) OR "Rehabilitation Centers"[MeSH Terms:noexp] OR "nursing center*"[Title/Abstract] OR "Skilled Nursing Facilities"[MeSH Terms]) NOT ("animal*"[MeSH Terms] NOT ("animal*"[MeSH Terms] AND "human*"[MeSH Terms]))) NOT ("letter*"[Publication Type] OR "editorial*"[Publication Type] OR "comment*"[Publication Type])) AND "english"[Language] AND 2007/01/01:2021/12/31[Date - Publication])) OR (((("Nursing Homes"[MeSH Terms] OR "nursing home*"[Title/Abstract] OR "intermediate care*"[Title/Abstract] OR "skilled nursing*"[Title/Abstract] OR ("SNF"[Title/Abstract] OR "SNFS"[Title/Abstract] OR "extended care facilit*"[Title/Abstract]) OR "continuing care*"[Title/Abstract] OR ("Long-Term Care"[MeSH Terms] OR "long term care*"[Title/Abstract] OR "LTC"[Title/Abstract] OR "LTCF"[Title/Abstract]) OR ("Assisted Living Facilities"[MeSH Terms] OR "ASSISTED LIVING"[Title/Abstract]) OR ("homes for the aged"[MeSH Terms] OR "homes for the aged"[Title/Abstract] OR "old age home*"[Title/Abstract] OR "HOMES FOR THE ELDERLY"[Title/Abstract]) OR "ELDER CARE"[Title/Abstract] OR "DEMENTIA CARE"[Title/Abstract] OR "MEMORY CARE"[Title/Abstract] OR ("Independent Living"[MeSH Terms] OR "independent living*"[Title/Abstract] OR "community dwelling*"[Title/Abstract]) OR ("rehabilitation center*"[Title/Abstract] OR "rehabilitation facilit*"[Title/Abstract]) OR "Rehabilitation Centers"[MeSH Terms:noexp] OR "nursing center*"[Title/Abstract] OR "Skilled Nursing Facilities"[MeSH Terms]) NOT ("animal*"[MeSH Terms] NOT ("animal*"[MeSH Terms] AND "human*"[MeSH Terms]))) NOT ("letter*"[Publication Type] OR "editorial*"[Publication Type] OR "comment*"[Publication Type])) AND "english"[Language] AND 2007/01/01:2021/12/31[Date - Publication] AND ((("occupational health"[MeSH Terms] AND "employee*"[Text Word]) OR "occupational health"[Title/Abstract] OR "health workforce"[MeSH Terms] OR "workforce*"[Title/Abstract] OR "Health Personnel"[MeSH Terms] OR "PERSONNEL"[Text Word] OR "MANPOWER"[Title/Abstract] OR "staff*"[Text Word] OR "occupational groups"[MeSH Terms] OR "worker*"[Title/Abstract] OR "Health Personnel"[MeSH Terms:noexp] OR "pharmacist*"[Text Word] OR "dentist*"[Text Word] OR "nurses"[MeSH Terms] OR "NURSE"[Text Word] OR "nurses"[Text Word] OR "Nursing Assistants"[MeSH Terms] OR "nursing assistant*"[Title/Abstract] OR "nurses aides"[Title/Abstract] OR "NURSING STAFF"[MeSH Terms] OR "Allied Health Personnel"[MeSH Terms:noexp] OR "Medical Staff"[MeSH Terms:noexp] OR "physician*"[Text Word]) AND ("Vaccination"[MeSH Terms] OR "vaccin*"[Text Word] OR "IMMUNIZATION"[MeSH Terms] OR "immunizat*"[Title/Abstract] OR "immunisat*"[Title/Abstract]))) OR (("national healthcare safety network"[Title/Abstract] OR "NHSN"[Title/Abstract]) AND (((("Nursing Homes"[MeSH Terms] OR "nursing home*"[Title/Abstract] OR "intermediate care*"[Title/Abstract] OR "skilled nursing*"[Title/Abstract] OR ("SNF"[Title/Abstract] OR "SNFS"[Title/Abstract] OR "extended care facilit*"[Title/Abstract]) OR "continuing care*"[Title/Abstract] OR ("Long-Term Care"[MeSH Terms] OR "long term care*"[Title/Abstract] OR "LTC"[Title/Abstract] OR "LTCF"[Title/Abstract]) OR ("Assisted Living Facilities"[MeSH Terms] OR "ASSISTED LIVING"[Title/Abstract]) OR ("homes for the aged"[MeSH Terms] OR "homes for the aged"[Title/Abstract] OR "old age home*"[Title/Abstract] OR "HOMES FOR THE ELDERLY"[Title/Abstract]) OR "ELDER CARE"[Title/Abstract] OR "DEMENTIA CARE"[Title/Abstract] OR "MEMORY CARE"[Title/Abstract] OR ("Independent Living"[MeSH Terms] OR "independent living*"[Title/Abstract] OR "community dwelling*"[Title/Abstract]) OR ("rehabilitation center*"[Title/Abstract] OR "rehabilitation facilit*"[Title/Abstract]) OR "Rehabilitation Centers"[MeSH Terms:noexp] OR "nursing center*"[Title/Abstract] OR "Skilled Nursing Facilities"[MeSH Terms]) NOT ("animal*"[MeSH Terms] NOT ("animal*"[MeSH Terms] AND "human*"[MeSH Terms]))) NOT ("letter*"[Publication Type] OR "editorial*"[Publication Type] OR "comment*"[Publication Type])) AND "english"[Language] AND 2007/01/01:2021/12/31[Date - Publication])) OR (("transmission based precaution*"[Text Word] OR "droplet precaution*"[Title/Abstract] OR "contact precaution*"[Title/Abstract] OR (((("ultrafine"[All Fields] OR "ultrafines"[All Fields]) AND "fiber*"[All Fields]) OR "airborn*"[Text Word] OR ("PARTICULATE MATTER"[MeSH Terms] OR ("particulate"[All Fields] AND "matter"[All Fields]) OR "PARTICULATE MATTER"[All Fields] OR ("particulate"[All Fields] AND "air"[All Fields] AND "pollutants"[All Fields]) OR "particulate air pollutants"[All Fields]) OR ("PARTICULATE MATTER"[MeSH Terms] OR ("particulate"[All Fields] AND "matter"[All Fields]) OR "PARTICULATE MATTER"[All Fields] OR ("ambient"[All Fields] AND "particulate"[All Fields] AND "matter"[All Fields]) OR "ambient particulate matter"[All Fields]) OR ("PARTICULATE MATTER"[MeSH Terms] OR ("particulate"[All Fields] AND "matter"[All Fields]) OR "PARTICULATE MATTER"[All Fields] OR ("ultrafine"[All Fields] AND "particulate"[All Fields] AND "matter"[All Fields]) OR "ultrafine particulate matter"[All Fields]) OR (("ultrafine"[All Fields] OR "ultrafines"[All Fields]) AND "particle*"[All Fields]) OR "PARTICULATE MATTER"[MeSH Terms]) AND "precaution*"[Title/Abstract])) AND (((("Nursing Homes"[MeSH Terms] OR "nursing home*"[Title/Abstract] OR "intermediate care*"[Title/Abstract] OR "skilled nursing*"[Title/Abstract] OR ("SNF"[Title/Abstract] OR "SNFS"[Title/Abstract] OR "extended care facilit*"[Title/Abstract]) OR "continuing care*"[Title/Abstract] OR ("Long-Term Care"[MeSH Terms] OR "long term care*"[Title/Abstract] OR "LTC"[Title/Abstract] OR "LTCF"[Title/Abstract]) OR ("Assisted Living Facilities"[MeSH Terms] OR "ASSISTED LIVING"[Title/Abstract]) OR ("homes for the aged"[MeSH Terms] OR "homes for the aged"[Title/Abstract] OR "old age home*"[Title/Abstract] OR "HOMES FOR THE ELDERLY"[Title/Abstract]) OR "ELDER CARE"[Title/Abstract] OR "DEMENTIA CARE"[Title/Abstract] OR "MEMORY CARE"[Title/Abstract] OR ("Independent Living"[MeSH Terms] OR "independent living*"[Title/Abstract] OR "community dwelling*"[Title/Abstract]) OR ("rehabilitation center*"[Title/Abstract] OR "rehabilitation facilit*"[Title/Abstract]) OR "Rehabilitation Centers"[MeSH Terms:noexp] OR "nursing center*"[Title/Abstract] OR "Skilled Nursing Facilities"[MeSH Terms]) NOT ("animal*"[MeSH Terms] NOT ("animal*"[MeSH Terms] AND "human*"[MeSH Terms]))) NOT ("letter*"[Publication Type] OR "editorial*"[Publication Type] OR "comment*"[Publication Type])) AND "english"[Language] AND 2007/01/01:2021/12/31[Date - Publication]))

### Embase

1 cross infection/ and prevent*.mp. [mp=title, abstract, heading word, drug trade name, original title, device manufacturer, drug manufacturer, device trade name, keyword, floating subheading word, candidate term word] 11774

2 (cross infection* and prevent*).ti,ab,kw. 1151

3 (nosocomial infection* and prevent*).ti,ab,kw. 5866

4 healthcare associated infection/ and prevent*.mp. [mp=title, abstract, heading word, drug trade name, original title, device manufacturer, drug manufacturer, device trade name, keyword, floating subheading word, candidate term word] 2487

5 ("health care associated infection*" and prevent*).ti,ab,kw. 1108

6 (bacterial transmission/ or virus transmission/) and prevent*.mp. [mp=title, abstract, heading word, drug trade name, original title, device manufacturer, drug manufacturer, device trade name, keyword, floating subheading word, candidate term word] 29741

7 ((infectio* adj3 disease transmission) and prevent*).mp. [mp=title, abstract, heading word, drug trade name, original title, device manufacturer, drug manufacturer, device trade name, keyword, floating subheading word, candidate term word] 370

8 infection/pc [Prevention] 7834

9 infection control/ 90355

10 (infection* adj3 (prevent* or control* or reduc*)).mp. [mp=title, abstract, heading word, drug trade name, original title, device manufacturer, drug manufacturer, device trade name, keyword, floating subheading word, candidate term word] 239823

11 nursing home/ 54410

12 "long term care".ti,ab,kw. 28874

13 nursing home*.ti,ab,kw. 41472

14 (intermediate care facilit* or skilled nursing facilit* or extended care facilit* or continuing care facilit*).ti,ab,kw. 6035

15 assisted living facility/ 2712

16 assisted living facilit*.ti,ab,kw. 1181

17 home for the aged/ 11124

18 "homes for the aged".ti,ab,kw. 737

19 "old age home*".ti,ab,kw. 392

20 "homes for the elderly".ti,ab,kw. 886

21 "dementia care".ti,ab,kw. 4123

22 "memory care".ti,ab,kw. 135

23 community dwelling*.ti,ab,kw. 33023

24 residential home/ 7273

25 (group home* or residential facilit* or residential institution*).ti,ab,kw. 2993

26 rehabilitation center/ 15564

27 REHABILITATION CENTER*.ti,ab,kw. 6526

28 NURSING CENTER*.ti,ab,kw. 318

29 REHABILITATION FACILIT*.ti,ab,kw. 4873

30 or/11-29 157284

31 limit 30 to english language 144817

32 limit 31 to abstracts 118461

33 exp ANIMAL/ 27019372

34 exp HUMAN/ 22242750

35 33 not (33 and 34) 4776622

36 32 not 35 118027

37 limit 36 to (embase and (article or article in press or "review")) 59221

38 limit 37 to dc=20070101-20210515 39182

39 or/1-10 270988

40 38 and 39 1102

41 leadership/ 73549

42 LEADER*.ti,ab,kw. 99072

43 "organization and management"/ 420582

44 manager/ 23920

45 medical director/ 1361

46 "MEDICAL DIRECTOR*".ti,ab,kw. 4928

47 ("PHYSICIAN EXECUTIVE*" or "NURSE EXECUTIVE*").ti,ab,kw. 1817

48 nurse administrator/ 12761

49 "NURSE MANAGER*".ti,ab,kw. 3969

50 infection control practitioner/ 433

51 (INFECTION CONTROL PRACTITIONER* or INFECTION PREVENTIONIST*).ti,ab,kw. 1292

52 or/41-51 559059

53 risk assessment/ 611735

54 (RISK ASSESSMENT* or RISK ANALYS*).ti,ab,kw. 114419

55 risk factor/ 1116330

56 (RISK FACTOR* or RISK SCOR* or HEALTH CORRELATE*).ti,ab,kw. 991092

57 RISK EVALUAT*.ti,ab,kw. 7793

58 RISK MITIGAT*.ti,ab,kw. 2164

59 or/53-58 1944130

60 HEALTH RESOURCE*.ti,ab,kw. 9727

61 health workforce/ 1805

62 WORKFORCE*.ti,ab,kw. 33361

63 health care personnel/ or acupuncturist/ or advanced practice provider/ or anesthesist/ or care coordinator/ or clinician/ or dental personnel/ or epidemiologist/ or eye care professional/ or health auxiliary/ or health educator/ or lay health worker/ or medical personnel/ or medical scribe/ or mental health care personnel/ or nursing home personnel/ or orthotist/ or paramedical personnel/ or perfusionist/ or prosthetist/ or traditional healer/ 259378

64 PERSONNEL.ti,ab,kw. 98784

65 MANPOWER.ti,ab,kw. 9367

66 ((PERSONNEL or STAFF* or WORKER* or LABOR or NURS* or MANPOWER) adj3 (TURNOVER or STABILIT* or STABLE or INSTABILIT*)).mp. [mp=title, abstract, heading word, drug trade name, original title, device manufacturer, drug manufacturer, device trade name, keyword, floating subheading word, candidate term word] 3444

67 funding/ 52322

68 financial support*.ti,ab,kw. 7683

69 financial investment*.ti,ab,kw. 747

70 devices/ 106488

71 (equipment or supplies).ti,ab,kw. 153995

72 protective equipment/ 20278

73 (personal protective equipment or PPE).ti,ab,kw. 10809

74 HEALTH CARE EVALUATION*.mp. 359

75 professional competence/ 32671

76 clinical competence/ 62752

77 job performance/ 17107

78 (WORK PERFORMANCE or JOB PERFORMANCE).ti,ab,kw. 5113

79 EXPERTISE.mp. 68370

80 or/60-79 857684

81 sentinel surveillance/ 2526

82 surveillance*.ti,ab,kw. 270124

83 (epidemic/ or epidemic*.ti,ab,kw. or disease outbreak*.ti,ab,kw.) and (detect* or reporting).ti,ab,kw. 27550

84 mandatory reporting/ or mandatory report*.ti,ab,kw. 4121

85 ("centers for medicare and medicaid" or "centers for disease control and prevention").mp. and reporting.ti,ab,kw. 1618

86 exp health care quality/ 3436833

87 qapi.ti,ab. 94

88 exp quality control/ 432610

89 quality assessment*.ti,ab,kw. 30941

90 audit*.ti,ab,kw. 220063

91 in service training/ 15707

92 ("in service training" or "inservice training").ti,ab,kw. 1992

93 staff development.ti,ab,kw. 2036

94 ((educat* or TRAINING) and (staff* or personnel or physician* or DOCTOR* or nurs* or allied health* or worker* or MANPOWER or WORKFORCE or WORK FORCE or LABOR or EMPLOYEE*)).mp. 626735

95 MECHANICAL VENTILATOR/ or (VENTILATOR* or VENTILATION SERVICE* or RESPIRATOR or RESPIRATORS).ti,ab,kw. 90420

96 exp dialysis/ 196122

97 DIALYS*.ti,ab,kw. 171908

98 or/81-97 4853017

99 organizational policy/ 1531

100 ((organization* or facilit* or institution*) adj3 (policy or policies)).mp. [mp=title, abstract, heading word, drug trade name, original title, device manufacturer, drug manufacturer, device trade name, keyword, floating subheading word, candidate term word] 9098

101 exp occupational health/ 236040

102 (employee health or occupational health).ti,ab,kw. 22020

103 (infrastructure or physical plant*).ti,ab,kw. 48172

104 FACILITY CHARACTERISTIC*.ti,ab,kw. 1086

105 ("patients rooms" or "PATIENT'S ROOM*" or "PATIENT ROOMS").ti,ab,kw. 1680

106 water quality/ 43177

107 water supply/ 39553

108 (water qualit* or Water Supply or potable water or facility water or water system*).ti,ab,kw. 49475

109 airflow/ 12258

110 (air flow or airflow).ti,ab,kw. 30301

111 air conditioning/ 23194

112 air conditioning*.ti,ab,kw. 2785

113 air movement*.ti,ab,kw. 503

114 indoor air pollution/ 13798

115 exp microclimate/ 52059

116 (indoor air pollution or indoor air qualit*).ti,ab,kw. 5277

117 (air borne or airborne).mp. 33932

118 or/99-117 499858

119 exp environmental monitoring/ 107560

120 (environmental monitor* or environmental surveillance* or environmental design).ti,ab,kw. 8485

121 medical device contamination/ 1044

122 ((equipment or medical device*) adj3 contaminat*).mp. [mp=title, abstract, heading word, drug trade name, original title, device manufacturer, drug manufacturer, device trade name, keyword, floating subheading word, candidate term word] 2355

123 (environment contamination or environmental contamination).ti,ab,kw. 5975

124 needs assessment/ 25353

125 (need assessment* or needs assessment*).ti,ab,kw. 11752

126 consultation/ 118665

127 (consultation* or consultant*).ti,ab,kw. 168661

128 local government*.ti,ab,kw. 6923

129 government program*.ti,ab,kw. 858

130 state government*.ti,ab,kw. 2202

131 government/ and health.mp. 96064

132 patient transport/ 28037

133 (patient transfer* or patient transport*).ti,ab,kw. 4111

134 multidrug resistance/ 48824

135 (multiple drug resistan* or multi drug resistan* or multidrug resistan* or mdro* or CORHA).ti,ab,kw. 86414

136 implementation*.ti,ab,kw. 370354

137 antiinfective agent/ 183587

138 antibiotic agent/ 321436

139 antimicrobial stewardship/ 6081

140 (antiinfective* or anti infective* or antibiotic* or antimicrobial* or anti microbial*).ti,ab,kw. 650481

141 or/119-140 1734839

142 (alcohol based hand rub* or abhr).ti,ab,kw. 758

143 hand disinfection/ 601

144 hand washing/ 16216

145 (hand disinfection* or hand washing or HANDWASHING or hand scrubbing or Hand Hygiene).ti,ab,kw. 13728

146 protective glove/ 889

147 protective glove*.ti,ab,kw. 572

148 laundry/ 3057

149 (laundry or laundries or launder*).ti,ab,kw. 3395

150 (linens or bedding).ti,ab,kw. 4319

151 respiratory tract infection/ and prevent*.mp. 10103

152 ((respiratory infection* or respiratory tract infection*) and prevention*).mp. [mp=title, abstract, heading word, drug trade name, original title, device manufacturer, drug manufacturer, device trade name, keyword, floating subheading word, candidate term word] 16427

153 (RESPIRATORY PROTECTION* or RESPIRATORY PRECAUTION*).ti,ab,kw. 1190

154 (dining room* or cafeteria*).ti,ab,kw. 2997

155 patient education/ 116564

156 (patient education or "education of patient").ti,ab,kw. 34282

157 or/142-156 179329

158 family/ 94073

159 patient visitor/ 384

160 relative/ 15178

161 (family or families or relatives or visitor*).ti,ab,kw. 1310493

162 (158 or 159 or 160 or 161) and (educat* or engagement).mp. 147077

163 presenteeism/ 1570

164 "working while sick".ti,ab,kw. 36

165 "working while ill".ti,ab,kw. 36

166 sickness presence.ti,ab,kw. 49

167 national healthcare safety network.ti,ab,kw. 1196

168 care bundle/ 1509

169 bundle*.ti,ab,kw. 87789

170 or/162-169 237447

171 "point of care testing"/ 15371

172 ("point of care testing" or bedside testing).ti,ab,kw. 5948

173 exp respiratory tract infection/ 426917

174 (Respiratory tract infection* or respiratory infection*).ti,ab,kw. 66268

175 (171 or 172) and (173 or 174) 960

176 multidrug resistance/ 48824

177 (multiple drug resistan* or multi drug resistan* or MDRO or MDROS or multidrug resistan*).ti,ab,kw. 86407

178 endemic disease/ 22376

179 ENDEMIC.ti,ab,kw. 105487

180 (Outbreak* and (viral or virus*)).ti,ab,kw. 38281

181 or/175-180 248313

182 transmission based precaution*.ti,ab,kw. 164

183 droplet precaution*.ti,ab,kw. 166

184 contact precaution*.ti,ab,kw. 1278

185 airborne.mp. 33077

186 exp patient right/ 171057

187 human dignity/ 3808

188 (Patient Rights or patients rights or resident rights or residents rights or DIGNITY).ti,ab,kw. 12122

189 (chlorhexidine and bath*).mp. 835

190 (nasal decoloniz* or nasal decolonis*).ti,ab,kw. 124

191 or/182-190 216633

192 monitoring.ti,ab. 729990

193 protocol compliance/ 15640

194 clinical audit/ 5695

195 health personnel attitude/ 82260

196 (Guideline Adherence or PROTOCOL COMPLIANCE or POLICY COMPLIANCE or audit* or feedback or health personnel attitude* or staff attitude*).ti,ab,kw. 407298

197 or/192-196 1208895

198 environmental microbiology/ 682

199 housekeeping/ 1039

200 disinfection/ 27820

201 decontamination/ 3354

202 environmental service*.ti,ab,kw. 761

203 cleanliness.ti,ab,kw. 4152

204 (environmental cleaning or environmental microbiolog* or housekeeping or disinfect* or decontaminat*).ti,ab,kw. 67062

205 ((Vaccinat* or vaccini* or IMMUNI*) and (occupational health or employee* or staff or personnel or worker* or workforce)).mp. [mp=title, abstract, heading word, drug trade name, original title, device manufacturer, drug manufacturer, device trade name, keyword, floating subheading word, candidate term word] 23088

206 midline catheter/ 152

207 peripherally inserted central venous catheter/ 3760

208 (PICC or PICCS).ti,ab,kw. 3444

209 MIDLINE CATHETER*.ti,ab,kw. 223

210 central venous catheter/ 22206

211 indwelling catheter/ 11716

212 (central catheter* or central venous catheter* or indwelling catheter* or in dwelling catheter* or implantable catheter* or peripherally inserted central catheter*).ti,ab,kw. 29245

213 enteric feeding/ 34071

214 (enteral feeding or force feeding* or tube feeding* or feeding tube*).ti,ab,kw. 19377

215 exp bladder catheterization/ 9457

216 urinary catheter/ 6134

217 (urinary catheter* or foley catheter* or ureteral catheter* or urethral catheter*).ti,ab,kw. 18092

218 or/143-147 22635

219 or/198-217 218277

220 38 and 197 and (218 or 219) 106

221 40 and (52 or 59 or 80 or 98 or 118 or 141 or 157 or 170 or 181 or 191) 1003

222 220 or 221 1059

## Exclusion criteria

Expert guidance documents and Compendium documents are not GRADE-based. Exclusions generally are based on determination that the article in question meets one or more of the following criteria:

Standard Criteria:

- Wrong language OR not available
- Not relevant to topic i.e.: setting, treatment, disease, diagnostic process, comorbidity, age group, sample size,
- lab study, sex, duration, intervention, procedure, administration route
- Insufficient: intervention data, randomization, control, design, data analysis
- Data reported already in another study

Consensus for exclusion as determined by authors:

- Papers on plans to implement an intervention (determined to be too premature to include)
- Predictors
- Descriptions of knowledge or attitudes that are both based on a small sample size and are unrelated to infection prevention in nursing homes

## Preferred reporting items for systematic reviews and meta-analyses (PRISMA)

| **Identification** | **May 2021** |  | |
| --- | --- | --- | --- |
|  | **PubMed** (5/14): 3,100 **Embase** (5/18): 1,059 |  | |
|  | Total: 4,159 | Duplicates removed: 810 |  |
|  | Number of records after duplicates removed: 3,349 | Number of records excluded by librarian based on exclusion criteria: 695 | |
|  |  |  |  |
| **Screening** | Number of records delivered by librarian  and imported to Covidence: 2,654 | Number of records excluded (2 reviewers per study; conflicts resolved by RM and LM): 1,915 | |
|  |  |  |  |
| **Eligibility** | Number of records assessed by full text: 737 |  | |
|  |  |  |  |
|  |  |  |  |
| **Identification** (Update) | **Jan. 2024** |  | |
|  | **PubMed** (1/18): 1,825 | Duplicates removed: 235 | |
|  | Total after duplicates removed: 1,590 | Number of records excluded by librarian with exclusion criteria: 1,493 | |
| **Screening** | Number of records screened by panel: 98 | Number of records excluded: 70 | |
|  |  |  |  |
| **Eligibility** | Number of records assessed by full text: 28 |  | |
|  |  |  |  |
| **Extraction** | Number of records extracted: 28 |  | |
|  |  |  |  |
| **Included** | Total number of records included in final manuscript: 239 |  | |
|  |  |  |  |

# References

1. Centers for Disease Control and Prevention. The Core Elements of Antibiotic Stewardship for Nursing Homes. US Department of Health and Human Services Division of Healthcare Quality Promotion; 2017. Updated 2024.

2. Salem-Schatz S, Griswold P, Kandel R, et al. A Statewide Program to Improve Management of Suspected Urinary Tract Infection in Long-Term Care. J Am Geriatr Soc. 2020;68:62-9.

3. Felsen CB, Dodds Ashley ES, Barney GR, et al. Reducing Fluoroquinolone Use and Clostridioides difficile Infections in Community Nursing Homes Through Hospital-Nursing Home Collaboration. J Am Med Dir Assoc. 2020;21:55-61.e2.

4. Sloane PD, Zimmerman S, Ward K, et al. A 2-Year Pragmatic Trial of Antibiotic Stewardship in 27 Community Nursing Homes. J Am Geriatr Soc. 2020;68:46-54.

5. Stevenson LD, Banks RE, Stryczek KC, et al. A pilot study using telehealth to implement antimicrobial stewardship at two rural Veterans Affairs medical centers. Infect Control Hosp Epidemiol. 2018;39:1163-9.

6. Doernberg SB, Dudas V, Trivedi KK. Implementation of an antimicrobial stewardship program targeting residents with urinary tract infections in three community long-term care facilities: a quasi-experimental study using time-series analysis. Antimicrob Resist Infect Control. 2015;4:54.

7. Zimmerman S, Sloane PD, Bertrand R, Olsho LE, Beeber A, Kistler C, et al. Successfully reducing antibiotic prescribing in nursing homes. J Am Geriatr Soc. 2014;62:907-12.

8. Jump RL, Olds DM, Seifi N, et al. Effective antimicrobial stewardship in a long-term care facility through an infectious diseases consultation service: keeping a LID on antibiotic use. Infect Control Hosp Epidemiol. 2012;33:1185-92.

9. Miller LG, McKinnell JA, Singh RD, et al. Decolonization in Nursing Homes to Prevent Infection and Hospitalization. N Engl J Med. 2023;389:1766-77.

10. Gussin GM, McKinnell JA, Singh RD, et al. Reducing Hospitalizations and Multidrug-Resistant Organisms via Regional Decolonization in Hospitals and Nursing Homes. JAMA. 2024;331:1544-57.

11. Zimmerman S, Sloane PD, Ward K, et al. Effectiveness of a Mouth Care Program Provided by Nursing Home Staff vs Standard Care on Reducing Pneumonia Incidence: A Cluster Randomized Trial. JAMA Netw Open. 2020;3:e204321.

12. Weintraub JA, Zimmerman S, Ward K, et al. Improving Nursing Home Residents' Oral Hygiene: Results of a Cluster Randomized Intervention Trial. J Am Med Dir Assoc. 2018;19:1086-91.

13. Mody L, Kauffman CA, McNeil SA, et al. Mupirocin-based decolonization of Staphylococcus aureus carriers in residents of 2 long-term care facilities: a randomized, double-blind, placebo-controlled trial. Clin Infect Dis. 2003;37:1467-74.

14. Juthani-Mehta M, Van Ness PH, Bianco L, et al. Effect of Cranberry Capsules on Bacteriuria Plus Pyuria Among Older Women in Nursing Homes: A Randomized Clinical Trial. Jama. 2016;316:1879-87.
